# Redox Dynamics of Active VO*_x_* Sites Promoted by TiO*_x_* during Oxidative Dehydrogenation of Ethanol Detected by *Operando* Quick XAS

**DOI:** 10.1021/jacsau.2c00027

**Published:** 2022-03-14

**Authors:** Anna Zabilska, Adam H. Clark, Benjamin M. Moskowitz, Israel E. Wachs, Yuya Kakiuchi, Christophe Copéret, Maarten Nachtegaal, Oliver Kröcher, Olga V. Safonova

**Affiliations:** † Paul Scherrer Institute, 5232 Villigen, Switzerland; ‡ École Polytechnique Fédérale de Lausanne, 1015 Lausanne, Switzerland; § *Operando* Molecular Spectroscopy & Catalysis Laboratory, Department of Chemical & Biomolecular Engineering, Lehigh University, Bethlehem, Pennsylvania 18015, United States; ∥ Department of Chemistry and Applied Biosciences, ETH Zürich, CH-8093 Zürich, Switzerland

**Keywords:** operando XAS, V K-edge XAS, time-resolved XAS, oxidative dehydrogenation, titania-supported vanadia

## Abstract

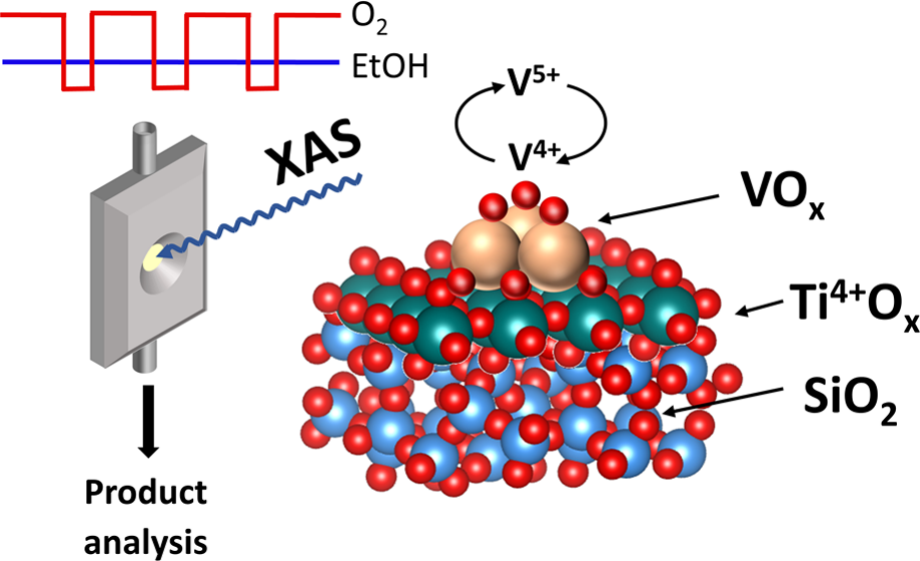

Titania-supported vanadia (VO*_x_*/TiO_2_) catalysts exhibit outstanding catalytic in a number of selective oxidation and reduction processes. In spite of numerous investigations, the nature of redox transformations of vanadium and titanium involved in various catalytic processes remains difficult to detect and correlate to the rate of products formation. In this work, we studied the redox dynamics of active sites in a bilayered 5% V_2_O_5_/15% TiO_2_/SiO_2_ catalyst (consisting of submonolayer VO*_x_* species anchored onto a TiO*_x_* monolayer, which in turn is supported on SiO_2_) during the oxidative dehydrogenation of ethanol. The VO*_x_* species in 5% V_2_O_5_/15% TiO_2_/SiO_2_ show high selectivity to acetaldehyde and an ca. 40 times higher acetaldehyde formation rate in comparison to VO*_x_* species supported on SiO_2_ with a similar density. *Operando* time-resolved V and Ti K-edge X-ray absorption near-edge spectroscopy, coupled with a transient experimental strategy, quantitatively showed that the formation of acetaldehyde over 5% V_2_O_5_/15% TiO_2_/SiO_2_ is kinetically coupled to the formation of a V^4+^ intermediate, while the formation of V^3+^ is delayed and 10–70 times slower. The low-coordinated nature of various redox states of VO*_x_* species (V^5+^, V^4+^, and V^3+^) in the 5% V_2_O_5_/15% TiO_2_/SiO_2_ catalyst is confirmed using the extensive database of V K-edge XANES spectra of standards and specially synthesized molecular crystals. Much weaker redox activity of the Ti^4+^/Ti^3+^ couple was also detected; however, it was found to not be kinetically coupled to the rate-determining step of ethanol oxidation. Thus, the promoter effect of TiO*_x_* is rather complex. TiO*_x_* species might be involved in a fast electron transport between VO*_x_* species and might affect the electronic structure of VO*_x_*, thereby promoting their reducibility. This study demonstrates the high potential of element-specific *operando* X-ray absorption spectroscopy for uncovering complex catalytic mechanisms involving the redox kinetics of various metal oxides.

## Introduction

Supported vanadia (VO*_x_*) materials belong to some of the most versatile selective oxidation catalysts applied for many reactions in the chemical industry and pollution control. For example, VO*_x_* species show high activity in oxidative dehydrogenation (ODH) of short alkanes and alcohols to the corresponding alkenes and aldehydes, respectively,^1,2^ oxidation of *o*-xylene to phthalic anhydride,^3^ oxidation of *n*-butane to maleic anhydride,^4^ oxidation of sulfur dioxide to sulfur trioxide,^5^ ammoxidation of alkylaromatics,^6^ and selective catalytic reduction of nitrogen oxides with ammonia.^7^ The ODH of alcohols is particularly attractive since it is one of the main industrial methods of formaldehyde production and as a possible route for the transformation of renewable bioethanol to value-added products, such as acetaldehyde and acetic acid.

**Scheme 1:**
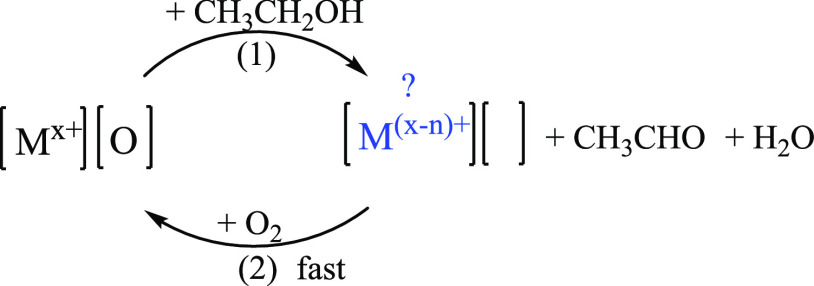
MvK Mechanism of Ethanol Oxidation The oxidation of an alcohol by lattice oxygen ([O]) accompanied by the formation of a partially reduced metal intermediate (M^(*x*–*n*)+^) and an oxygen vacancy ([ ]), and is followed by the oxidation of the reduced intermediate by molecular oxygen resulting in the regeneration of the oxidized metal species (M^*x*+^).

This reaction proceeds via the Mars–van Krevelen (MvK) reaction mechanism^8−11^ as depicted in Scheme 1.

In the MvK mechanism, the oxidation of an alcohol takes place on the catalyst surface and is accompanied by the formation of a partially reduced metal species (intermediate M^(*x*–*n*)+^) and oxygen vacancy ([ ]). This step is followed by the reoxidation of the reduced intermediate (M^(*x*–*n*)+^) by molecular oxygen resulting in the regeneration of the oxidized metal species (M^*x*+^). By comparing the catalytic oxidation rates of normal and D-labeled ethanol (EtOH) over supported vanadia catalysts, it was found that the rate-determining step of alcohol oxidation is kinetically coupled to the abstraction of hydrogen from the α-carbon of an alcohol.^12−14^ The oxidation of the partially reduced metal intermediate (M^(*x*–*n*)+^) by molecular oxygen (Step 2 in Scheme 1) is assumed to be very rapid^8,9,12,15,16^ and, therefore, the concentration of the M^(*x*–*n*)+^ intermediate during the ODH process should be low.

The mechanism of alcohol oxidation over supported VO*_x_* species was examined by many groups using multiple *in situ* spectroscopic techniques (Fourier transform infrared (FT-IR) spectroscopy,^2,9,15−19^ Raman spectroscopy,^11,18,20^ diffuse reflectance UV–vis (DR UV–vis) spectroscopy,^18,20^ X-ray photoelectron spectroscopy (XPS),^9,15,17^ and X-ray absorption spectroscopy (XAS)^19,21^). Although several reaction mechanisms were proposed based on the nature of the observed surface reaction intermediates, the redox activity of vanadium and titanium species is difficult to assess under *operando* conditions and in a quantitative manner. Kaichev et al. studied methanol and ethanol oxidation over supported VO*_x_*/TiO_2_ catalysts with ambient pressure (AP) XPS and found that both V^3+^ and V^4+^ are formed on the surface during interaction with alcohols at relevant temperatures.^9,15,17^ These experiments, however, were performed at 0.25–0.5 mbar pressure, which is far removed from industrial conditions at atmospheric pressure. Wu et al.^22^ investigated supported VO*_x_*/Al_2_O_3_ catalyst after exposure to methanol with XPS under UHV conditions and detected V^3+^ and V^4+^ species. Vieira et al.^19^ investigated zeolite-supported vanadia catalysts in the methanol ODH by *in situ* time-resolved V K-edge XAS and concluded that V^4+^ is the main intermediate involved in the reaction. The observed rate of V^4+^ reoxidation by oxygen, however, was slower than V^5+^ reduction by methanol, which contradicts the literature and might be related to the large volume of the *in situ* cell, which is not ideal for kinetic studies. Moreover, linear combination fitting of the V K-edge XAS spectra of highly dispersed VO*_x_* species in this study with crystalline references might be producing large uncertainties in the vanadium speciation, which were not reported. The atomic-scale redox dynamics in vanadium oxide-based catalysts was also visualized using high-resolution transmission electron microscopy under oxidizing (300 °C, 1 mbar O_2_) and reducing (300 °C, vacuum) conditions showing that a VO*_x_* layer on titania reversibly changes its atomic structure, which is consistent with reversible changes in the oxidation state of vanadium.^23^ Several density functional theory (DFT) calculation studies^24−26^ showed that V^3+^ can also be an intermediate involved in the oxidation of alcohols. These simulations, however, were typically performed with only one isolated surface vanadium oxide site. DFT calculations performed on larger clusters involving more than one VO*_x_* species (either neighboring sites or separated by silica) under conditions of propane ODH suggested that the formation of two V^4+^ species is energetically more favorable than the formation of one V^3+^.^27,28^

The rate of oxidation (of alcohols, short alkanes, SO_2_, etc.) over highly dispersed supported vanadia catalysts can be accelerated by several orders of magnitude by varying the oxide support material.^5,8,29−32^ Several hypotheses regarding the role of the oxide support in the ODH of alcohols were discussed in the literature. Wachs and co-workers^33^ proposed that some oxide supports influence the electronic properties of the surface VO*_x_* species that in turn affect the transition state entropy of C–H bond breaking. Bronkema et al.^16^ suggested that the spillover of methoxy species from the support to vanadium oxide may be responsible for the increased activity of VO*_x_*/TiO_2_ during methanol oxidation. DFT calculations found that reducible supports such as ceria^34,35^ and titania^36^ can accept electrons on the f-orbital (for cerium) or delocalize electrons in the subsurface layers (for titanium), thus, facilitating the formation of oxygen vacancies without a necessary reduction of VO*_x_* species. Goodrow and Bell^37^ proposed, based on DFT calculations, that the formation of oxygen vacancies in the support in close proximity to surface VO*_x_* species promotes the methanol ODH by reducing the activation energy for the C–H bond-breaking step. Thus, the ability of the support to form oxygen vacancies correlates with the catalytic activity. The DFT calculations of Yun et al.^38^ and Beck et al.^8^ led to similar conclusions. Yun et al.^38^ also experimentally confirmed the formation of oxygen vacancies by measuring oxygen uptake by a VO*_x_*/TiO_2_ catalyst pre-reduced in ethanol at 200 °C. For ceria-supported vanadia, the formation of Ce^3+^ during ODH of ethanol was shown by *operando* wavelength-selective Raman spectroscopy.^39^ For supported VO*_x_*/TiO_2_, experimental evidence for Ti^3+^ formation was found with EPR during ODH of alkanes.^40,41^ The identification of Ti^3+^ by EPR in the presence of V^4+^, however, is questionable since both signals appear in the same region of 3000–3700 G (see (36) and references therein). On the contrary, ambient pressure XPS experiments^9,15,17^ could not detect the formation of Ti^3+^ during oxidation of methanol and ethanol over supported VO*_x_*/TiO_2_, which suggests that the redox activity of titanium, if any, is not strong in the ODH of alcohols.

In that context, hard X-ray spectroscopy methods are well suited to probe the redox activity of supported metals in catalysts using *operando* approach. XAS experiments can be performed without pressure or material gaps and the detection of products can be performed simultaneously using cells mimicking plug-flow reactors. XAS is also element-specific, thus, allowing to probe the redox activity of each metal selectively as well as quantitatively.^42−44^ Additionally, recent developments in time-resolved XAS combined with non-steady-state experimental strategies have demonstrated that it is possible to detect short-lived reaction intermediates, measure the rates of their formation and decay, and correlate the formation/decay rates of short-lived intermediates with the kinetics of the overall catalytic processes.^45−49^

In the present study, we applied time-resolved XAS methods to probe the mechanism of alcohol oxidation over supported VO*_x_* species promoted by titania. The aim was to clarify whether the redox dynamics of a specific redox process involving potential cation intermediates (V^4+^, V^3+^, or Ti^3+^) quantitatively correlates with the overall catalytic rate. However, probing the vanadium state in VO*_x_* surface species supported on bulk TiO_2_ catalysts by time-resolved XAS is particularly challenging because detection of the V K-edge XAS in the presence of titanium is very inefficient. This is due to the strong absorption of incident X-rays by titanium having its K-edge at 4966 eV that is just below the V K-edge at 5465 eV. Moreover, the V K_α_ line (4945–4953 eV) used for the fluorescence detection of XAS overlaps with the Ti K_β_ line (4933 eV). These problems were minimized by designing bilayered supported VO*_x_*/TiO*_x_*/SiO_2_ catalysts where the surface VO*_x_* species are anchored to surface TiO*_x_* species that are in turn anchored to the SiO_2_ support.^10^ Such model catalysts employ only small amounts of titania and have been shown to be effective catalysts for alcohol ODH. These materials have several advantages for mechanistic studies. First, their activity normalized to vanadium content is comparable to the state-of-the-art VO*_x_*/TiO_2_. Second, they contain less titanium, thus, probing of V K-edge XAS is easier. Third, a large fraction of titanium atoms in these catalysts is close to the surface and, thus, in direct contact with surface vanadium oxide species. Accordingly, the activity of surface titanium atoms in such model catalysts should be easier to detect by the bulk-sensitive Ti K-edge XAS. Thus, we focused on the bilayered supported VO*_x_*/TiO*_x_*/SiO_2_ catalyst consisting of VO*_x_* submonolayer anchored on a TiO*_x_* monolayer supported on SiO_2_ and the process of ethanol ODH. A series of dedicated oxygen cutoff experiments (cycling between an ethanol/oxygen gas mixture and one consisting of ethanol only) in an *operando* reactor were performed to probe the activity of VO*_x_* species in this catalyst. Using multivariate curve resolution methods to analyze the time-resolved V K-edge XANES dataset, we obtained the time-resolved profiles of the V^5+^, V^4+^, and V^3+^ species. The V K-edge XANES pre-edge and edge features of these intermediates were compared to an extensive database of standards and specially tailored molecular references to confirm the oxidation states of these species and to get additional details about their geometric structure. Correlation of the formation and decay rates of V^5+^, V^4+^, and V^3+^ during transient experiments to the rate of acetaldehyde production identified which redox process is kinetically coupled to the rate-determining step of this catalytic process. To probe the subtle redox activity of titanium, we also performed a series of modulation excitation XAS experiments at the Ti K-edge under relevant conditions.

## Materials and Methods

### Preparation of Catalysts

A series of supported bilayered VO*_x_*/TiO*_x_*/SiO_2_ catalysts were synthesized following a well-established method.^10^ The names of the catalysts are expressed in terms of nominal V_2_O_5_ and TiO_2_ loading. None of the catalysts contained crystalline V_2_O_5_; this term was only used to describe the samples’ stoichiometry. The surface TiO*_x_* layer was first deposited on silica support followed by the deposition of the surface vanadia layer. The stoichiometry was varied from 1 to 50 wt % TiO_2_. The VO*_x_* content was always maintained at 5 wt % V_2_O_5_ stoichiometry. For comparison, supported 8 wt % V_2_O_5_/SiO_2_ and 5 wt % V_2_O_5_/TiO_2_ were also synthesized. The surface coverage of VO*_x_* in all catalysts was varied in the interval of 1–6.9 V/nm^2^, which is below monolayer coverage (8 V/nm^2^), Table 1.^50^

**Table 1:** Specific Surface Area and Surface VO*_x_* Density of Catalysts of This Study

catalysts’ names containing nominal metal loading	BET surface area of support before VO*_x_* deposition, m^2^/g^51^	final catalyst BET surface area, m^2^/g	actual V loading in V_2_O_5_ equivalents^a^, wt %^10^	actual Ti loading in TiO_2_ equivalents^a^, wt %^51^	Ti/V atom. ratio	surface density, V/nm^2 ^a	surface density, Ti/nm^2^
8% V_2_O_5_/SiO_2_	332	231	0	0		2.3	0
5% V_2_O_5_/1% TiO_2_/SiO_2_	305	263		1.05	0.23	1.3	0.3
5% V_2_O_5_/5% TiO_2_/SiO_2_	280	174^10^	3.80	6.58	1.1	1.9	1.8
5% V_2_O_5_/8% TiO_2_/SiO_2_	253	211^10^	4.74	9.38	1.8	1.6	2.9
5% V_2_O_5_/15% TiO_2_/SiO_2_	229	189^10^	4.51	15.71	3.4	1.8	4.9
5% V_2_O_5_/25% TiO_2_/SiO_2_		189^10^			5.7	1.8	n/a
5% V_2_O_5_/40% TiO_2_/SiO_2_		175			9.1	1.9	n/a
5% V_2_O_5_/50% TiO_2_/SiO_2_		142			11.4	2.3	n/a
5% V_2_O_5_/TiO_2_	50	48			21.6	6.9	n/a

a Actual concentration determined by inductively coupled plasma (ICP) analysis, taken from refs (10, 51). n/a (not applicable) is indicated for samples with titania content higher than one monolayer.

Catalyst preparation and characterization are described and discussed in detail elsewhere.^10^ In brief, the used silica support was Cabosil EH-5 (Cabot Corporation) and the titania support was P-25 (Degussa). For ease of handling, silica was first treated with water, dried at 120 °C, and calcined overnight at 500 °C. The BET surface area of the resulting SiO_2_ was 332 m^2^/g. The deposition of TiO*_x_* and VO*_x_* overlayers was performed in a glovebox with continuous N_2_ flow. The supported TiO_2_/SiO_2_ materials were prepared by incipient-wetness impregnation of an isopropanol solution of titanium isopropoxide (Ti(O-*i*-C_3_H_7_)_4_, Alfa-Aesar, 99.999% purity). After impregnation, the catalysts were dried in the glovebox overnight and then in N_2_ flow at 120 °C for 1 h and at 300 °C for 1 h. The supported VO*_x_*/TiO*_x_*/SiO_2_, VO*_x_*/SiO_2_, and VO*_x_*/TiO_2_ catalysts were prepared by incipient-wetness impregnation of an isopropanol solution of vanadium(V) oxytriisopropoxide (VO(O-*i*-C_3_H_7_)_3_, Alfa-Aesar 97% purity) on the corresponding TiO*_x_*/SiO_2_, SiO_2_, and TiO_2_ supports. The resulting catalysts were kept in a glovebox overnight and subsequently dried at 120 °C for 3 h and at 300 °C for 1 h in N_2_ flow prior to calcination in air. The supported VO*_x_*/TiO*_x_*/SiO_2_ catalysts were calcined for 1 h at 300 °C and 2 h at 500 °C, whereas the supported VO*_x_*/SiO_2_ and VO*_x_*/TiO_2_ catalysts were calcined for 1 h at 300 °C and 2 h at 450 °C.

### XAS Experiments

*Operando* V K-edge and Ti K-edge XAS were measured on the SuperXAS beamline, at the Swiss Light Source, Villigen, Switzerland operating at 400 mA and 2.4 GeV. The polychromatic beam coming from a 2.9 T superbend magnet was vertically collimated by a Si-coated mirror at 2.5 mrad and subsequently monochromatized by the Si(111) channel-cut monochromator. A Rh-coated toroidal mirror was used to focus the incident X-ray beam to 500 × 400 μm^2^ at the sample position. XAS spectra were recorded in fluorescence mode^52^ using a PIPS diode (Mirion Technologies) as a fluorescence detector. The Si(111) channel-cut monochromator was oscillating with a frequency of 1 Hz, which corresponds to a repetition rate of 2 scans/s. Prior to each data acquisition of the sample in the *operando* cell, the X-ray energy was calibrated by measuring vanadium (for V K-edge at 5465 eV) or titanium metallic foil (for Ti K-edge at 4966 eV) in transmission mode by moving the *operando* cell temporarily out of the beam. Intensities of the incident and transmitted beam were measured with 15 cm long ionization chambers filled with 500 mbar N_2_ and 500 mbar He. All reference samples were measured in transmission mode.

### *Operando* Reaction Cell and Gas Switching Setup

**Figure 1:**
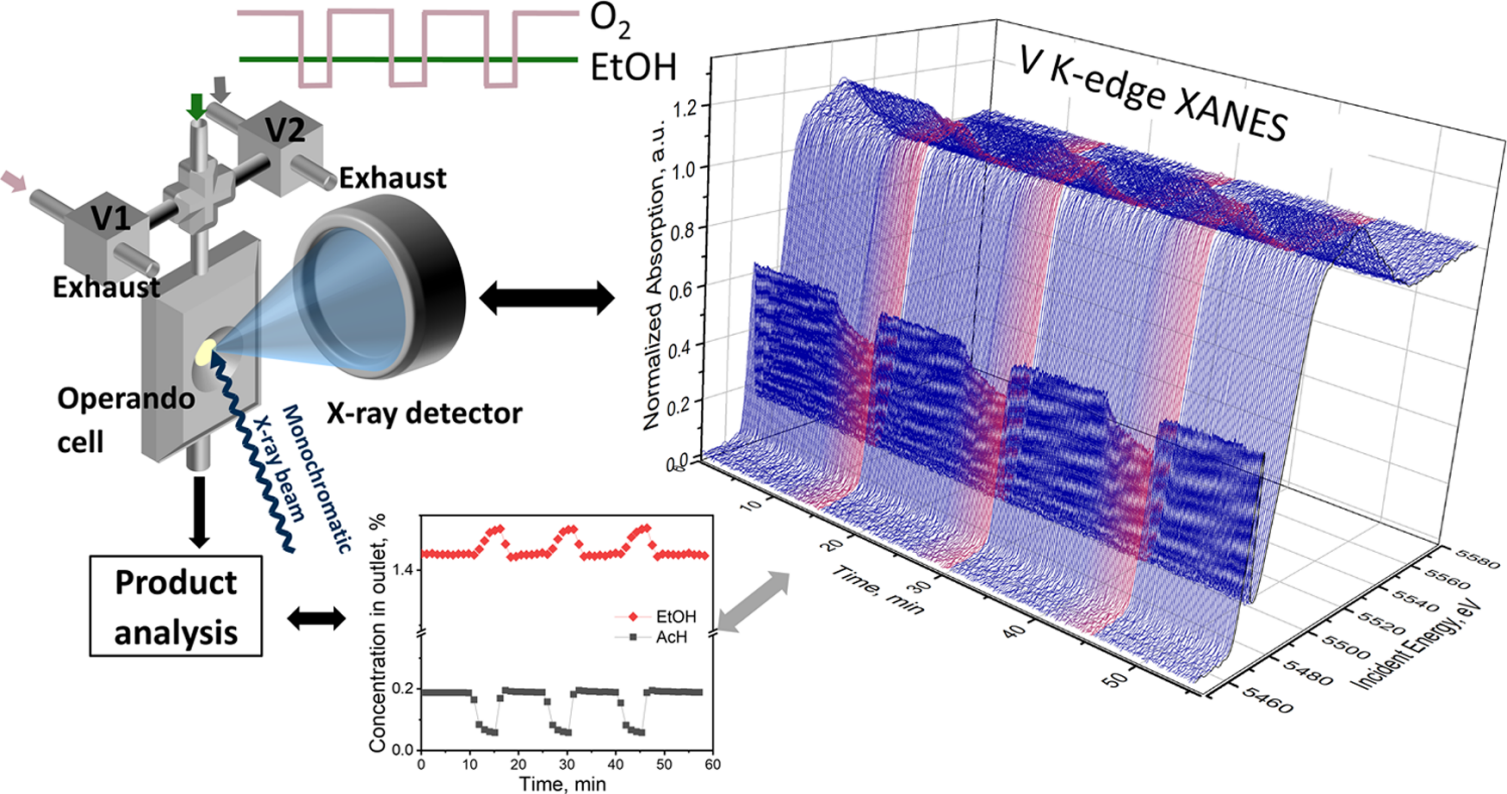
Scheme of the *operando* XAS setup. V1 and V2 are solenoid three-way valves. Product analysis was done using a Bruker Alpha II FT-IR spectrometer. The V K-edge XAS spectra of VO*_x_* species in 5% V_2_O_5_/15% TiO_2_/SiO_2_ catalyst represent the data quality obtained after averaging 20 scans corresponding to 10 s time resolution.

The scheme of the experimental setup for *operando* XAS is shown in Figure 1. All experiments (including experiments performed outside the beamline) were performed in a stainless steel plug-flow reactor cell^53^ equipped with graphite windows (thickness 0.25 mm, Fisher Scientific), which are partially transparent for X-rays and are also used to seal the reactor. A sample inside the cell was heated with two heating cartridges (Moesch). The temperature was controlled in the middle of the reactor using a thermocouple. The products at the reactor outlet were analyzed using a gas phase IR spectrometer (Alpha Bruker).

To supply gas flows to the cell, we used 40% O_2_ in He (4.7) and He (6.0) gas bottles connected to the mass flow controllers (Bronkhorst). The helium gas line was equipped with a moisture trap (Agilent technologies) and an oxygen trap (Restek). The oxygen gas line was equipped with a moisture trap (Agilent technologies). A well-defined concentration of ethanol in a flow of helium (30 mL/min) was achieved using a saturator filled with pure ethanol and placed inside a chiller (Huber, KISS K6, temperature stability ±0.05°), which was typically set to 8 °C. Oxygen cutoff experiments were performed by flowing ethanol-saturated helium constantly through the cell, while identical flows (20 mL/min) of helium and an oxygen-containing helium gas mixture were alternated by means of two three-way switching solenoid valves (Parker, Series 9) (V1 and V2, Figure 1). While one gas mixture went into the reactor, the other flew through a bypass to the exhaust. With this setup, one can perform experiments involving fast changes in the oxygen concentration, while keeping the ethanol concentration (1.6 vol %) and total flow through the cell (50 mL/min) constant. Changing the composition of the gases flowing through the gas lines also allows performing other types of transient experiments, such as the alternation of ethanol and oxygen flows. The *operando* cell allows for fast gas exchange. After switching from one gas to the other, 95% of gas exchange is reached in 2.4 s at 50 mL/min flow (Figure S1a). All gas lines were heated up to 130 °C to avoid condensation of liquids.

For the catalytic tests, we loaded 15–20 mg of a catalyst (63–150 μm fraction) in the cell between two quartz wool plugs. The catalysts were tested without any catalyst dilution. Before each experiment, the samples were pretreated *in situ* by heating to 400 °C in an oxygen-containing flow (20 vol % O_2_ in He, 50 mL/min) at a rate of 12 °C/min and dwelling for 1 h. Afterward, the catalysts were cooled down to the operating temperature. Before performing the transient experiments, steady-state operation conditions were applied by supplying a constant flow consisting of 1.6 vol % of ethanol and 6.4 vol % of oxygen in He for 30 min at 180 °C.

### Transient V K-Edge XAS Experiments and Data Analysis

A series of oxygen cutoff experiments were performed at the V K-edge to track the reduction kinetics of the surface VO*_x_* sites. Initially, the catalyst was exposed to an ethanol–oxygen flow (1.6 vol % EtOH, 6.4 vol % O_2_ in He); after 10 min, the oxygen-containing feed was replaced by an oxygen-free ethanol-containing feed (1.6 vol % EtOH in He). This phase lasted 5 min; afterward, the oxygen was switched on again. At each investigated temperature, we performed 3 oxygen cutoff cycles (10 min in EtOH + O_2_ and 5 min in EtOH) while measuring V K-edge XAS. The reaction conditions (temperature, feed composition, and flow rate) were selected in a way that the experiment could be carried out within low conversion (the highest ethanol conversion was 26%). This ensured the uniformity of the catalyst structure along the bed.

A temperature-programmed reduction (TPR) by ethanol (1.6 vol % EtOH in He 6.0, total flow 50 mL/min) was performed in the temperature interval of 100–400 °C with a heating rate of 5 °C/min. Prior to the ethanol TPR experiment, a standard pretreatment in an oxygen-containing atmosphere was conducted.

The raw V K-edge XAS data were processed using the in-house developed ProQEXAFS software.^54^ The recorded XAS spectra were averaged by every six scans, background-subtracted, normalized to an edge jump of one, and further averaged by averaging spectra at similar time periods within each of the three periods of gas switching to further improve the data quality. For the background subtraction in the pre-edge interval, we used a linear function fit in the interval of −60.0 to −6.1 eV (relative to *E*_0_ = 5465.0 eV). A linear function in the interval of 54.6–136.7 eV was applied for the normalization in the post-edge region. Figure S2 shows the quality of 1 scan and after averaging 6 scans and 18 scans (3 periods). Accordingly, during oxygen cutoff experiments at each temperature, we measured 5400 spectra (3 cycles of 15 min with 2 scans/s), which after processing ended up in one averaged gas switching cycle consisting of 300 spectra with a time resolution of 3 s. For the analysis of the ethanol TPR data, the resulting XAS spectra were averaged by 20 scans and processed as described above. All normalized and averaged XANES spectra (one combined dataset containing the TPR data and the switching experiments at different temperatures) were analyzed together in the energy interval of 5450–5580 eV using multivariate curve resolution alternating least square (MCR-ALS) analysis implemented in MatLab (MCR-ALS GUI2.0,^55^ details are in SI Section 1.7). In the MCR-ALS routine, three constraints were implemented: non-negativity of spectra, non-negativity of concentrations, and the sum of all concentrations equal to 1.

The MCR method is based on solving eq 1
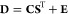
1where **D** (*m* × *n*) is a matrix of raw data containing *m* experimental spectra; **C** (*m* × *k*) is a matrix containing the concentration variation for *k* pure components; **S** (*n* × *k*) contains in columns the corresponding spectra of the pure components; and **E** (*m* × *n*) is the residual and represents an unexplained signal, ideally the experimental uncertainties. Equation 1 can be solved iteratively using the least square optimization (alternating least square, ALS). The solution of eq 1 results in optimization of matrixes **C** and **S**, which will minimize the residual **E**. In other words, MCR allows determining individual spectra (**S**) of components, in a way that the experimental spectra (**D**) could be presented as a sum of these components with a certain contribution (**C**). Ideally, each component corresponds to one specific state of the analyzed atom. However, it is not always possible to distinguish all different states, for example when two states evolve similarly over time. MCR does not require any references.

### Ti K-Edge XAS Experiments

The Ti K-edge XAS spectra were averaged over 2 s, background-subtracted, and normalized to the edge jump of one. In the pre-edge region, the background subtraction was performed using a linear function in the interval of −61.0 to −8.7 eV (relative to *E*_0_ = 4966.0 eV). For normalization in the post-edge region, a cubic polynomial function in the 100.0–492.6 eV interval was used. For the details on the performing and analysis of modulation excitation Ti K-edge XAS refer to SI Section 1.4.

### XAS Measurements of V and Ti Reference Compounds

V-containing references were either purchased from a manufacturer or synthesized in the laboratory. The details can be found in SI Section 1.8.

The sample powders (except the liquid vanadium(V) oxytriisopropoxide) were homogeneously mixed with cellulose or boronitride and pressed into pellets. The pellets containing air-sensitive references (e.g., vanadium(III) oxides and vanadium(IV) oxides) were prepared in a glovebox and sealed in aluminized plastic bags (polyaniline (14 μm), polyethylene (15 μm), aluminum (12 μm), polyethylene (75 μm)). The XAS spectra of all of the powder reference compounds were measured in transmission mode simultaneously with vanadium or titanium foil, which was used for energy calibration. Vanadium(V) oxytriisopropoxide was sealed in a quartz capillary (diameter 0.3 mm, wall thickness 0.01 mm) in a glovebox and measured in fluorescence mode. The measured reference spectra were background-subtracted and normalized in the same way as reported above for the XAS spectra of the catalysts. The absence of X-ray damage was confirmed by the absence of any ongoing changes in the spectra during the measurements with a 2 scan/s repetition rate.

### V K-Edge XANES Pre-Edge and Edge Analysis

For the V K-edge XANES, we quantified the area under the pre-edge peak, the pre-edge intensity, the position of the center of mass (centroid) of the pre-edge and the half-edge step position (the energy at 0.5 au for normalized absorption). The pre-peak height was measured without background subtraction. For more accurate integration of the pre-edge area, a cumulative distribution function (CDF) was used to fit the background of the rising edge with the use of the least square method implemented in Python (Figures S4–S6) and then subtracted. To obtain the pre-edge area, the resulting peak was integrated using the trapezoidal rule (details in SI Section 1.9). The centroid position corresponded to the center of mass of the peak.

### Online Analysis of Products

For offline activity tests of the catalysts, we used a gas chromatograph (MicroGC-MS, SRA) equipped with a stabilWAX column and thermal conductivity detector. For the *operando* spectroscopy investigations, where the time resolution is crucial, the gases at the reactor outlet were analyzed with an online Bruker Alpha II FT-IR Spectrometer, equipped with a 70 mm pathlength cuvette (95% of gas exchange in the cuvette is reached in 15 s, Figure S1b). Each spectrum was recorded for 1 min (47 scans) in the absorbance mode in the interval of 500–4000 cm^–1^ with 4 cm^–1^ resolution. Prior to every experiment (after standard pretreatment in O_2_ or He flow), the background IR spectrum was recorded. The bands of acetaldehyde (1853–1658 cm^–1^) and ethanol (1294–1181 cm^–1^) not overlapping with other signals were extracted and analyzed using the MCR-ALS approach^55^ (details are in SI Section 1.10).

## Results and Discussion

### Catalytic Activity for Ethanol ODH

**Figure 2:**
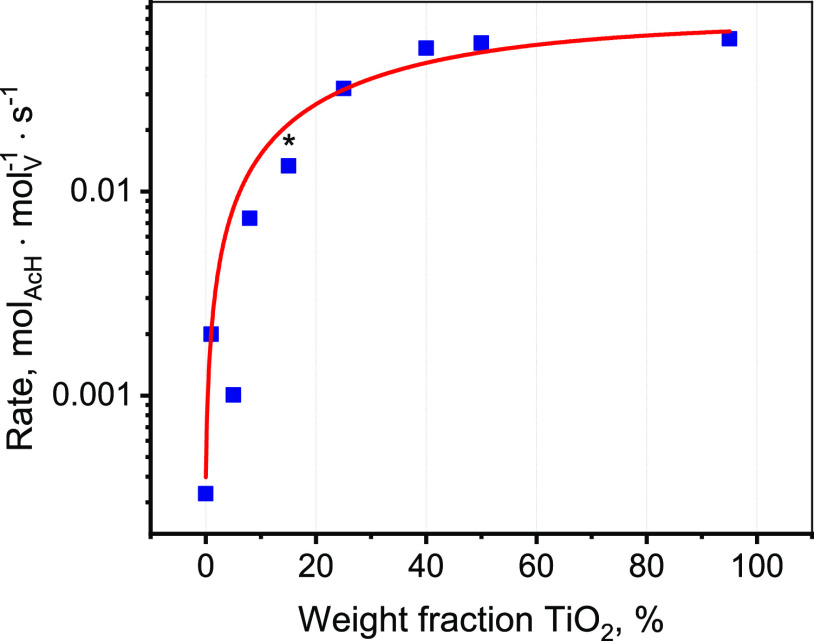
Reaction rates for ethanol ODH to acetaldehyde per V over the supported VO*_x_* species in 8% V_2_O_5_/SiO_2_ (*x* = 0), 5% V_2_O_5_/TiO_2_ (*x* = 95), and 5% V_2_O_5_/*x*% TiO_2_/SiO_2_ catalysts as a function of TiO_2_ loading (*x*) at 200 °C (feed 1.6 vol % EtOH, 6.4 vol % O_2_ in He). More details about catalytic activity are summarized in Table S1. The catalyst marked with an asterisk (*) was chosen for the *operando* XAS investigations.

The activity of the supported VO*_x_*/TiO*_x_*/SiO_2_, VO*_x_*/TiO_2_, and VO*_x_*/SiO_2_ catalysts for selective oxidation of ethanol to acetaldehyde as a function of titanium oxide loading is presented in Figure 2. The data were collected at 200 °C and normalized to the vanadium loading. The concentrations of vanadium oxide corresponded to a surface density of 1.3–2.3 V/nm^2^ for VO*_x_*/TiO*_x_*/SiO_2_ and VO*_x_*/SiO_2_ catalysts and 6.9 V/nm^2^ for the VO*_x_*/TiO_2_ catalyst, which is below the value for monolayer coverage (8 V/nm^2^).^50^ The concentrations of titania (*x*) deposited on the SiO_2_ support in the VO*_x_*/TiO*_x_*/SiO_2_ catalysts was varied in the range of 1–50% TiO_2_. Raman spectroscopy revealed that all of the catalysts contain exclusively surface VO*_x_* species (V = O vibrations at ∼1030 cm^–1^) and no crystalline V_2_O_5_ nanoparticles were detected (characteristic Raman vibration at 994 cm^–1^, Figure S8).^10^ The selectivity toward acetaldehyde for all catalysts was above 90% and several nonselective products (acetic acid, CO_2_, and ethylene) were also detected.

The acetaldehyde production rate progressively increases with titania loading as shown in Figure 2. The major gain in catalytic activity, by a factor of 40, is observed between 0 and 15% of TiO_2_. Further addition of titania causes only a 4 times increase in activity. The surface density of titania on silica for the 5% V_2_O_5_/15% TiO_2_/SiO_2_ catalyst corresponds to 4.9 Ti/nm^2^, which is close to the estimated monolayer coverage (4 Ti/nm^2^ ^51^ and 5.5 Ti/nm^2^ ^29^). *In situ* DR UV–vis spectroscopy of the calcined 5% V_2_O_5_/15% TiO_2_/SiO_2_ catalyst revealed that the catalyst contains mostly amorphous surface TiO*_x_* species and only around 0–5% crystalline TiO_2_ (details are in SI Section 4.1).

The catalytic results indicate that approximately monolayer coverage of surface TiO*_x_* on SiO_2_ is enough for the effective promotion of surface VO*_x_* species anchored at the surface TiO*_x_* species. A similar activity trend was reported for 1% V_2_O_5_/*x*% TiO_2_/SiO_2_ catalysts in methanol ODH with a saturation of catalytic activity observed at ca. 12% TiO_2_ loading.^10^*In situ* Raman spectroscopy detected the bands of V–O–V (465, 606 cm^–1^), V–O–Ti (800, 916 cm^–1^), and Ti–O–Si (1070 cm^–1^) as well as a band at 1034 cm^–1^, assigned to the stretching V=O anchored to the surface TiO*_x_* species. The presence of surface VO*_x_* species in 5% V_2_O_5_/15% TiO_2_/SiO_2_ catalyst only anchored to the SiO_2_ support was tentatively ruled out since a vanadyl stretch for such silica-supported VO*_x_* domains (1041 cm^–1^) was not detected (Figure S27b). *In situ* DR UV–vis showed that this catalyst contains mostly oligomeric (assigned to dimeric and trimeric) surface VO*_x_* species (details are in SI Section 4.1). In comparison, supported VO*_x_* species in the VO*_x_*/SiO_2_ catalysts are mostly present in the isolated (monomer) state.^56^

Based on the ethanol oxidation catalytic activity, the supported 5% V_2_O_5_/15% TiO_2_/SiO_2_ catalyst was chosen for the mechanistic studies using *operando* XAS. This catalyst demonstrated high selectivity toward acetaldehyde (close to 100%) in a wide temperature range (160–210 °C) and near-zero order of reaction in both reactants (0.2 in ethanol and 0.1 in oxygen, Figure S9). These reaction orders suggest that under reaction conditions the catalyst surface is extensively covered with adsorbed ethanol and that the catalyst reoxidation step is faster than the reduction step. The obtained reaction orders are close to the near-zero orders reported in the literature for ethanol ODH over alumina-supported vanadia catalysts.^12,14^ The apparent activation energy measured for the 5% V_2_O_5_/15% TiO_2_/SiO_2_ catalyst is 68 kJ/mol, which is close to 66 kJ/mol observed for the 5% V_2_O_5_/TiO_2_ and 66–67 kJ/mol, reported in the literature for VO*_x_*/TiO_2_ catalysts.^8,38^ In comparison, the apparent activation energy for 8% V_2_O_5_/SiO_2_ containing no TiO*_x_* layer is higher at 86 kJ/mol (Table S1).

### Redox Activity of Vanadium during Ethanol Oxidation

To understand which surface VO*_x_* species are involved in the catalytic cycle of selective oxidation of alcohols, we performed a series of transient *operando* V K-edge XAS experiments with the bilayered supported 5% V_2_O_5_/15% TiO_2_/SiO_2_ catalyst. The main transient experiments were oxygen cutoff experiments and consisted of periodical oxygen removal from the ethanol–oxygen gas feed. Such experiments were performed at six temperatures in the range of 160–210 °C. Upon oxygen cutoff, the reoxidation step of the catalytic cycle (reaction 2 in Scheme 1) was interrupted, which allowed the detection of short-lived reduced intermediates. In addition, we performed an *operando* experiment during TPR in ethanol in the temperature range of 100–400 °C. The averaged and normalized V K-edge XAS spectra were processed by multivariate curve resolution alternating least square (MCR-ALS) analysis.^55,57,58^ For our system, the MCR-ALS approach is better suited than linear combination fitting using known references since the latter requires spectra of references, which are typically crystalline and therefore not well suited for fitting the spectra of completely dispersed surface vanadium oxide species.

**Figure 3:**
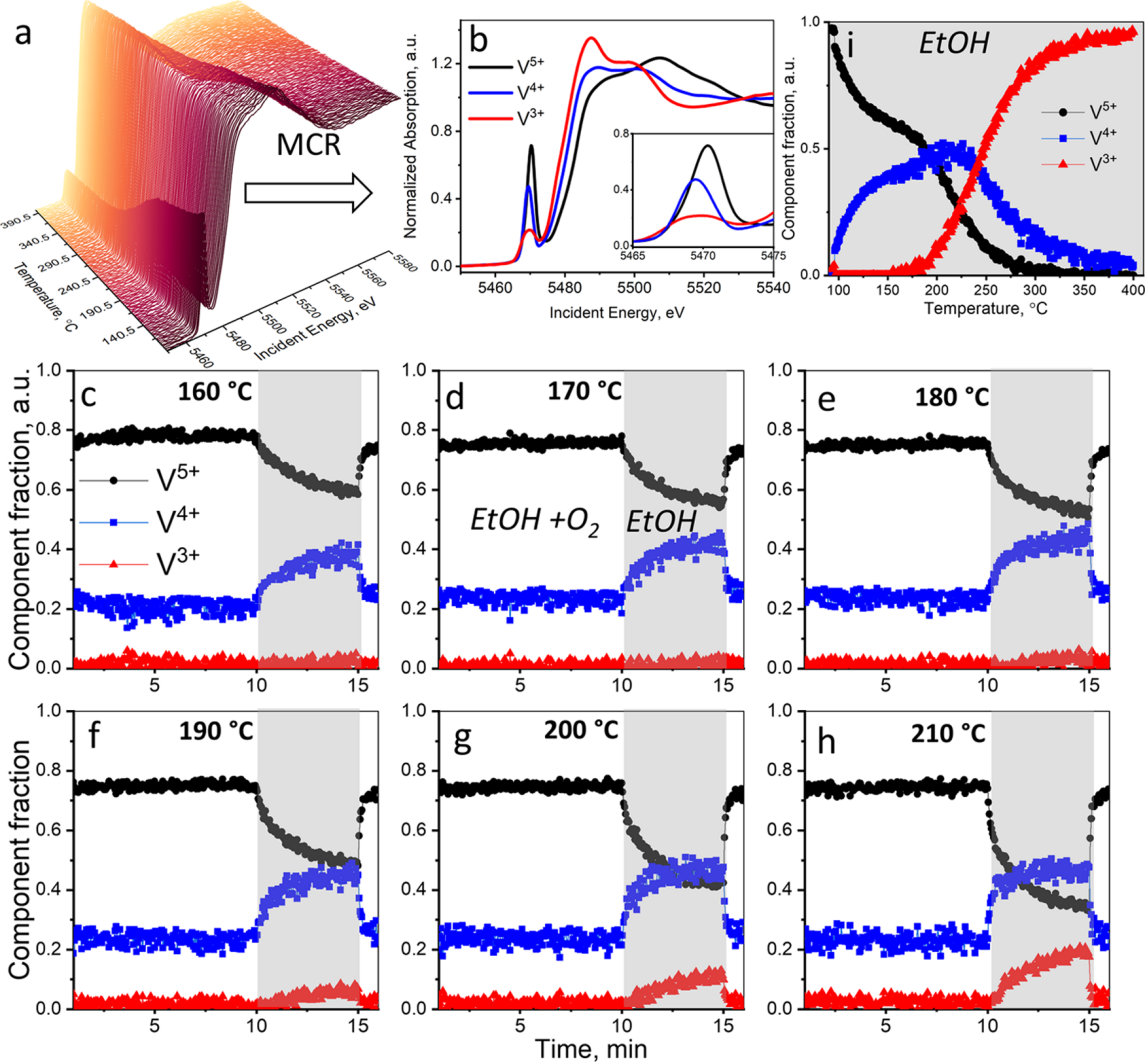
(a) V K-edge spectra of VO*_x_* species measured *operando* in ethanol TPR using the 5% V_2_O_5_/15% TiO_2_/SiO_2_ catalyst. (b) V K-edge XAS components resolved by MCR-ALS from *operando* experiments using the supported 5% V_2_O_5_/15% TiO_2_/SiO_2_ catalyst. The inset shows the pre-edge region. (c–h) Concentration profiles of the V^5+^, V^4+^, and V^3+^ components during oxygen cutoff experiments at 160, 170, 180, 190, 200, and 210 °C, respectively. (i) Concentration profiles of the V^5+^, V^4+^, and V^3+^ components during ethanol TPR. In (c–i), the white background corresponds to the 1.6 vol % EtOH and 6.4 vol % O_2_ feed; the gray background corresponds to the 1.6 vol % EtOH only feed.

The optimal number of components for MCR-ALS was determined using the lack-of-fit analysis and was equal to three (details in SI Section 4.3). The spectra of the components and their concentration profiles were resolved using the MCR-ALS approach during oxygen cutoff experiments performed at different temperatures and ethanol TPR experiments (shown in Figure 3). The resolved XAS components are tentatively assigned to V^5+^, V^4+^, and V^3+^, and more careful assignments by comparison to spectra of reference compounds will be discussed below. Importantly, the V^4+^ component cannot be represented as a linear combination of the V^5+^ and V^3+^ spectra. This is clearly visible in the pre-edge region shown in the inset of Figure 3b: at 5468 eV the V^4+^ component has the highest absorption value and, thus, it cannot be obtained by superposition of the V^3+^ and V^5+^ spectra.

### Comparison of MCR-ALS Resolved Components to Vanadium Reference XAS Spectra

It is known that the shape and intensity of the V K-edge XAS pre-edge and edge features strongly depend on the oxidation state and the local structure of vanadium. The pre-edge peak appears due to the electron transition from 1s to 3d levels of vanadium, which is a dipole forbidden transition. However, this transition becomes partially allowed in noncentrosymmetric local structures of vanadium due to polarization of p-orbitals and 3d–4p orbital mixing that leads to an increase in the pre-edge peak intensity.^59−61^ Moreover, the extent of p–d mixing depends on several factors, such as covalency, oxidation state, and exact geometry (length of bonds and symmetry) of vanadium.^62^ The pre-edge height, the pre-edge area, the pre-edge position, the edge position, and their different combinations are often used as fingerprints to estimate the oxidation state and coordination number of vanadium atoms surrounded by oxygen. Wong et al.^59^ showed that the intensity of the pre-edge peak (half-width multiplied by the height of the pre-edge) correlates with the vanadium coordination and average V–O bond length, whereas the edge position is correlating with the oxidation state (d*^n^* electron configuration). Giuli et al.^60^ demonstrated that the pre-edge peak height and the pre-edge center of mass position (centroid) can be used for the identification of the vanadium oxidation state. This approach was also used by Sutton et al.^63^ Chaurand et al.^64^ have concluded that the area under the pre-edge peak and the pre-edge centroid position are the most reliable characteristics for the determination of vanadium oxidation state and symmetry. The edge position as a single or main descriptor is also often used to determine the vanadium oxidation state.^65−68^ Alternatively, Silversmit et al.^69^ have used the difference between the edge position (half-edge jump) and the pre-edge position (the position of the maximum intensity) to determine the vanadium oxidation state.

**Figure 4:**
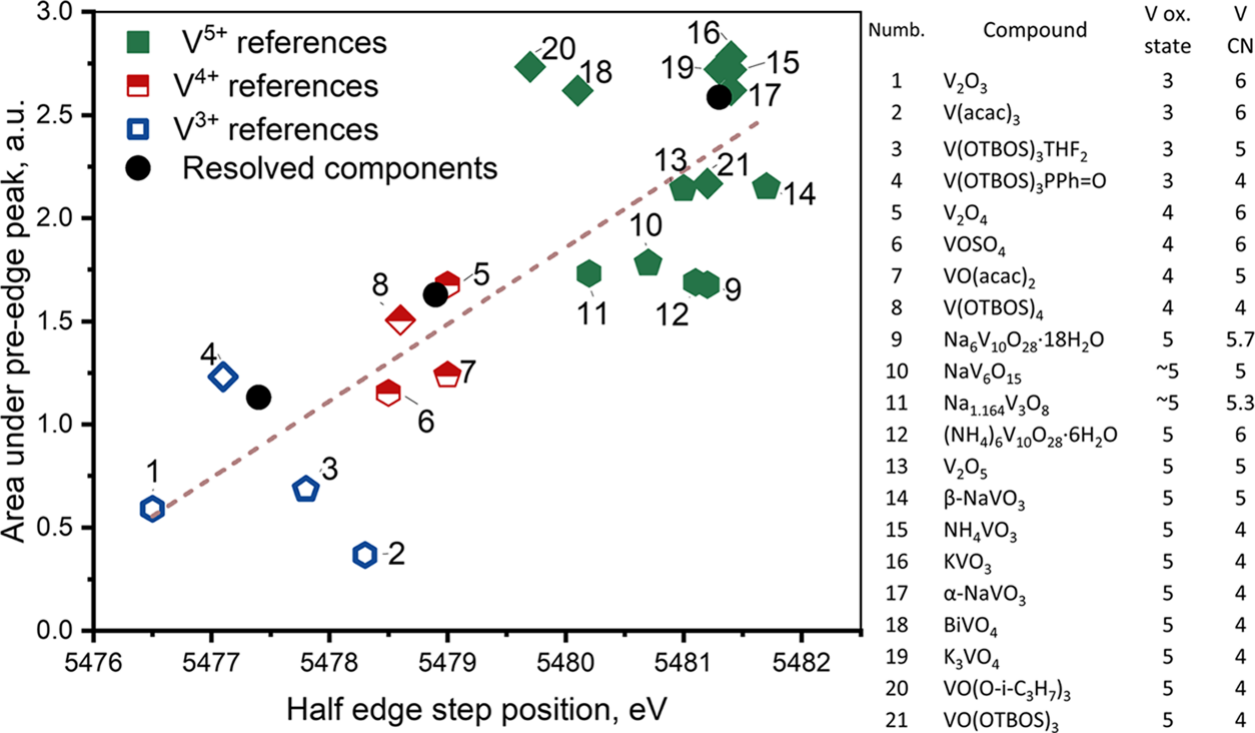
Area under the V K-pre-edge peak and the half-edge step position for the reference compounds (listed on the right with their oxidation states (V ox. state) and coordination numbers (CN)) and the MCR-ALS resolved components for VO*_x_* species in the 5% V_2_O_5_/15% TiO_2_/SiO_2_ catalyst. Acac: acetylacetonate; OTBOS: OSi(O-*tert*-C_4_H_9_)_3_; Ph: C_6_H_5_. Different shapes of symbols represent different coordination numbers of vanadium: ⧫—4-fold-coordinated; ⬟—5-fold-coordinated; ⬢—6-fold-coordinated.

To verify the oxidation states of the MCR-ALS resolved VO*_x_* components corresponding to surface intermediates in the catalyst, we have measured V K-edge XANES of 21 commercially available standards and tailored reference compounds containing vanadium coordinated by oxygen in different oxidation states and local geometries (Figure S10). For all of these structures, we quantified the area under the pre-edge peak, the pre-edge height, the pre-edge center of mass (centroid position), and the position of the half-edge step (*E*_1/2_). Analysis of these descriptors (details in SI Section 1.9) showed that the area under the pre-edge peak and the energy of the half-edge jump are the most reliable parameters to assign the oxidation state of the VO*_x_* components. A correlation plot comparing these descriptors for all of the references and the MCR-ALS resolved components is shown in Figure 4. With increasing vanadium oxidation state, both the half-edge step position and the pre-edge peak area increase. Based on Figure 4, our assignments of the oxidation states of the MCR-ALS resolved XAS spectra to V^5+^, V^4+^, and V^3+^ components are, thus, confirmed. The MCR resolved XANES components represent the average signatures of V^5+^, V^4+^, and V^3+^ states of all VO*_x_* species present in the catalyst, which can have slightly different local structures. Nevertheless, V K-edge XANES is very sensitive to the oxidation state and less sensitive to the local coordination of vanadium; therefore, the use of the MCR-XANES method for the identification of the oxidation state of VO*_x_* species is appropriate.

Additionally, the area of the pre-edge peak helped us to obtain a better insight into the local geometry of MCR-ALS resolved components. For a fixed oxidation state, a larger pre-edge area is typically observed for noncentrosymmetric VO_4_ in tetrahedral coordination and progressively decreases for VO_5_ in square pyramidal and for VO_6_ in distorted octahedral coordination. This trend is clear for V^5+^ references. Based on this correlation, we can conclude that V^5+^ species in the catalyst are predominantly VO_4_ (tetrahedral) with a possible small fraction of VO_5_ (square pyramidal). Commercially available V^3+^ references (V_2_O_3_ and V(acac)_3_) contain vanadium in octahedral environment. The surface V^3+^ species of the catalyst are, however, unlikely to be 6-fold-coordinated. For the better assignment of the resolved V^3+^ component, we synthesized and fully characterized original molecular references containing V^3+^ in tetrahedral (V(OTOBOS)_3_PPhO) or trigonal-bipyramidal (V(OTOBOS)_3_thf_2_) environments (Figures S11, S29, and S30). A similar approach was successfully used to understand the structure of surface chromium species of the Phillips catalyst.^70^ The pre-edge intensity of the synthesized molecular V^3+^ references (especially of 4-coordinated V(OTOBOS)_3_PPhO) is significantly higher in comparison to 6-coordinated V_2_O_3_ and V(acac)_3_ references and much better correlates to the value observed for V^3+^ surface species. Concerning the V^4+^ component, its geometry cannot be reliably assigned based on Figure 4. This is due to the lack of low-coordinated reference compounds and the limitation of our descriptor approach. However, based on the previous assignment of the local geometry of V^5+^ and V^3+^ components and chemical intuition, we can hypothesize that the V^4+^ intermediate should be also rather low-coordinated and contain no more than four oxygen neighbors.

### Involvement of Vanadium in the Mechanism of Alcohol Oxidation

The changes of the V^5+^, V^4+^, and V^3+^ concentration (molar fraction) during the oxygen cutoff experiments are shown in Figure 3c–h. In the first 10 min of the experiments, the catalyst was exposed to the ethanol–oxygen mixture, selective ethanol reduction was taking place and the catalytic conversion was analyzed by online IR-spectroscopy (Figure S12). Under these conditions, 75–80% of all VO*_x_* species in the catalyst are in the V^5+^ state and 20–25% are in the V^4+^ state. As suggested in the literature,^8,9,12^ the step of V^4+/3+^ reoxidation (Scheme 1, reaction 2) by molecular oxygen should be much faster than the step of V^5+^ reduction by ethanol (Scheme 1, reaction 1), which explains why in the presence of ethanol and oxygen the majority of vanadium species are present in the highest oxidation state of +5.

After 10 min in the reaction mixture (Figure 3c–h), oxygen is removed from the feed, and over the next 5 min, the catalyst is exposed to the pure ethanol feed (1.6 vol % EtOH in He). Removing oxygen from the feed eliminates the vanadium reoxidation step (reaction 2 in Scheme 1), which leads to the accumulation of reduced vanadium intermediates. Thus, in Figure 3c–h, we see a decrease in the fraction of V^5+^ with a simultaneous increase in the concentration of V^4+^. The V^3+^ species is not detected at 160 and 170 °C. Starting from 180 °C, however, the formation of V^3+^ could also be observed. After 15 min, the oxygen supply is switched back on and the concentrations of all vanadium species and the activity of the catalyst are rapidly restored to the initial values (Figures 3 and S12). This clearly evidences the complete reversibility of the vanadium reduction during oxygen cutoff experiments.

**Figure 5:**
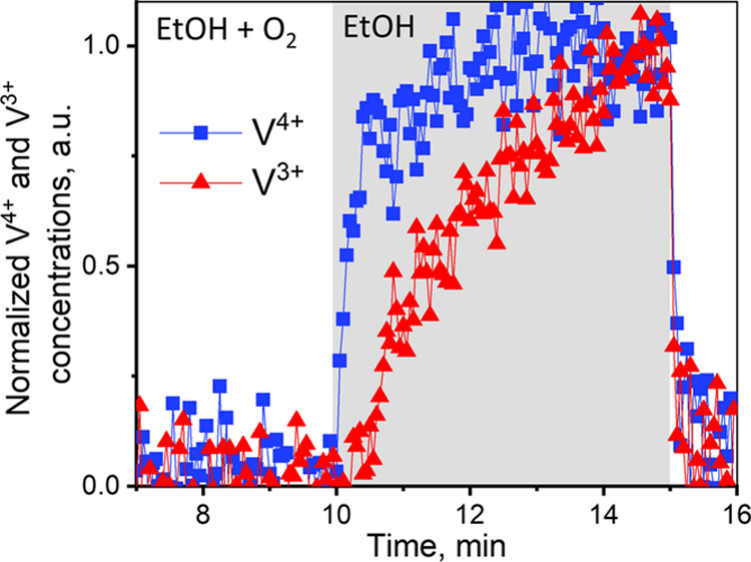
Relative changes in V^4+^ and V^3+^ concentrations during the oxygen cutoff experiment at 210 °C for VO*_x_* species in the 5% V_2_O_5_/15% TiO_2_/SiO_2_ catalyst.

To clarify whether V^4+^ and V^3+^ are formed simultaneously during exposure to ethanol or whether V^3+^ is formed in a secondary process (irrelevant to catalysis), we compared the evolution of normalized V^4+^ and V^3+^ concentrations at the highest temperature of 210 °C (Figure 5). Note that V^3+^ appears with a 20–25 s delay after the V^4+^ fraction almost reaches its maximum. At lower temperatures (Figure S13), the delay is less pronounced but still present. This clearly demonstrates that V^4+^ is the main reduced vanadium species, which may be formed under steady-state operating conditions.

The concentration profiles of the *operando* XAS experiments with the VO*_x_* species in the 5% V_2_O_5_/15% TiO_2_/SiO_2_ catalyst during ethanol TPR are shown in Figure 3i. The V^4+^ species appear already at 100 °C, whereas V^3+^ only begins to be formed at ca. 190 °C, which correlates with the oxygen cutoff experiments. The analysis of reaction products during ethanol TPR revealed additional products: ethane and ethylene (Figure S14). The formation of ethane starts only at ca. 260 °C and is accompanied by an increase in the concentration of acetaldehyde. Acetaldehyde and ethane can be formed over reduced V^3+^ species by ethanol disproportionation as previously described in the literature.^71,72^ The formation of ethylene is observed starting from ca. 250 °C and is presumably formed over acidic sites (such as V–OH, Ti–OH, Si–OH). Importantly, at the temperatures of oxygen cutoff experiments (160–210 °C), no other products apart from acetaldehyde were detected both in the presence and in the absence of oxygen.

To demonstrate that changes in the evolution of the VO*_x_* species during oxygen cutoff experiments are not affected by exposure to intense X-rays,^73^ we conducted several additional tests. We performed oxygen cutoff experiments with differently focused beam sizes at 160 °C. We have chosen the lowest temperate since all processes related to catalysis are slower at these conditions, which facilitates the detection of the photoreduction processes. The data were analyzed using a linear combination fitting approach using the MCR-ALS resolved V^5+^, V^4+^, and V^3+^ components. Only V^4+^ and V^5+^ were found in these experiments, whereas V^3+^ was not detected (Figure 3c) even at the brightest (most focused) X-ray beam. The evolution of the V^5+^ component in all experiments is shown in Figure S15a. The concentration profiles vary on the level of noise and do not demonstrate a correlation with the beam size. To identify whether the exposure to X-rays may have an influence on the vanadium oxidation state under steady-state conditions in the ethanol–oxygen mixture, we also performed a so-called beam switching experiment. The catalyst was exposed to the ethanol–oxygen feed, while the V K-edge XAS was recorded simultaneously. After 10 min, the beam was completely switched off for the next 10 min and then switched on again (Figure S15b). XAS acquisition was never stopped. If the X-ray beam influences the vanadium speciation, the vanadium species would change their structure immediately after switching the beam back on. As the state of vanadium was not changing and was apparently identical in the presence and the absence of the X-ray beam, we concluded that the vanadium speciation in our experiments is not affected by the X-ray beam. In addition, the V K-edge XANES spectra of the fresh catalyst and the catalyst after ca. 24 h of operation (under X-ray beam during steady-state and transient XAS experiments) (Figure S16) are also identical (under dehydrating conditions). It evidences that VO*_x_* species did not undergo significant structural changes during *operando* XAS experiments.

### Kinetics of Vanadium Reduction and Reoxidation in the MvK Cycle of Ethanol ODH

**Figure 6:**
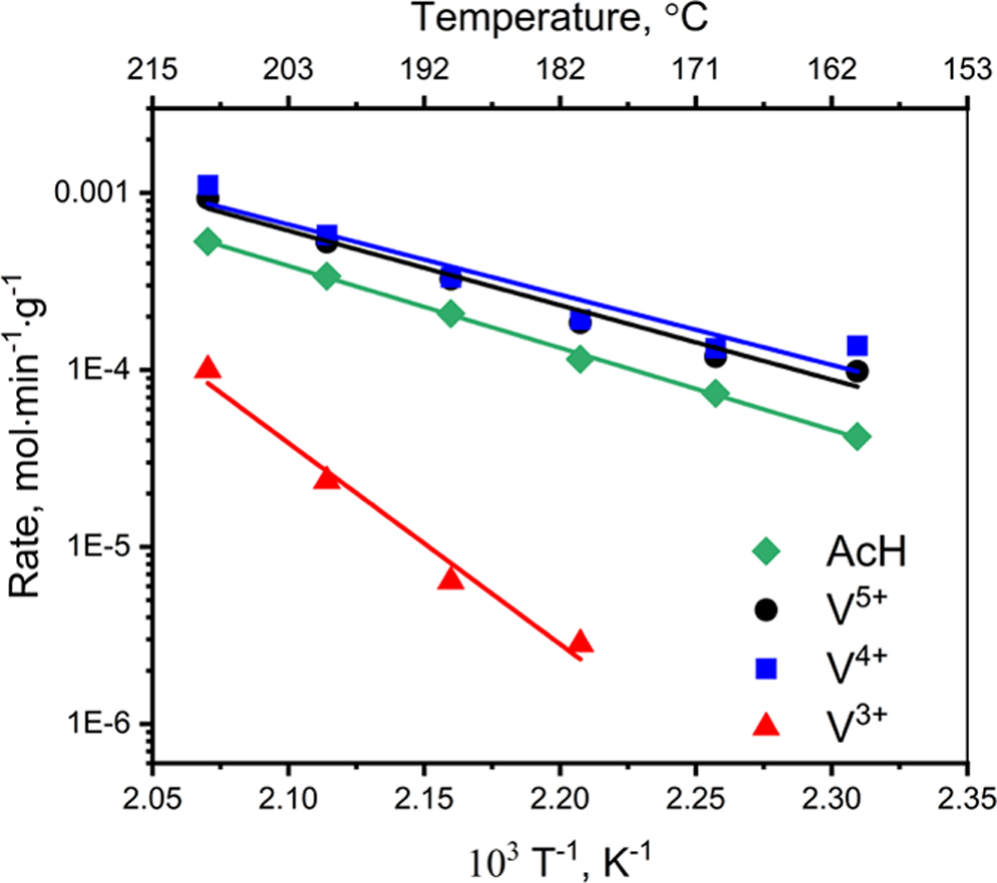
Background-corrected rates of acetaldehyde production (in the ethanol–oxygen mixture) and the initial rates of V^5+^ consumption and V^4+^ and V^3+^ formation after switching to ethanol, estimated from time-resolved V K-edge XAS for VO*_x_* species in the 5% V_2_O_5_/15% TiO_2_/SiO_2_ catalyst. For V^5+^ and V^4+^, the corresponding initial rates were calculated starting at *t*_0_ = 10.0 min (the moment when oxygen was switched off); for V^3+^, *t*_0_ was 10.4 min, due to the delay in the appearance of the V^3+^ signal.

If ethanol ODH proceeds via the MvK mechanism (Scheme 1), the rates of V^4+^ or V^3+^ or Ti^3+^ formation should correlate with the rate of acetaldehyde production. We evaluated the initial rates of V^4+^ and V^3+^ formation and V^5+^ consumption after switching off oxygen and correlated them to the catalytic rates. We fitted the V^5+^, V^4+^, and V^3+^ concentration profiles shown in Figure 3c–h in the interval of 1–15 min (Figures S16 and S17). Before oxygen cutoff (1 min < *t* < *t*_0_), we used a constant function to fit the concentration profile; after oxygen cutoff (*t*_0_ < *t* < 15 min), we applied either linear or exponential decay functions (details are in SI Section 1.11). For V^4+^ and V^5+^ profiles, *t*_0_ was set equal to 10 min, the moment of switching off the oxygen. As V^3+^ appeared with a delay, the *t*_0_ was set at 10.4 min. To estimate the initial rates of V^4+^/V^3+^ formation and V^5+^ decay after oxygen switching off, we determined the first derivative of the resulting fits at time *t*_0_. Figure 6 shows the initial vanadium transformation rates in ethanol compared to the rate of acetaldehyde production before oxygen switching off. The acetaldehyde production never decreased to zero in the absence of oxygen in the feed since the graphite windows used for sealing of the cell were not completely leak-tight (Figure S19). The residual (background) activity in ethanol was around 1 × 10^–6^ mol_AcH_·min^–1^ and was almost independent of the catalyst loading and reaction temperature. The rates of acetaldehyde production in Figure 6 were corrected by subtracting the above-mentioned background value (0.5–1 × 10^–4^ mol·min^–1^·g^–1^). This subtraction removed the activity of a fraction of the vanadium sites that performs the catalytic cycle both in the ethanol–oxygen and the ethanol feed containing oxygen impurity and, therefore, does not change oxidation state during the oxygen cutoff experiments (e.g., at 210 °C the background activity is ca. 10% of the total activity).

Ethanol oxidation to acetaldehyde involves the transfer of two electrons. If V^4+^ is the main redox intermediate, the expected rate of V^4+^ formation should be 2 times higher than the rate of acetaldehyde production. According to Figure 6, the rates of V^4+^ formation and V^5+^ consumption are similar and about 1.5–1.8 times higher than the acetaldehyde production rate over the entire temperature range. This reasonably matches the electron balance of acetaldehyde formation, considering the complexity of chemical speciation by time-resolved V K-edge XAS. To the best of our knowledge, no other technique could correlate the rates of intermediate formation and product production with similar precision. These results suggest that V^4+^ formation is kinetically coupled to the acetaldehyde production over VO*_x_* species promoted by a TiO*_x_* monolayer. In contrast, the formation of V^3+^ is delayed and is 10–70 times lower than the production of acetaldehyde. This is a clear indication that V^3+^ is not the main intermediate involved in this catalytic process.

According to the literature, the V^4+/3+^ reoxidation step by molecular oxygen (Scheme 1, Step 2) is considered to be much faster than V^5+^ reduction by alcohol.^8,9,12,16^ However, to the best of our knowledge, there have been no quantitative experiments that could estimate the rate of this process in alcohol ODH. We attempted to estimate the rates of V^4+^ reoxidation upon oxygen introduction in oxygen cutoff experiments (Figure 3c–h), similar to what we did for V^5+^ reduction (details and fits are given in SI Sections 1.12 and 4.6). We observed a significant increase in the V^4+^ reoxidation rates as a function of temperature, indicating that the kinetics is not limited by the exchange of gas in the cell. Reoxidation of V^4+^ is clearly faster than V^5+^ reduction and, therefore, cannot be the rate-determining step, our estimation shows that reoxidation of V^4+^ is ca. 6–8 times faster than the rate of V^5+^ reduction in the investigated temperature range (Table S5). This explains the presence of V^4+^ species in the ethanol–oxygen mixture at 160–210 °C (Figure 3c–h). Based on the ratio between the oxidation and reduction rates, under steady-state conditions, 6–12% of all vanadium species in the catalyst should be statistically present in the V^4+^ state (Table S5, details in SI Section 4.6). Experimentally we observed 20–25% of V^4+^ in the ethanol–oxygen flow (Figure 3c–h). The higher experimental values can be due to the presence of additional V^4+^ not involved in the catalytic cycle^74^ or to the oversimplification of our model.

### Titanium State in Calcined Catalyst

**Figure 7:**
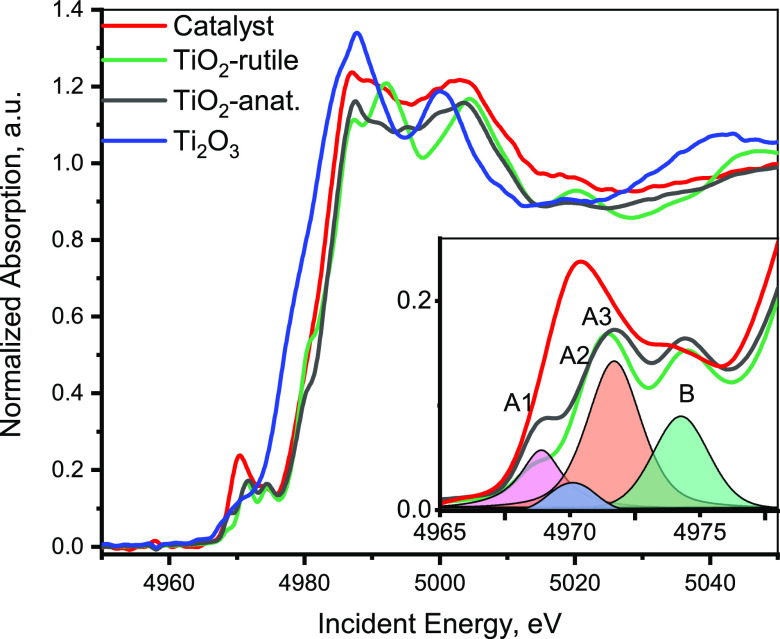
Ti K-edge XANES of the TiO_2_-anatase, TiO_2_-rutile, Ti_2_O_3_, and TiO*_x_* species in the 5% V_2_O_5_/15% TiO_2_/SiO_2_ catalyst (measured at 350 °C in 6 vol % O_2_). The inset amplifies the pre-edge region. The A1, A2, A3, and B components were fitted for the anatase spectrum.

Figure 7 shows the Ti K-edge XAS spectrum of the TiO*_x_* species in the bilayered supported 5% V_2_O_5_/15% TiO_2_/SiO_2_ catalyst (with close to one monolayer surface TiO*_x_* coverage) measured at 350 °C in an oxygen-containing atmosphere (6.4 vol % O_2_) together with TiO_2_-rutile, TiO_2_-anatase, and Ti_2_O_3_ references. The Ti K-edge position for the catalyst is close to that of the TiO_2_ references indicating that the oxidation state of titanium in the catalyst is close to +4. The overall shape of the pre-edge and the post-edge features of the catalyst, however, significantly differ from those of the bulk titania references, which is due to the high dispersion of TiO*_x_* and its interaction with both the surface VO*_x_* species and silica sites of the SiO_2_ support. The pre-edge feature of Ti K-edge XANES originates from the 1s–3d transition and appears due to the polarization of p-orbitals and strong 3d–4p orbital mixing.^75^ Farges et al.^76^ have shown that the pre-edge intensity increases with a decrease in the coordination number of titanium. Thus, a small pre-edge intensity is characteristic of 6-fold-coordinated compounds (e.g., TiO_2_-rutile and TiO_2_-anatase), whereas 5-fold-coordinated and, especially, 4-fold-coordinated compounds in tetrahedral coordination demonstrate an intense pre-edge peak with a height close to the normalized XAS edge step.

The pre-edge peaks of anatase and rutile consist of four components typically labeled in the literature as A1, A2, A3, and B (Figure 7).^75,77,78^ It was suggested that A1 and A2 have mostly a local character and vary upon structural changes in the first coordination shell of titanium, whereas A3 and B correspond to nonlocal transitions.^75^ The A2 peak appears due to lattice defects, e.g., distortion around Ti atoms or changes in the coordination number. Investigations of the Ti K-edge XANES of TiO_2_ nanoparticles showed that A2 increases with a decrease in particle size and positively correlates with the nanoparticle surface-area-to-volume ratio.^78,79^ In addition, it was found that the A2 peak intensity could be diminished by the adsorption of oxygen-donating ligands (e.g., ascorbic acid) on the nanoparticle surface.^80^ The Ti K-edge XAS spectrum of TiO*_x_* species in the 5% V_2_O_5_/15% TiO_2_/SiO_2_ catalyst under dry conditions (350 °C in O_2_) shown in Figure 7 exhibits a rather high A2 component and resembles the XANES spectra of amorphous or nanoparticle titania.^77,78,81^ The Ti K pre-edge peak of the catalyst also resembles the simulated pre-edge peak for the central atom in an anatase-like supercell (of 768 atoms) having one missing oxygen in the equatorial position modeled by Rossi et al.^75^ Thus, we assume that the structure of Ti^4+^ in our catalyst under dry conditions is distorted octahedral and most probably partially 5-fold-coordinated, which is in agreement with the *in situ* DR UV–vis observations (SI Section 4.1).

### Activity of Titanium during Alcohol Oxidation

To probe the redox activity of titanium during ODH of ethanol, we initially planned to perform similar *operando* oxygen cutoff experiments at the Ti K-edge as we did for the V K-edge. However, we discovered that in spite of the fact that the TiO*_x_* species in the 5% V_2_O_5_/15% TiO_2_/SiO_2_ catalyst are in the surface bilayer, the redox activity of titanium is extremely weak. For this reason, we performed a series of modulation excitation (ME) XAS experiments at the Ti K-edge, which are much more sensitive to tiny spectral changes. This approach is based on a periodic perturbation of a catalytic system, for example, by changing the composition of the gas feed. The resulting time-resolved spectra can be transformed into phase-resolved spectra by applying the phase-sensitive detection (PSD) procedure (details in the Materials and Methods section). The phase-sensitive analysis reduces the level of noise allowing the detection of small but reproducible changes in the spectra.^82^

**Figure 8:**
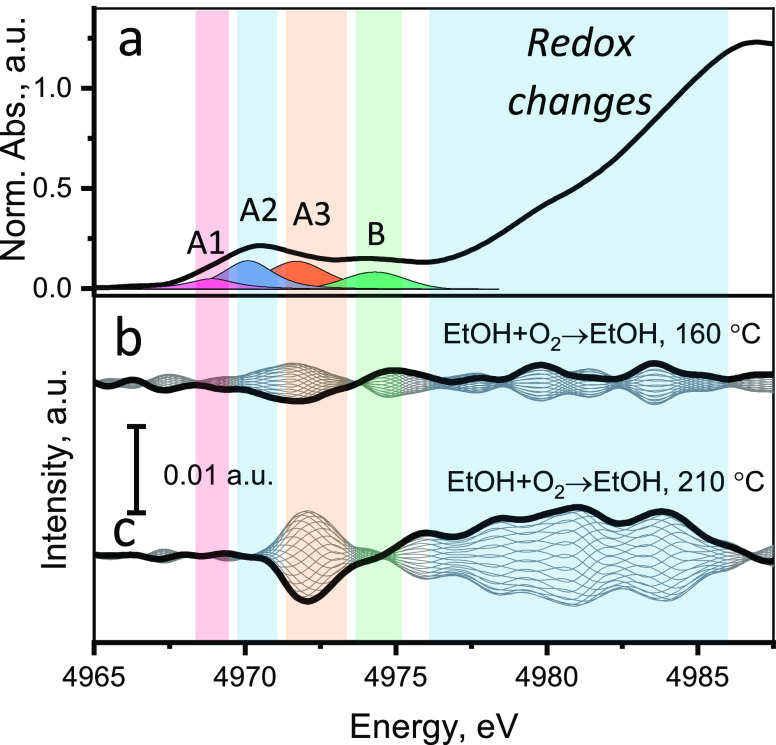
(a) Ti K-edge XANES spectrum (20 vol % O_2_, 400 °C) of TiO*_x_* species in the 5% V_2_O_5_/15% TiO_2_/SiO_2_ catalyst. (b, c) Phase-resolved spectra from the ME Ti K-edge XAS experiments during the periodic switching off oxygen from the ethanol–oxygen feed at 160 and 210 °C, respectively. Bold curves in (b, c) represent spectral changes when switching from more oxidizing (EtOH + O_2_) to more reducing (EtOH) feed. The colored intervals highlight the expected changes in the A1, A2, A3, and B features in the pre-edge region (4968–4975 eV) and in the edge region (4977–4986 eV).

In the *operando* ME Ti K-edge XAS experiments, the ethanol concentration in the feed was held constant, and the flow of oxygen was periodically switched on and off every 5 min (denoted as EtOH + O_2_ → EtOH cycling). Additionally, we performed a second type of the ME Ti K-edge XAS experiments (discussed in detail in SI Section 4.7), in which the oxygen and ethanol flows were periodically (every 5 min) alternated. After recording 10 cycles at each working temperature, the resulting Ti K-edge XAS spectra were normalized and analyzed using the PSD approach in the XANES region. The phase-resolved spectra of TiO*_x_* species from the ME XAS experiments over the supported 5% V_2_O_5_/15% TiO_2_/SiO_2_ catalyst are shown in Figure 8 together with the Ti K-edge XANES spectrum of the same catalyst measured at 350 °C in an oxygen flow.

If titanium undergoes a redox cycle in the catalytic ethanol ODH cycle, Ti^3+^ species would be formed in the absence of oxygen and could be readily detected by a change in the Ti K-edge position. For instance, the reference compound containing Ti^3+^ in octahedral coordination (Ti_2_O_3_) demonstrates an edge shift of ca. 3 eV toward lower energies in comparison with Ti^4+^ in TiO_2_ (rutile, anatase) (Figure 7). For the phase-resolved Ti K-edge XANES spectra, changes in the edge region (around 4977–4986 eV) are also observed (Figure 8). These changes are minor at 160 °C but more obvious at 210 and 350 °C (see Figure S37). These data strongly indicate reversible changes in the titanium oxidation state of a very small fraction of the Ti atoms during the catalytic cycle.

**Figure 9:**
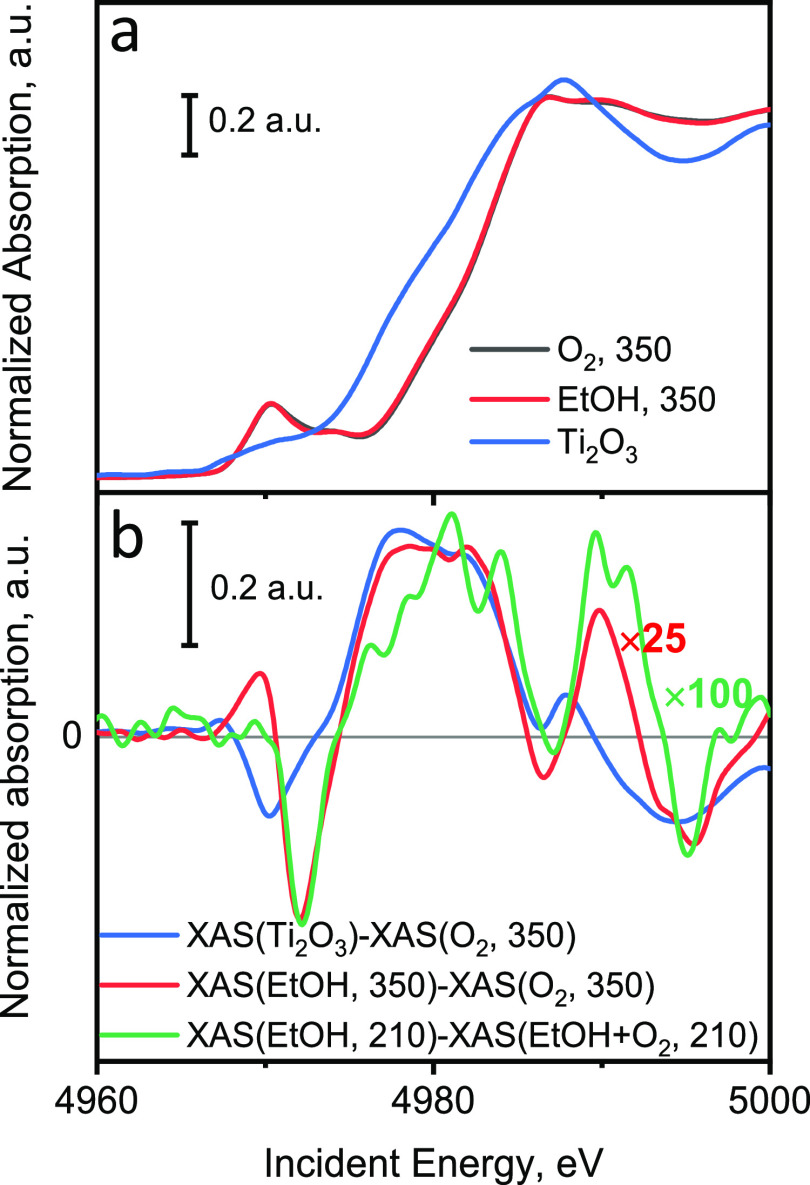
(a) Ti K-edge XANES spectra of TiO*_x_* species in the supported 5% V_2_O_5_/15% TiO_2_/SiO_2_ catalyst measured in O_2_ and EtOH at 350 °C in comparison with the corresponding spectrum of Ti_2_O_3_. (b) Differential Ti K-edge XANES spectra: the blue curve is the difference between the spectra of Ti_2_O_3_ and the 5% V_2_O_5_/15% TiO_2_/SiO_2_ catalyst in 6.4 vol % O_2_ in He at 350 °C; the red curve is the difference between the spectra of the 5% V_2_O_5_/15% TiO_2_/SiO_2_ catalyst measured in 1.6 vol % EtOH in He at 350 °C and the same catalyst in 6.4 vol % O_2_ in He at 350 °C; the green curve is the difference between the spectra of the 5% V_2_O_5_/15% TiO_2_/SiO_2_ catalyst measured in 1.6 vol % EtOH in He at 210 °C and the same catalyst in 1.6 vol % EtOH and 6.4 vol % O_2_ in He at 210 °C.

We attempted to roughly estimate the maximal extent of titanium reduction of TiO*_x_* species during these ME XAS experiments. For this, we averaged the Ti K-edge XANES spectra measured under static conditions in various atmospheres (EtOH, O_2_, and EtOH + O_2_, details are in SI Section 1.4) (Figure 9a). The resulting spectra were used to make the differential XAS spectra (Figure 9b). To estimate the changes in titanium oxidation state, we used the reference spectrum which represents 100% titanium reduction from +4 to +3. This spectrum was received as the difference between the Ti K-edge XAS spectra of the TiO*_x_* species in the 5% V_2_O_5_/15% TiO_2_/SiO_2_ catalyst measured at 350 °C in O_2_ (assumed 100% Ti^4+^) and that of Ti_2_O_3_ (100% Ti^3+^) (Figure 9b). The differential spectrum, representing the changes in the state of Ti in the supported 5% V_2_O_5_/15% TiO_2_/SiO_2_ catalyst upon switching between ethanol and oxygen at 350 °C multiplied by a factor of 25 (Figure 9b) resembles the reference spectrum suggesting a 4% reduction of Ti^4+^. At the relevant working temperature of 210 °C, the changes upon oxygen cutoff experiment (switching between ethanol–oxygen mixture and ethanol) are even smaller (Figure 9b). However, the shape of the differential spectrum is similar to the reference spectrum when multiplied by a factor of 100. This indicates that only around 1% of all TiO*_x_* species in the 5% V_2_O_5_/15% TiO_2_/SiO_2_ catalyst changed their oxidation state at 210 °C during ethanol ODH. Taking into account the extent of vanadium reduction under similar conditions, (∼65% at 210 °C in ethanol, Figure 3h), the molar ratio between vanadium and titanium species reduced in ethanol is around 20:1. This estimation indicates that despite the fact that titanium can potentially accept electrons, the main redox intermediate during ODH of ethanol is V^4+^ and that only a minor amount of Ti^3+^ can participate in the redox kinetics.

Comparison of the phase-resolved Ti K-edge spectra in the pre-edge region (4968–4970 eV) (Figure 8) also revealed interesting details. No significant changes in the pre-edge peaks A1 and A2 (which are sensitive to structural changes in the first coordination shell) were detected (Figure 8b,c). This suggests that the coordination number of Ti does not change in the reducing environment. The reversible changes were detected in the pre-edge at the A3 peak position. The simulation of the Ti K pre-edge XANES of TiO_2_-anatase made by Rossi et al.^75^ showed that the A3 peak is not related to the first coordination shell of Ti^4+^ and appeared only if the size of the investigated cluster was larger than 4 Å and included at least the second shell of Ti^4+^ ions. We, therefore, hypothesize that changes observed in the A3 peak intensity could reflect vanadium reduction which takes place in close proximity of the titanium atoms. To clarify this, in Figure S22, we compared the extent of vanadium reduction and the amplitude of the Ti A3 pre-edge peak changes. The plot shows a clear correlation; stronger changes in vanadium oxidation state upon switching gas lead to greater changes in the A3 peak of the Ti K-edge XANES.

### Overall Mechanism

**Scheme 2:**
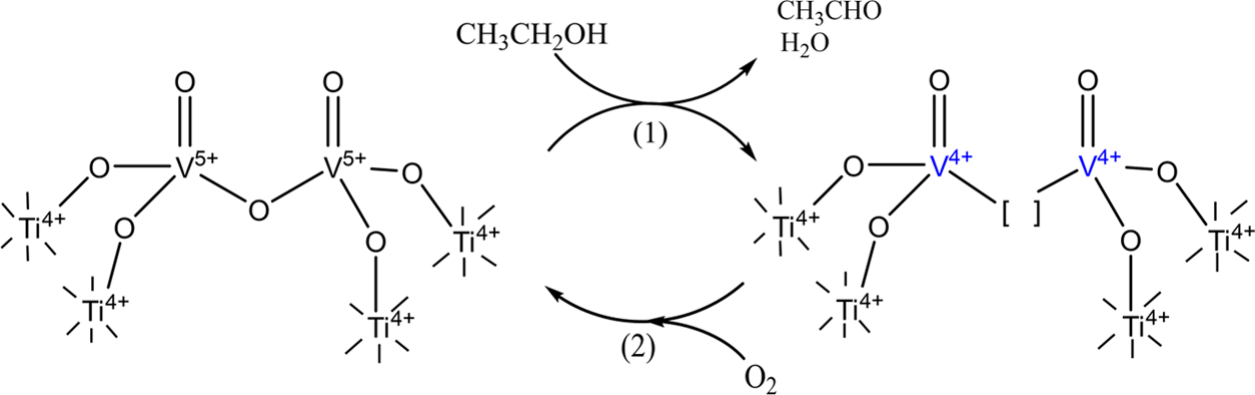
Suggested Redox Mechanism of Ethanol ODH over Supported VO*_x_* Species Supported on a TiO*_x_* Layer The position of the oxygen vacancy is hypothetic.

The catalytic mechanism of ethanol oxidation by VO*_x_* species supported on TiO*_x_* is summarized in Scheme 2. *In situ* DR UV–vis showed that the VO*_x_* species on the catalyst surface are present as dimers and trimers. V K-edge XAS showed that in the absence of oxygen, in an alcohol-containing environment, both reduced species, V^4+^ and V^3+^, are formed. This agrees with the findings reported in the literature. For example, V^4+^ and V^3+^ species were detected at relevant conditions using XPS for VO*_x_*/TiO_2_^9,15,17^ and VO*_x_*/Al_2_O_3_^22^ catalysts. At the same time, by performing transient XAS experiments we were able to quantitatively decipher that the formation of V^4+^ is kinetically coupled to the acetaldehyde production, whereas the appearance of V^3+^ species is delayed and 10–70 times slower. This finding is in agreement with DFT calculations suggesting that the formation of two V^4+^ species is energetically more favorable than the formation of one V^3+^.^27,28^ The comparison of the resolved spectra with the broad reference database indicated that the V^5+^, V^4+^, and V^3+^ species have low-coordinated nature (the coordination number is not greater than four). The extremely weak redox activity of titanium was detected by Ti K-edge XAS. This, from one side proves that titanium can accept electrons during ODH, which is in agreement with DFT predictions;^36^ from another side, it shows, that the redox activity of the TiO*_x_* species is not kinetically coupled to the rate-determining step of ethanol ODH, and, thus, the promotional role of titania could have another origin. For example, the surface TiO*_x_* species can be involved in a fast transport of electrons on the surface or modify the electronic structure of VO*_x_* species, which facilitates their reducibility. ME Ti K-edge XAS showed that upon switching between reducing (EtOH) and oxidizing (EtOH + O_2_) feeds, no changes occur in the local coordination of titanium. This suggests that the oxygen vacancy is not formed in the first coordination sphere of Ti. Thus, the most probable position of the oxygen vacancy is between two vanadium atoms, however, it remains rather hypothetic. The surface VO*_x_* species promoted by a monolayer of TiO*_x_* demonstrate about 40 times higher activity in comparison to VO*_x_* on silica and only 4 times lower than VO*_x_* on bulk TiO_2_. This suggests that the promoting effect of bulk TiO_2_ on the activity of VO*_x_* species is probably not related to titanium reducibility.

## Conclusions

We studied the redox activity of vanadium and titanium during ODH of ethanol over completely dispersed supported surface VO*_x_* species promoted by titania using *operando* time-resolved XAS. To facilitate the XAS investigation at the V and Ti K-edges, we prepared a model bilayered catalyst, 5% V_2_O_5_/15% TiO_2_/SiO_2_, consisting of VO*_x_* species anchored on a TiO*_x_* monolayer supported on SiO_2_. The surface VO*_x_* species in this bilayered catalyst demonstrated high activity, about 40 times higher than the activity of vanadia on silica and only 4 times lower than VO*_x_* on pure titania. Dedicated *operando* oxygen cutoff experiments performed during the ethanol ODH reaction using V K-edge XAS showed that V^4+^ intermediates form much faster in comparison to V^3+^ species. The extensive database of V K-edge XANES of standards and specially synthesized molecular crystals suggested the mainly low-coordinated nature (coordination number 4 or lower) of the V^5+^, V^4+^, and V^3+^ surface species. A quantitative correlation of the initial V^4+^ and V^3+^ formation rates after oxygen cutoff and steady-state acetaldehyde formation rates showed that V^4+^ formation is kinetically coupled to acetaldehyde formation according to the MvK mechanism, while V^3+^ formation is at least 10–70 times slower. Reoxidation of V^4+^ by oxygen is 6–8 times faster than V^5+^ reduction. To track very subtle changes in the surface titanium state during ethanol ODH, we performed a series of modulation excitation Ti K-edge XAS experiments. Semiquantitative estimation showed that the extent of Ti^4+^ reduction in TiO*_x_* species of 5% V_2_O_5_/15% TiO_2_/SiO_2_ catalyst is about 20 times lower than that of V^5+^ reduction under similar conditions. This suggests that the TiO*_x_* redox activity is not kinetically coupled to the rate-determining step of the catalytic cycle. Probably, titanium changes its oxidation state much faster or modifies the electronic structures of VO*_x_* and in these ways facilitates the reduction of V^5+^ into the V^4+^ state.

## Supporting Information Available

Method description and detailed discussion of *in situ* Raman and DR UV–vis spectroscopy, synthesis procedures of the V-containing references and their structure, MCR ALS analysis, analysis of V K-edge XAS of references, determination of the rates of V reduction and oxidation, and ME Ti K-edge XAS (PDF)
Crystallographic data of V(OTBOS)_3_(Ph3PO) (CIF)
Crystallographic data of V{OSi(OMes)(OtBu)_2_}_3_THF_2_ (CIF)

## Supplementary Material

au2c00027_si_001.pdf

au2c00027_si_002.cif

au2c00027_si_003.cif

## Abbreviations

ODH: oxidative dehydrogenation
MvK: Mars–van Krevelen
TPR: temperature-programmed reduction
ME: modulation excitation
MCR ALS: multivariate curve resolution alternating least square
PSD: phase-sensitive detection
acac: acetylacetonate ion
OTBOS: OSi(O-*tert*-C_4_H_9_)_3_

## References

[ref1] Heracleous E.; Machli M.; Lemonidou A. A.; Vasalos I. A. Oxidative Dehydrogenation of Ethane and Propane over Vanadia and Molybdena Supported Catalysts. J. Mol. Catal. A: Chem. 2005, 232, 29–39. 10.1016/j.molcata.2005.01.027.

[ref2] Busca G.; Elmi A. S.; Forzatti P. Mechanism of Selective Methanol Oxidation over Vanadium Oxide-Titanium Oxide Catalysts: A FT-IR and Flow Reactor Study. J. Phys. Chem. A 1987, 91, 5263–5269. 10.1021/j100304a026.

[ref3] Fumagalli C.; Golinelli G.; Mazzoni G.; Messori M.; Stefani G.; Trifirò F. Production of Maleic and Phthalic Anhydrides by Selective Vapor Phase Oxidation with Vanadium Oxide Based Catalysts. Stud. Surf. Sci. Catal. 1994, 82, 221–231. 10.1016/S0167-2991(08)63414-X.

[ref4] Wachs I. E.; Jehng J.-M.; Deo G.; Weckhuysen B. M.; Guliants V. V.; Benziger J. B.; Sundaresan S. Fundamental Studies of Butane Oxidation over Model-Supported Vanadium Oxide Catalysts: Molecular Structure-Reactivity Relationships. J. Catal. 1997, 170, 75–88. 10.1006/jcat.1997.1742.

[ref5] Dunn J. P.; Stenger H. G.; Wachs I. E. Oxidation of Sulfur Dioxide over Supported Vanadia Catalysts: Molecular Structure - Reactivity Relationships and Reaction Kinetics. Catal. Today 1999, 51, 301–318. 10.1016/S0920-5861(99)00052-8.

[ref6] Cavalli P.; Cavani F.; Manenti I.; Trifiro’ F. Ammoxidation of Alkylaromatics over V2O5/TiO2 Catalysts. Catal. Today 1987, 1, 245–255. 10.1016/0920-5861(87)80043-3.

[ref7] Amiridis M. D.; Wachs I. E.; Deo G.; Jehng J. M.; Kim D. S. Reactivity of V2O5 Catalysts for the Selective Catalytic Reduction of NO by NH3: Influence of Vanadia Loading, H2O, and SO2. J. Catal. 1996, 161, 247–253. 10.1006/jcat.1996.0182.

[ref8] Beck B.; Harth M.; Hamilton N. G.; Carrero C.; Uhlrich J. J.; Trunschke A.; Shaikhutdinov S.; Schubert H.; Freund H. J.; Schlögl R.; Sauer J.; Schomäcker R. Partial Oxidation of Ethanol on Vanadia Catalysts on Supporting Oxides with Different Redox Properties Compared to Propane. J. Catal. 2012, 296, 120–131. 10.1016/j.jcat.2012.09.008.

[ref9] Kaichev V. V.; Chesalov Y. A.; Saraev A. A.; Klyushin A. Y.; Knop-Gericke A.; Andrushkevich T. V.; Bukhtiyarov V. I. Redox Mechanism for Selective Oxidation of Ethanol over Monolayer V2O5/TiO2 Catalysts. J. Catal. 2016, 338, 82–93. 10.1016/j.jcat.2016.02.022.

[ref10] Gao X.; Bare S. R.; Fierro J. L. G.; Wachs I. E. Structural Characteristics and Reactivity/Reducibility Properties of Dispersed and Bilayered V _2_ O _5_ /TiO _2_ /SiO _2_ Catalysts. J. Phys. Chem. B 1999, 103, 618–629. 10.1021/jp983357m.

[ref11] Weckhuysen B. M.; Keller D. E. Chemistry, Spectroscopy and the Role of Supported Vanadium Oxides in Heterogeneous Catalysis. Catal. Today 2003, 78, 25–46. 10.1016/S0920-5861(02)00323-1.

[ref12] Kilos B.; Bell A. T.; Iglesia E. Mechanism and Site Requirements for Ethanol Oxidation on Vanadium Oxide Domains. J. Phys. Chem. C 2009, 113, 2830–2836. 10.1021/jp8078056.

[ref13] Oyama S. T.; Zhang W. True and Spectator Intermediates in Catalysis: The Case of Ethanol Oxidation on Molybdenum Oxide as Observed by in Situ Laser Raman Spectroscopy. J. Am. Chem. Soc. 1996, 118, 7173–7177. 10.1021/ja960468v.

[ref14] Nair H.; Gatt J. E.; Miller J. T.; Baertsch C. D. Mechanistic Insights into the Formation of Acetaldehyde and Diethyl Ether from Ethanol over Supported VOx, MoOx, and WOx Catalysts. J. Catal. 2011, 279, 144–154. 10.1016/j.jcat.2011.01.011.

[ref15] Andrushkevich T. V.; Kaichev V. V.; Chesalov Y. A.; Saraev A. A.; Buktiyarov V. I. Selective Oxidation of Ethanol over Vanadia-Based Catalysts: The Influence of Support Material and Reaction Mechanism. Catal. Today 2017, 279, 95–106. 10.1016/j.cattod.2016.04.042.

[ref16] Bronkema J. L.; Leo D. C.; Bell A. T. Mechanistic Studies of Methanol Oxidation to Formaldehyde on Isolated Vanadate Sites Supported on High Surface Area Anatase. J. Phys. Chem. C 2007, 111, 14530–14540. 10.1021/jp073826x.

[ref17] Kaichev V. V.; Popova G. Y.; Chesalov Y. A.; Saraev A. A.; Zemlyanov D. Y.; Beloshapkin S. A.; Knop-Gericke A.; Schlögl R.; Andrushkevich T. V.; Bukhtiyarov V. I. Selective Oxidation of Methanol to Form Dimethoxymethane and Methyl Formate over a Monolayer V2O5/TiO2 Catalyst. J. Catal. 2014, 311, 59–70. 10.1016/j.jcat.2013.10.026.

[ref18] Burcham L. J.; Gao X.; Wachs I. E.; Deo G. In Situ IR, Raman, and UV-Vis DRS Spectroscopy of Supported Vanadium Oxide Catalysts during Methanol Oxidation. Top. Catal. 2000, 11–12, 85–100. 10.1023/A:1027275225668.

[ref19] Vieira L. H.; Possato L. G.; Chaves T. F.; Lee J. J.; Sulmonetti T. P.; Jones C. W.; Martins L. Insights into Redox Dynamics of Vanadium Species Impregnated in Layered Siliceous Zeolitic Structures during Methanol Oxidation Reactions. ChemCatChem 2020, 12, 141–151. 10.1002/cctc.201901567.

[ref20] Waleska P.; Rupp S.; Hess C. Operando Multiwavelength and Time-Resolved Raman Spectroscopy: Structural Dynamics of a Supported Vanadia Catalyst at Work. J. Phys. Chem. C 2018, 122, 3386–3400. 10.1021/acs.jpcc.7b10518.

[ref21] Vining W. C.; Strunk J.; Bell A. T. Investigation of the Structure and Activity of VO x/CeO 2/SiO 2 Catalysts for Methanol Oxidation to Formaldehyde. J. Catal. 2012, 285, 160–167. 10.1016/j.jcat.2011.09.024.

[ref22] Wu W.; Ding K.; Liu J.; Drake T.; Stair P.; Weitz E. Methanol Oxidation to Formate on ALD-Prepared VO x / θ -Al 2 O 3 Catalysts: A Mechanistic Study. J. Phys. Chem. C 2017, 121, 26794–26805. 10.1021/acs.jpcc.7b07498.

[ref23] Ek M.; Ramasse Q. M.; Arnarson L.; Georg Moses P.; Helveg S. Visualizing Atomic-Scale Redox Dynamics in Vanadium Oxide-Based Catalysts. Nat. Commun. 2017, 8, 305 10.1038/s41467-017-00385-y. 28824163 PMC5563508

[ref24] Döbler J.; Pritzsche M.; Sauer J. Oxidation of Methanol to Formaldehyde on Supported Vanadium Oxide Catalysts Compared to Gas Phase Molecules. J. Am. Chem. Soc. 2005, 127, 10861–10868. 10.1021/ja051720e. 16076191

[ref25] Goodrow A.; Bell A. T. A Theoretical Investigation of the Selective Oxidation of Methanol to Formaldehyde on Isolated Vanadate Species Supported on Silica. J. Phys. Chem. C 2007, 111, 14753–14761. 10.1021/jp072627a.

[ref26] González-Navarrete P.; Gracia L.; Calatayud M.; Andres J. Density Functional Theory Study of the Oxidation of Methanol to Formaldehyde on a Hydrated Vanadia Cluster. J. Comput. Chem. 2010, 109, 2493–2501. 10.1002/jcc.21543. 20652991

[ref27] Rozanska X.; Kondratenko E. V.; Sauer J. Oxidative Dehydrogenation of Propane: Differences between N2O and O2 in the Reoxidation of Reduced Vanadia Sites and Consequences for Selectivity. J. Catal. 2008, 256, 84–94. 10.1016/j.jcat.2008.03.002.

[ref28] Rozanska X.; Fortrie R.; Sauer J. Oxidative Dehydrogenation of Propane by Monomeric Vanadium Oxide Sites on Silica Support. J. Phys. Chem. C 2007, 111, 6041–6050. 10.1021/jp071409e.

[ref29] Quaranta N. E.; Soria J.; Cortés Corberán V.; Fierro J. L. G. Selective Oxidation of Ethanol to Acetaldehyde on V2O5/TiO2/SiO2 Catalysts: Effect of TiO2-Coating of the Silica Support. J. Catal. 1997, 171, 1–13. 10.1006/jcat.1997.1760.

[ref30] Roozeboom F.; Cordingley P. D.; Gellings P. J. Vanadium Oxide Monolayer Catalysts. The Vapor-Phase Oxidation of Methanol. J. Catal. 1981, 68, 464–472. 10.1016/0021-9517(81)90116-0.

[ref31] Deo G.; Wachs I. E. Reactivity of Supported Vanadium Oxide Catalysts: The Partial Oxidation of Methanol. J. Catal. 1994, 146, 323–334. 10.1006/jcat.1994.1071.

[ref32] Bañares M.; Martínez-Huerta M. V.; Gao X.; Fierro J. L. G.; Wachs I. E. Dynamic Behavior of Supported Vanadia Catalysts in the Selective Oxidation of Ethane. In Situ Raman, UV-Vis DRS and Reactivity Studies. Catal. Today 2000, 61, 295–301. 10.1016/S0920-5861(00)00388-6.

[ref33] Wachs I. E.; Deo G.; Juskelis M. V.; Weckhuysen B. M. Methanol Oxidation over Supported Vanadium Oxide Catalysts: New Fundamental Insights about Oxidation Reactions over Metal Oxide Catalysts from Transient and Steady State Kinetics. In Dynamics of Surfaces and Reaction Kinetics in Heterogeneous Catalysis; Froment G. F.; Waugh K. C., Eds.; Elsevier Science, 1997; pp 305–314.

[ref34] Kropp T.; Paier J.; Sauer J. Oxidative Dehydrogenation of Methanol at Ceria-Supported Vanadia Oligomers. J. Catal. 2017, 352, 382–387. 10.1016/j.jcat.2017.06.011.

[ref35] Kropp T.; Paier J.; Sauer J. Support Effect in Oxide Catalysis: Methanol Oxidation on Vanadia/Ceria. J. Am. Chem. Soc. 2014, 136, 14616–14625. 10.1021/ja508657c. 25275568

[ref36] Shapovalov V.; Fievez T.; Bell A. T. A Theoretical Study of Methanol Oxidation Catalyzed by Isolated Vanadia Clusters Supported on the (101) Surface of Anatase. J. Phys. Chem. C 2012, 116, 18728–18735. 10.1021/jp302862q.

[ref37] Goodrow A.; Bell A. T. A Theoretical Investigation of the Selective Oxidation of Methanol to Formaldehyde on Isolated Vanadate Species Supported on Titania. J. Phys. Chem. C 2008, 112, 13204–13214. 10.1021/jp801339q.

[ref38] Yun D.; Wang Y.; Herrera J. E. Ethanol Partial Oxidation over VO x /TiO 2 Catalysts: The Role of Titania Surface Oxygen on Vanadia Reoxidation in the Mars-van Krevelen Mechanism. ACS Catal. 2018, 8, 4681–4693. 10.1021/acscatal.7b03327.

[ref39] Ober P.; Rogg S.; Hess C. Direct Evidence for Active Support Participation in Oxide Catalysis: Multiple Operando Spectroscopy of VOx/Ceria. ACS Catal. 2020, 10, 2999–3008. 10.1021/acscatal.9b05174.

[ref40] Brückner A.; Kondratenko E. Simultaneous Operando EPR/UV-Vis/Laser-Raman Spectroscopy - A Powerful Tool for Monitoring Transition Metal Oxide Catalysts during Reaction. Catal. Today 2006, 113, 16–24. 10.1016/j.cattod.2005.11.006.

[ref41] Dinse A.; Carrero C.; Ozarowski A.; Schomäcker R.; Schlögl R.; Dinse K. P. Characterization and Quantification of Reduced Sites on Supported Vanadium Oxide Catalysts by Using High-Frequency Electron Paramagnetic Resonance. ChemCatChem 2012, 4, 641–652. 10.1002/cctc.201100412.

[ref42] Meitzner G. In Situ XAS Characterization of Heterogeneous Catalysts. In In-Situ Spectroscopy in Heterogeneous Catalysis; Haw J. F., Ed.; Wiley-VCH Verlag GmbH & Co. KGaA: Weinheim, FRG, 2002; pp 179–194.

[ref43] Sa J. Heterogeneous Catalysts. In High-Resolution XAS/XES; Sa J., Ed.; CRC Press, 2014; pp 169–194.

[ref44] Yano J.; Yachandra V. K. X-Ray Absorption Spectroscopy. Photosynth. Res. 2009, 102, 241–254. 10.1007/s11120-009-9473-8. 19653117 PMC2777224

[ref45] Zabilskiy M.; Sushkevich V. L.; Palagin D.; Newton M. A.; Krumeich F.; van Bokhoven J. A. The Unique Interplay between Copper and Zinc during Catalytic Carbon Dioxide Hydrogenation to Methanol. Nat. Commun. 2020, 11, 2409 10.1038/s41467-020-16342-1. 32415106 PMC7229192

[ref46] Imbao J.; van Bokhoven J. A.; Clark A.; Nachtegaal M. Elucidating the Mechanism of Heterogeneous Wacker Oxidation over Pd-Cu/Zeolite Y by Transient XAS. Nat. Commun. 2020, 11, 1118 10.1038/s41467-020-14982-x. 32111846 PMC7048791

[ref47] Marberger A.; Petrov A. W.; Steiger P.; Elsener M.; Kröcher O.; Nachtegaal M.; Ferri D. Time-Resolved Copper Speciation during Selective Catalytic Reduction of NO on Cu-SSZ-13. Nat. Catal. 2018, 1, 221–227. 10.1038/s41929-018-0032-6.

[ref48] Kopelent R.; van Bokhoven J. A.; Szlachetko J.; Edebeli J.; Paun C.; Nachtegaal M.; Safonova O. V. Catalytically Active and Spectator Ce 3+ in Ceria-Supported Metal Catalysts. Angew. Chem. 2015, 127, 8852–8855. 10.1002/ange.201503022. 26069026

[ref49] Safonova O. V.; Guda A.; Rusalev Y.; Kopelent R.; Smolentsev G.; Teoh W. Y.; van Bokhoven J. A.; Nachtegaal M. Elucidating the Oxygen Activation Mechanism on Ceria-Supported Copper-Oxo Species Using Time-Resolved X-Ray Absorption Spectroscopy. ACS Catal. 2020, 10, 4692–4701. 10.1021/acscatal.0c00551.

[ref50] Wachs I. E. Raman and IR Studies of Surface Metal Oxide Species on Oxide Supports: Supported Metal Oxide Catalysts. Catal. Today 1996, 27, 437–455. 10.1016/0920-5861(95)00203-0.

[ref51] Gao X.; Bare S. R.; Fierro J. L. G.; Banares M. A.; Wachs I. E. Preparation and In-Situ Spectroscopic Characterization of Molecularly Dispersed Titanium Oxide on Silica. J. Phys. Chem. B 1998, 102, 5653–5666. 10.1021/jp981423e.

[ref52] Clark A. H.; Steiger P.; Bornmann B.; Hitz S.; Frahm R.; Ferri D.; Nachtegaal M. Fluorescence-Detected Quick-Scanning X-Ray Absorption Spectroscopy. J. Synchrotron Radiat. 2020, 27, 681–688. 10.1107/S1600577520002350. 32381768 PMC7285694

[ref53] Chiarello G. L.; Nachtegaal M.; Marchionni V.; Quaroni L.; Ferri D. Adding Diffuse Reflectance Infrared Fourier Transform Spectroscopy Capability to Extended X-Ray-Absorption Fine Structure in a New Cell to Study Solid Catalysts in Combination with a Modulation Approach. Rev. Sci. Instrum. 2014, 85, 074102 10.1063/1.4890668. 25085153

[ref54] Clark A. H.; Imbao J.; Frahm R.; Nachtegaal M. ProQEXAFS: A Highly Optimized Parallelized Rapid Processing Software for QEXAFS Data. J. Synchrotron Radiat. 2020, 27, 551–557. 10.1107/S1600577519017053. 32153297 PMC7064099

[ref55] Jaumot J.; de Juan A.; Tauler R. MCR-ALS GUI 2.0: New Features and Applications. Chemom. Intell. Lab. Syst. 2015, 140, 1–12. 10.1016/j.chemolab.2014.10.003.

[ref56] Gao X.; Bare S. R.; Weckhuysen B. M.; Wachs I. E. In Situ Spectroscopic Investigation of Molecular Structures of Highly Dispersed Vanadium Oxide on Silica under Various Conditions. J. Phys. Chem. B 1998, 102, 10842–10852. 10.1021/jp9826367.

[ref57] Voronov A.; Urakawa A.; van Beek W.; Tsakoumis N. E.; Emerich H.; Rønning M. Multivariate Curve Resolution Applied to in Situ X-Ray Absorption Spectroscopy Data: An Efficient Tool for Data Processing and Analysis. Anal. Chim. Acta 2014, 840, 20–27. 10.1016/j.aca.2014.06.050. 25086889

[ref58] van Stokkum I. H. M.; Mullen K. M.; Mihaleva V. V. Global Analysis of Multiple Gas Chromatography-Mass Spectrometry (GC/MS) Data Sets: A Method for Resolution of Co-Eluting Components with Comparison to MCR-ALS. Chemom. Intell. Lab. Syst. 2009, 95, 150–163. 10.1016/j.chemolab.2008.10.004.

[ref59] Wong J.; Lytle F. W.; Messmer R. P.; Maylotte D. H. K-Edge Absorption Spectra of Selected Vanadium Compounds. Phys. Rev. B 1984, 30, 5596–5610. 10.1103/PhysRevB.30.5596.

[ref60] Giuli G.; Paris E.; Mungall J.; Romano C.; Dingwell D. V Oxidation State and Coordination Number in Silicate Glasses by XAS. Am. Mineral. 2004, 89, 1640–1646. 10.2138/am-2004-11-1208.

[ref61] Yamamoto T. Assignment of Pre-Edge Peaks in K-Edge x-Ray Absorption Spectra of 3d Transition Metal Compounds: Electric Dipole or Quadrupole?. X-Ray Spectrom. 2008, 37, 572–584. 10.1002/xrs.1103.

[ref62] Rees J. A.; Wandzilak A.; Maganas D.; Wurster N. I. C.; Hugenbruch S.; Kowalska J. K.; Pollock C. J.; Lima F. A.; Finkelstein K. D.; DeBeer S. Experimental and Theoretical Correlations between Vanadium K-Edge X-Ray Absorption and K β Emission Spectra. J. Biol. Inorg. Chem. 2016, 21, 793–805. 10.1007/s00775-016-1358-7. 27251139 PMC4989026

[ref63] Sutton S. R.; Karner J.; Papike J.; Delaney J. S.; Shearer C.; Newville M.; Eng P.; Rivers M.; Dyar M. D. Vanadium K Edge XANES of Synthetic and Natural Basaltic Glasses and Application to Microscale Oxygen Barometry. Geochim. Cosmochim. Acta 2005, 69, 2333–2348. 10.1016/j.gca.2004.10.013.

[ref64] Chaurand P.; Rose J.; Briois V.; Salome M.; Proux O.; Nassif V.; Olivi L.; Susini J.; Hazemann J. L.; Bottero J. Y. New Methodological Approach for the Vanadium K-Edge X-Ray Absorption near-Edge Structure Interpretation: Application to the Speciation of Vanadium in Oxide Phases from Steel Slag. J. Phys. Chem. B 2007, 111, 5101–5110. 10.1021/jp063186i. 17429991

[ref65] Mansour A. N.; Smith P. H.; Baker W. M.; Balasubramanian M.; McBreen J. In Situ XAS Investigation of the Oxidation State and Local Structure of Vanadium in Discharged and Charged V2O5 Aerogel Cathodes. Electrochim. Acta 2002, 47, 3151–3161. 10.1016/S0013-4686(02)00234-7.

[ref66] Rossignol C.; Ouvrard G. General Behavior upon Cycling of LiNiVO4 as Battery Electrode. J. Power Sources 2001, 97–98, 491–493. 10.1016/S0378-7753(01)00517-1.

[ref67] McKeown D. A.; Muller I. S.; Matlack K. S.; Pegg I. L. X-Ray Absorption Studies of Vanadium Valence and Local Environment in Borosilicate Waste Glasses. MRS Online Proc. Libr. 2002, 713, 132 10.1557/proc-713-jj13.2.

[ref68] Nadjafi M.; Abdala P. M.; Verel R.; Hosseini D.; Safonova O. V.; Fedorov A.; Müller C. R. Reducibility and Dispersion Influence the Activity in Silica-Supported Vanadium-Based Catalysts for the Oxidative Dehydrogenation of Propane: The Case of Sodium Decavanadate. ACS Catal. 2020, 10, 2314–2321. 10.1021/acscatal.9b04752.

[ref69] Silversmit G.; van Bokhoven J. A.; Poelman H.; van Der Eerden A. M. J.; Marin G. B.; Reyniers M. F.; De Gryse R. The Structure of Supported and Unsupported Vanadium Oxide under Calcination, Reduction and Oxidation Determined with XAS. Appl. Catal., A 2005, 285, 151–162. 10.1016/j.apcata.2005.02.018.

[ref70] Trummer D.; Searles K.; Algasov A.; Guda S. A.; Soldatov A. V.; Ramanantoanina H.; Safonova O. V.; Guda A. A.; Copéret C. Deciphering the Phillips Catalyst by Orbital Analysis and Supervised Machine Learning from Cr Pre-Edge XANES of Molecular Libraries. J. Am. Chem. Soc. 2021, 143, 7326–7341. 10.1021/jacs.0c10791. 33974429

[ref71] Nakamura Y.; Murayama T.; Ueda W. Reduced Vanadium and Molybdenum Oxides Catalyze the Equivalent Formation of Ethane and Acetaldehyde from Ethanol. ChemCatChem 2014, 6, 741–744. 10.1002/cctc.201300991.

[ref72] Malmusi A.; Velasquez Ochoa J.; Tabanelli T.; Basile F.; Lucarelli C.; Agnoli S.; Carraro F.; Granozzi G.; Cavani F. Ethanol Aerobic and Anaerobic Oxidation with FeVO4 and V2O5 Catalysts. Appl. Catal., A 2019, 570, 139–147. 10.1016/j.apcata.2018.11.013.

[ref73] Newton M. A.; Knorpp A. J.; Meyet J.; Stoian D.; Nachtegaal M.; Clark A. H.; Safonova O. V.; Emerich H.; van Beek W.; Sushkevich V. L.; van Bokhoven J. A. Unwanted Effects of X-Rays in Surface Grafted Copper(II) Organometallics and Copper Exchanged Zeolites, How They Manifest, and What Can Be Done about Them. Phys. Chem. Chem. Phys. 2020, 22, 6826–6837. 10.1039/d0cp00402b. 32186570

[ref74] Yun D.; Song Y.; Herrera J. E. Supported Vanadium Oxide Clusters in Partial Oxidation Processes: Catalytic Consequences of Size and Electronic Structure. ChemCatChem 2017, 9, 3655–3669. 10.1002/cctc.201700503.

[ref75] Rossi T. C.; Grolimund D.; Nachtegaal M.; Cannelli O.; Mancini G. F.; Bacellar C.; Kinschel D.; Rouxel J. R.; Ohannessian N.; Pergolesi D.; Lippert T.; Chergui M. X-Ray Absorption Linear Dichroism at the Ti K Edge of Anatase TiO2 Single Crystals. Phys. Rev. B 2019, 100, 245207 10.1103/PhysRevB.100.245207. PMC7064109 32153281

[ref76] Farges F.; Brown G. E.; Rehr J. J. Ti-Edge XANES Studies of Ti Coordination and Disorder in Oxide Compounds: Comparison between Theory and Experiment. Phys. Rev. B 1997, 56, 1809–1819. 10.1103/PhysRevB.56.1809.

[ref77] Hanley T. L.; Luca V.; Pickering I.; Howe R. F. Structure of Titania Sol-Gel Films: A Study by X-Ray Absorption Spectroscopy. J. Phys. Chem. B 2002, 106, 1153–1160. 10.1021/jp012225h.

[ref78] Luca V. Comparison of Size-Dependent Structural and Electronic Properties of Anatase and Rutile Nanoparticles. J. Phys. Chem. C 2009, 113, 6367–6380. 10.1021/jp808358v.

[ref79] Chen L. X.; Rajh T.; Wang Z.; Thurnauer M. C. XAFS Studies of Surface Structures of TiO2 Nanoparticles and Photocatalytic Reduction of Metal Ions. J. Phys. Chem. B 1997, 101, 10688–10697. 10.1021/jp971930g.

[ref80] Chen L. X.; Rajh T.; Jäger W.; Nedeljkovic J.; Thurnauer M. C. X-Ray Absorption Reveals Surface Structure of Titanium Dioxide Nanoparticles. J. Synchrotron Radiat. 1999, 6, 445–447. 10.1107/S090904959801591X. 15263339

[ref81] Rittmann-Frank M. H.; Milne C. J.; Rittmann J.; Reinhard M.; Penfold T. J.; Chergui M. Mapping of the Photoinduced Electron Traps in TiO 2 by Picosecond X-Ray Absorption Spectroscopy. Angew. Chem. 2014, 126, 5968–5972. 10.1002/ange.201310522. 24820181

[ref82] Müller P.; Hermans I. Applications of Modulation Excitation Spectroscopy in Heterogeneous Catalysis. Ind. Eng. Chem. Res. 2017, 56, 1123–1136. 10.1021/acs.iecr.6b04855.

