# Supplementary material for: Redox Dynamics of Active VOx Sites Promoted by TiOx during Oxidative Dehydrogenation of Ethanol Detected by Operando Quick XAS
Source: JACS Au. 2022 Mar 14;2(3):762–76. doi: 10.1021/jacsau.2c00027 (PMC8977985; doi:10.1021/jacsau.2c00027)
Supplement: Supplementary file 1 — au2c00027_si_001.pdf [file au2c00027_si_001.pdf]

Supplementary Information

Redox dynamics of active VO_x sites promoted by TiO_x during oxidative dehydrogenation of ethanol detected by *operando* quick XAS

Anna Zabilska[a,b], Adam H. Clark[a], Benjamin M. Moskowitz[c], Israel E. Wachs[c], Yuya Kakiuchi[d], Christophe Copéret[d], Maarten Nachtegaal[a], Oliver Kröcher[a,b], Olga V. Safonova[a]*

[a]Paul Scherrer Institute, 5232 Villigen, Switzerland

[b]École Polytechnique Fédérale de Lausanne, 1015 Lausanne, Switzerland

[c]*Operando* Molecular Spectroscopy & Catalysis Laboratory, Department of Chemical & Biomolecular Engineering, Lehigh University, Bethlehem, PA 18015, USA

[d]Department of Chemistry and Applied Biosciences, ETH Zürich, CH-8093 Zürich, Switzerland

*olga.safonova@psi.ch

Contents

1. Supplementary Methods 3

Surface VO_x and TiO_x density 3

1.1. *In situ* diffuse reflectance (DR) UV-Vis spectroscopy 3

1.2. *In situ* Raman spectroscopy 4

1.3. X-ray beam damage tests at the V K absorption-edge 5

1.4. Time-resolved Ti K-edge XAS experiments and data analysis 5

1.5. X-ray Crystallographic Analysis 6

1.6. Determination of the number of components in V K-edge XANES data 7

1.7. MCR ALS of V K-edge XANES spectra 7

1.8. Synthesis of V-containing references 7

1.9. Pre-edge analysis of V K-edge XAS 9

1.10. Quantitate analysis of the products by IR spectroscopy 9

1.11. Quantification of the rates of V^{5+} consumption and V^{3+} and V^{4+} formation upon oxygen switch off10

1.12. Quantification of the rates of V^{4+} re-oxidation by molecular oxygen upon oxygen switch on 11

2. Supplementary Figures 11

3. Supplementary Tables 35

4. Supplementary Results and Discussion 36

4.1. *In situ* DR UV-Vis spectroscopy 36

4.2. *In situ* Raman spectroscopy 41

4.3. Determining the number of components in V K-edge XANES 43

4.4. X-ray Crystallographic Analysis of tailored molecular V-references 46

4.5. Assignment of MCR-resolved V K-edge spectra 48

4.6. V^{4+} re-oxidation rate 52

4.7. Activity of titanium during alcohol oxidation 54

5. Supplementary References 57

1. Supplementary Methods

Surface VO_x and TiO_x density

The surface density of TiO_x (SD_{TiOx}) was found in the next way:

$$SD_{TiO_x} = \frac{\%_{TiO_2} \cdot N_A}{M_{TiO_2} \cdot SA \cdot 10^{20}}$$

Where $\%_{TiO2}$ is a TiO_2 loading in %, N_A –Avogadro's constant, M_{TiO2} – TiO_2 molar mass in g · mol^{-1}, SA – surface area of the catalyst after TiO_x supporting.

The surface density of VO_x (SD_{VOx}) was found in the next way:

$$SD_{VO_x} = \frac{2 \cdot \%_{V_2O_5} \cdot N_A}{M_{V_2O_5} \cdot SA \cdot 10^{20}}$$

Where $\%_{V2O5}$ is a V_2O_5 loading in %, N_A –Avogadro's constant, M_{V2O5} – V_2O_5 molar mass in g · mol^{-1}, SA – surface area of the catalyst after VO_x supporting.

1.1. *In situ* diffuse reflectance (DR) UV-Vis spectroscopy

Silica was used as a diluent to ensure DR UV-vis signal linearity, with signal intensity $F(\rho)_\infty \leq 0.5$.[1] Fumed silica (Cab-O-Sil EH-5, Cabot) was dispersed in deionized (DI) water (18.2 MΩ, MilliQ), dried at 120 °C in static air for 16 h in a muffle furnace (48000, Thermolyne), crushed in an agate mortar, and then calcined in the muffle furnace at 500 °C for 4 h (ramp rate 1 °C/min) in 100 cm^3/min flowing dry air (AI D300, Airgas). The silica was then crushed in an agate mortar and sieved to retain 75 – 125 μm aggregates. Catalyst samples were separately crushed in an agate mortar and sieved to retain 75 – 125 μm aggregates. 15% TiO_2/SiO_2 was mixed with the silica diluent in a vial to achieve a catalyst weight fraction of 0.125 and thoroughly mixed by inverting 50 times. 5% V_2O_5/15% TiO_2/SiO_2 was mixed with the silica diluent in a vial to achieve a catalyst weight fraction of 0.0625 and thoroughly mixed by inverting 50 times. The silica diluent was also separately examined.

UV-vis spectra of the samples were collected with an Agilent Cary 5000 UV-vis-NIR spectrophotometer in diffuse reflectance mode with a Harrick Scientific Praying Mantis accessory. Polytetrafluoroethylene powder (35 μm, Sigma Aldrich) was used as a reflectance standard. Approximately 10-20 mg of sample was loaded into an *in situ* environmental cell (HVC-DR2, Harrick Scientific) equipped with CaF_2 windows into a sample cup padded with quartz wool (AI-4033, Altamira Instruments) and 316SS mesh. The sample thermocouple

(KMQSS-032G-6, Omega Engineering) was custom fit to directly probe the sample bed through a spare port and sealed with a PTFE ferrule.

For dehydration experiments, the samples were treated in 30 cm^3/min 10% O_2/Ar (Certified Standard, Praxair) at 400 °C for 1 h (ramp rate 10 °C/min) and cooled to 120 °C (ramp rate 10 °C/min) for spectral acquisition. Spectra (200 – 800 nm, 1 nm resolution, 0.1 s/nm averaging) were collected in double-beam mode with full slit height, a slit beam width of 4, and a 1.5 absorbance filter in the reference beam. The reflectance spectra were analyzed using the Kubelka-Munk formalism to convert the reflectance into the equivalent absorption coefficient, $F(\rho_\infty)$, according to:[2]

$$F(\rho_\infty) = \frac{(1-\rho_\infty)^2}{2\rho_\infty}$$

where

$$\rho_\infty = \frac{\rho_{sample}}{\rho_{reflectance\ standard}}$$

The direct allowed optical band gap (E_g) was determined from the x-intercept of the linear fit of a plot of $(F(\rho_\infty)h\nu)^2$ versus $h\nu$ with data points selected to yield the largest positive slope, where $h\nu$ is the incident photon energy. The absolute error in the band gap was set to 0.1 eV owing to the uncertainty in the manual fitting process. Spectra were further analyzed by calculating a difference spectrum between the 5% V_2O_5/15% TiO_2/SiO_2 spectrum and the 15% TiO_2/SiO_2 subtrahend spectrum, where spectra were first constant-baseline corrected and a 1.23x multiplication factor was applied to the 15% TiO_2/SiO_2 spectrum. The direct band gap of this difference spectrum was calculated as mentioned above. Finally, the 5% V_2O_5/15% TiO_2/SiO_2 and 15% TiO_2/SiO_2 spectra were analyzed by least-squares fitting in Fityk version 1.3.1.[3] Spectra constant-baseline corrected and fit simultaneously with Gaussian peaks in the 50 000 - 20 000 cm^{-1} and 50 000 - 25 000 cm^{-1} ranges for 5% V_2O_5/15% TiO_2/SiO_2 and 15% TiO_2/SiO_2, respectively. All peak positions and FWHM values were constrained to be equal to other across the spectra, while peak areas were constrained to be non-negative. Finally, direct band gaps of the Gaussian peaks were calculated as mentioned above.

1.2. In situ Raman spectroscopy

The dehydrated Raman spectra of the 15% TiO_2/SiO_2 and 5% V_2O_5/15% TiO_2/SiO_2 samples were acquired with a Horiba LabRAM HR Evolution confocal Raman spectrometer. A He-Cd

laser source (Kimmon IK5751I-G) generated the 442 nm laser excitation with a filtered power output of 7.5 mW. A confocal microscope with a 50x objective (Olympus BX-30-LWD) was used to focus the laser onto the sample. The 520.7 cm^{-1} band of a silicon wafer standard was used to calibrate the spectrometer prior to spectral acquisition. A spectral resolution of <2.7 cm^{-1} was achieved while averaging 5 scans (120 s/scan) and using a 100 μm hole. The *in situ* Raman spectra were collected in a high-temperature reaction cell (CCR1000, Linkam Scientific) controlled with a high temperature controller (TMS94, Linkam Scientific). The inlet 10%O_2/Ar gas mixture used in the dehydration treatment was supplied using a Brooks mass flow controller (5850E) with a set point of 30 cm^3/min. Approximately 30 mg of each sample was loaded onto a quartz wool-padded sample cup before sealing the reaction cell. The samples were heated to 450 °C at a rate of 10 °C min^{-1}, where they were dehydrated for 1h before cooling down to 120 °C at a rate of 10 °C min^{-1}, and finally acquiring the spectra.

1.3. X-ray beam damage tests at the V K absorption-edge

To make sure that there is no influence of the X-ray beam on the state of the measured VO_x species, we performed a series of dedicated experiments at relevant temperatures. We have performed oxygen cut-off experiments with a different X-ray beam size (from 300 x 200 $μm^2$ to 500 x 400 $μm^2$) controlled by the Rh-coated toroidal mirror. In addition, we have done a periodical X-ray beam on/off switching experiments. Prior to the beam-switching experiment, the catalyst was exposed for 30 min to steady-state conditions at 160 °C in 1.6 vol% EtOH, 6vol% O_2 in He. We started the data acquisition while exposing the sample to the ethanol-oxygen mixture (1.6 vol% EtOH, 6.4 vol% O_2 in He) at 160 °C. After 10 min, we switched the beam off by closing a shutter, while continuing the data acquisition, and switched it on again after 10 min. For the analysis, 20 XAS scans were averaged and normalized as described in the main text. The time interval without beam was removed from the data file. The processed spectra from both types of experiments were analyzed using a linear combination fit using the XAS spectra resolved by MCR-ALS (obtained previously from TPR and oxygen switch-off experiments) in the ProQEXAFS software.[4]

1.4. Time-resolved Ti K-edge XAS experiments and data analysis

We performed two types of transient experiments, named modulation excitation (ME) experiments while monitoring the Ti K-edge XAS. The first type of experiment was similar to the oxygen cut-off experiments performed at the V K-edge: for 5 min the catalyst was exposed to the ethanol-oxygen flow (1.6 vol% EtOH, 6.4 vol% O_2 in He) and for the next 5 min in an

oxygen-free ethanol containing mixture (1.6 vol% EtOH in He). At each temperature, 10 switching cycles were performed. In the second type of experiment, the oxygen-containing mixture (6.4 vol% O_2 in He) was alternated with the ethanol-containing mixture (1.6 vol% EtOH in He). The gases were switched every 5 min and 10 cycles were performed at each temperature.

The four Ti K-edge XAS spectra were averaged, background-subtracted, and normalized to the edge jump of one. In the pre-edge region, the background subtraction was performed using a linear function in the interval of -61.0 – -8.7 eV (relative to E_0=4966.0 eV). For normalization in the post-edge region, a cubic polynomial function in the 100.0 – 492.6 eV interval was used. A bespoke python script was used for the phase-sensitive detection (PSD) analysis and period averaging. PSD allows the analysis of tiny changes caused by periodic perturbation in the composition of gas mixture.[5] It transforms time-resolved spectra into phase-resolved spectra and can be described by the following equation:

$$I(\varphi^{PSD}) = \frac{2}{T}\int_0^T I(t)\sin(kwt + \varphi^{PSD})\,dt, \qquad (2)$$

where ω is the frequency of the stimulation, k is the demodulation index, φ^{PSD} is the phase angle for demodulation k, $I(t)$ and $I(\varphi^{PSD})$ are the intensities in the time and phase domains, respectively.

Thus, in each ME XAS experiment the initially measured 12 000 spectra (10 cycles of 10 min with 2 scans/s), after processing and period averaging ended up in one averaged gas switching cycle consisting of 300 spectra with a time resolution of 2 s.

To receive an average in the certain feed composition spectra (which were used for differential spectra calculations), we used phase-averaged spectra with serial numbers 14-149 and 164-299 (spectrum 0 corresponds to gas switching); fist 15 spectra under each (new) conditions were not considered to exclude the transitional period from one condition to another (Figure S3).

1.5. X-ray Crystallographic Analysis

For single crystal X-ray diffraction analysis, suitable crystals were placed onto MiTeGen loop pins coated in paratone oil and mounted under a flow of nitrogen at 100 K on a Bruker Smart Apex II or Bruker D8 Venture diffractometer with CCD area detector using Mo Kα irradiation. Using Olex2[6] the structures were solved with the Super flip package[7,8] and refined using SHELXL.[9] All non-hydrogen atoms were refined with anisotropic displacement parameters. Hydrogen atoms were placed in ideal positions and refined as riding atoms.

1.6. Determination of the number of components in V K-edge XANES data

To determine the number of spectral components in the time-resolved V K-edge XANES spectra, we analysed the MCR-ALS results, received using different number of components. SIMPLISMA approach implemented in the MCR-ALS GUI2.0 software[10] helped us to guess the initial spectra. The lack of fit in each solution was quantified according to this equation:

$$Lack\ of\ fit\ (\%) = 100\sqrt{\frac{\sum_{i,j} e_{ij}^2}{\sum_{i,j} d_{ij}^2}} \qquad \text{(S1)}$$

where d_{ij} is the element of the data matrix **D**, e_{ij} is the element of the residual matrix **E** (see equation 1 in the main text).

1.7. MCR ALS of V K-edge XANES spectra

The final solution was found with the use of initial guesses of V^{5+}, V^{4+}, and V^{3+} component spectra. The first and the last spectra from the EtOH TPR experiment were used as the initial guesses for V^{5+} and V^{3+}, respectively. As the initial guess for V^{4+}, we used the last spectrum measured in ethanol during oxygen cut-off experiments at 210 °C.

1.8. Synthesis of V-containing references

The reference compounds vanadium (III) oxide (**V_2O_3**, Chemie Brunschwig, >95%), vanadium (IV) oxide (**V_2O_4**, Fisher Scientific), vanadium (V) oxide (**V_2O_5**, Chemie Brunschwig, >99%), vanadium (V) oxytriisopropoxide (**$VO(O\text{-}i\text{-}C_3H_7)_3$**, Sigma-Aldrich), vanadium (IV) sulphate oxide hydrate (**$VOSO_4$**, VWR), vanadyl (IV) acetylacetonate (**$VO(C_5H_7O_2)_2$**, Chemie Brunschwig, >99%), vanadium (III) 2,4-pentanedionate (**$V(C_5H_7O_2)_3$**, Chemie Brunschwig, >97%), ammonium metavanadate (**NH_4VO_3**, VWR), potassium metavanadate (**KVO_3**, VWR), sodium orthovanadate (**Na_3VO_4**, Lucerna Chem), titanium (III) oxide (Ti_2O_3, Fisher Scientific, >99,8%), titanium (IV) oxide rutile (**TiO_2** (rutile), Sigma Aldrich, >99.9%) and titanium (IV) oxide anatase (TiO_2 (anatase), Sigma Aldrich, >99.8%) were purchased.

The sodium metavanadates **β-$NaVO_3$** and **α-$NaVO_3$**, sodium decavanadate **$Na_6V_{10}O_{28}\cdot18H_2O$** and **$Na_{1.164}V_3O_8$** were prepared following published methods.[11]

Ammonium decavanadate (**$(NH_4)_6V_{10}O_{28}\cdot6H_2O$**) was prepared using the method described in [12].

V(=O)(OTBOS)$_3$ complex was synthesized according to the procedure reported in [13].

V(OTBOS)$_4$. The titled complex was synthesized according to the reported procedure for analogous vanadium(IV) siloxide complexes.[14] A suspension of $VCl_4(thf)_2$ (300 mg, 0.890 mmol, synthesized according to the reported procedure[15]) in *n*-hexane/THF (1:1, 25 mL) was treated with Na(OTBOS)

(1.02 g, 3.56 mmol) at -78 °C. The solution was stirred for 2 hours at the same temperature to give clear blue solution. The solution was then further stirred overnight at room temperature followed by a reflux for 1.5 hours. All volatiles were removed under vacuum. Remaining sky-blue residue was extracted with pentane (40 mL). The extract was concentrated under vacuum to give $V(OTBOS)_4$ as blue-white powder. Yield: 787.8 mg, 80 %. ^1H NMR (300 MHz, C_6D_6) δ/ppm = 1.66 (br., ν1/2≈ 200 Hz).

$V(OTBOS)_3thf_2$. A solution of $VCl_3(thf)_3$ (2.00 g, 5.42 mmol, synthesized according to the literature procedure[16]) in toluene/THF (1:1, 100 mL) was treated with NaOSi(O^{t}Bu)$_3$ (4.66 g, 16.2 mmol, synthesized according to the literature procedure[17]) to give deep blue solution. The resulting solution was stirred overnight at room temperature. Solvent was removed under vacuum. Remaining purple solid was extracted with *n*-pentane (50 mL) and the blue-purple solution was concentrated under vacuum. Recrystallization in *n*-pentane gave pink crystals. Yield: 2.02 g, 38% yield. ^1H NMR (300 MHz, C_6D_6) δ/ppm = 1.66 (br., ν1/2≈ 1000 Hz).

Since the molecular structure of the complex **$V(OTBOS)_3thf_2$** was not obtained in good quality in XRD analysis, analogous V(OSi(OMes)(O^{t}Bu)$_2$)$_3$(thf)$_2$ was synthesized in the same manner and was analyzed.

V(OSi(OMes)(O^{t}Bu)$_2$)$_3$(thf)$_2$. A solution of $VCl_3(thf)_3$ (200 mg, 0.535 mmol, synthesized according to the literature procedure[16]) in toluene/THF (1:1, 10 mL) was treated with NaOSi(OMes)(O^{t}Bu)$_2$ (559 g, 1.60 mmol, synthesized according to the literature procedure[18]) to give deep purple solution. The resulting solution was stirred overnight at room temperature. Solvent was removed under vacuum. Remaining purple residue was extracted with *n*-pentane (30 mL) and the resulting purple solution was oncentrated under vacuum. Recrystallization in HMDSO gave pink crystals, which are suitable for XRD studies. Yield: 259.6 g, 42% yield. ^1H NMR (300 MHz, C_6D_6) δ/ppm = 1.62 (br., ν1/2≈ 800 Hz), 2.17 (br., ν1/2≈ 50 Hz), 6.81 (br., ν1/2≈ 100 Hz).

$V(OTBOS)_3(OPPh_3)$. A solution of $V(OTBOS)_3(thf)_2$ (98.0 mg, 0.100 mmol) in benzene (10 mL) was treated with triphenylphosphine oxide (27.8 mg, 0.100 mmol). The resulting greenish blue solution was stirred at room temperature for 2 hours. All volatiles were removed under vacuum to give green-blue powder. Yield: 95.6 mg, 85% yield. Recrystallization in *n*-pentane gave crystals with the same color, which are suitable for XRD studies. ^1H NMR (200 MHz, C_6D_6) δ/ppm = 1.60 (br., ν1/2≈ 1100 Hz), 7.00 (br., ν1/2≈ 80 Hz), 7.97 (br., ν1/2≈ 150 Hz). $^{31}P\{^1H\}$ NMR (81 MHz, C_6D_6) δ/ppm = 24.80 (br., ν1/2≈ 170 Hz).

1.9. Pre-edge analysis of V K-edge XAS

The area under the pre-edge peak, the position of the center of mass (centroid) and the half-edge step position (the energy at 0.5 a.u. adsorption) were calculated for each resolved and reference spectra. For a more accurate calculation of the pre-edge area and the pre-edge centroid position, cumulative distribution function (CDF, eq. S2) was used as a baseline to correct the edge rising (Figure S4). The edge jump was fitted with CDF with the use of the least square method (implemented on the base of Python). The CDF was fitted in two energy intervals: before the edge and at the edge raise; these intervals were chosen manually for every calculated spectrum, all of them are shown in Figures S5, S6. The resulting peak was integrated. In the case, if a spectrum contained an additional shoulder on the edge (B-peak in Figure S4 b), the peaks received after subtraction were fitted with multiple pseudo-Voight functions to identify and subtract the contribution of the edge shoulder (Figure S4 c).

$$f_{CDF} = \frac{1}{2}\left[1 + erf\left(\frac{x-\mu}{\sigma\sqrt{2}}\right)\right] \quad \text{(S2)}$$

Where x – the energy scale, μ - fitted parameter, defines the x position of f_{CDF}, σ – fitted parameter, defines the slope of $f_{CDF.}$

1.10. Quantitate analysis of the products by IR spectroscopy

Figure S7 shows the IR spectra of ethanol and possible catalytic products. For the quantitative analysis of ethanol and acetaldehyde concentrations during *operando* oxygen cut-off experiments, specific bands belonging exclusively to these molecules (1294–1181 cm^{-1} for ethanol, 1853–1658 cm^{-1} for acetaldehyde) were extracted and analyzed using ALS-MCR. A series of IR spectra corresponding to different concentrations of pure ethanol and acetaldehyde were also included in the experimental data set for the ALS-MCR analysis.[10] The applied constraints were: non-negativity of the spectral components and the sum of concentrations of all components < 1.

For the quantitative analysis of ethylene concentration (observed only in the TPR experiment), the intensity of the band at 948 cm^{-1} was analyzed. Since ethane does not show any bands, which do not overlap with the bands of the other products, its quantitative analysis was hindered. To follow the ethane formation in the TPR experiment, we used the intensity of the band at 3060 cm^{-1}. The IR spectra of carbon dioxide and carbon monoxide are also shown in Figure S7, however, their formation was not observed under catalytic conditions.

1.11. Quantification of the rates of V^{5+} consumption and V^{3+} and V^{4+} formation upon oxygen switch off

To estimate the initial rates of V^{5+} consumption and V^{4+} and V^{3+} formation after oxygen cut-off, we fitted the concentration profiles of the V^{5+}, V^{4+} and V^{3+} components with the following functions:

Linear:
$$\begin{cases} y = Y_0\,, t < t_0 \\ y = Y_0 + k(t - t_0), t \geq t_0 \end{cases} \quad \text{(S3)}$$

Single exponent:
$$\begin{cases} y = Y_0\,, t < t_0 \\ y = Y_0 + A\left(1 - e^{-\frac{t-t_0}{\tau}}\right), t \geq t_0 \end{cases} \quad \text{(S4)}$$

Double exponent:
$$\begin{cases} y = Y_0\,, t < t_0 \\ y = Y_0 + A_1\left(1 - e^{-\frac{t-t_0}{\tau_1}}\right) + A_2\left(1 - e^{-\frac{t-t_0}{\tau_2}}\right), t \geq t_0 \end{cases} \quad \text{(S5)}$$

where t is the time, t_0 is the time when the change in component concentration is observed (t_0 = 10 min for V^{4+} and V^{5+}, when oxygen is switched off; t_0=10.4 min for V^{3+}, since V^{3+} appears with a time delay), y is the fraction of certain vanadium species, Y_0, A, A_1, A_2, $\boldsymbol{\tau}$, $\boldsymbol{\tau_1}$, and $\boldsymbol{\tau_2}$ are the fitted parameters.

The constant component concentrations before oxygen is switched off ($t<t_0$) in every experiment were fitted as a plateau ($y=Y_0$). The interval when oxygen is switched off was fitted with linear, exponential, or double exponential functions. The fitting was implemented in the Origin software using piecewise PWL2 (for linear fitting), ExpAssosDelay1 (for single exponential function) and ExpAssosDelay2 (for double exponential function) functions.

The choice of the fitting function was done based on the data quality of the resolved concentration profiles. The fitting interval usually was 1-15 min, however, in several experiments, we had to limit it to 12 min to diminish the impact of noise arising from deeper vanadium reduction. Importantly, this limitation does not significantly influence the results, since the complementary concentration profiles ($V^{5+}\leftrightarrow V^{4+}$) usually were fitted in the whole time interval. The functions chosen to fit each concentration profile are shown in Table S2, the result of the fits are shown on Figures S15-S16.

After fitting, the derivative at the time t_0 (coincides with the time when oxygen is switched off for V^{4+} and V^{5+} components and 0.4 min later for V^{3+}) was taken and multiplied on the total amount of vanadium sites in the catalyst bed to receive rate in mol/min units.

1.12. Quantification of the rates of V^{4+} re-oxidation by molecular oxygen upon oxygen switch on

To estimate the rate of $V^{4+/3+}$ re-oxidation, initially we planned to fit V^{4+} and V^{3+} concentration profiles similarly to ones made upon oxygen switch-off. However, the noise in the data did not allow us to make reasonable fitting and we decided to use a complementary component (V^{5+}), which has a lower noise level. However, we could use only those temperatures where the fraction of produced V^{3+} is negligible (T=160-190 °C). Thus, calculated V^{5+} formation rates could be attributed exclusively to $V^{4+} \rightarrow V^{5+}$ process.

2. Supplementary Figures

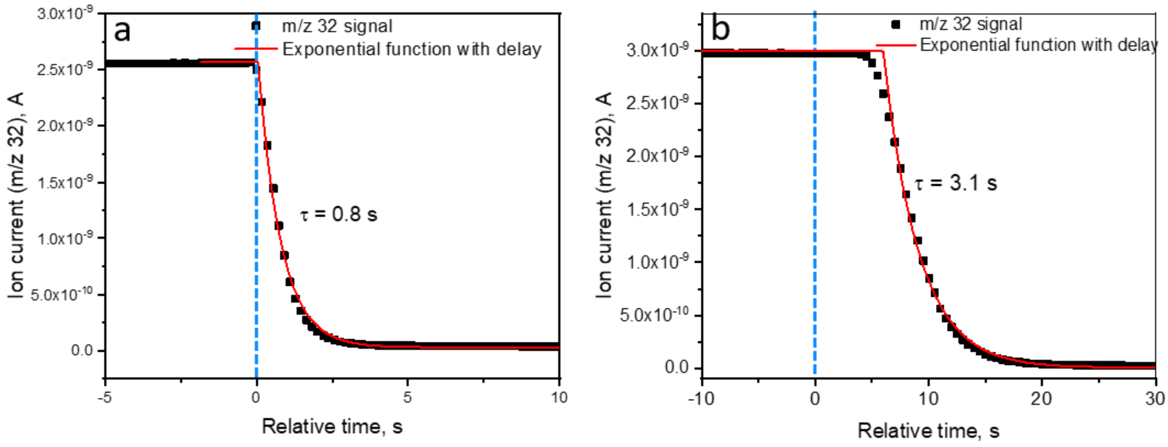

Figure S1. (a) The concentration profile of oxygen measured in the outlet of the *operando* cell in the oxygen cut-off experiment using a mass-spectrometer (the total flow is 50 mL/min, time 0 corresponds to oxygen switch-off time). (b) The concentration profile of oxygen measured at the exit of Bruker Alpha II FT-IR Spectrometer in the oxygen cut-off experiment using mass-spectrometer (the total flow is 50 mL/min, time 0 corresponds to oxygen switch-off time). These experiments were done with the use of mass the spectrometer OmniStar GSD 320, Pfeiffer.

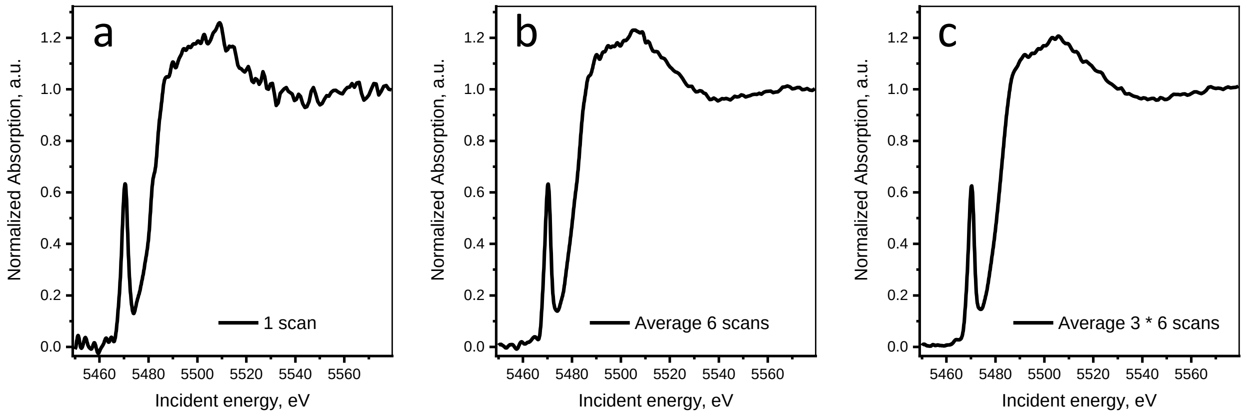

Figure S2. (a) Time-resolved data at V K-edge XAS measured during 1 scan (0.5 s), (b) after averaging 6 scans (3 s); (c) after averaging 18 scans (6 scans x 3 oxygen cut-off cycles).

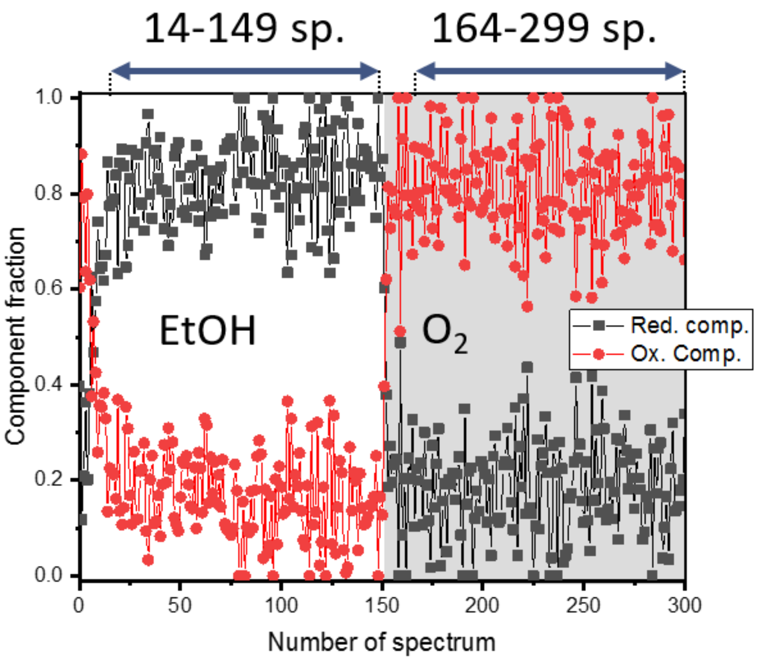

Figure S3. MCR-ALS resolved Ti K-edge XAS component profiles of phase-averaged spectra measured at 350 °C in an ethanol-oxygen switching experiment**.** The spectra with the serial numbers 14-149 and 164-299 were averaged to receive a spectrum in reducing or oxidizing environment, respectively. The component fraction profiles are shown here to explain the averaging procedure and to show the transitional period. This Figure does not carry any other informational load.

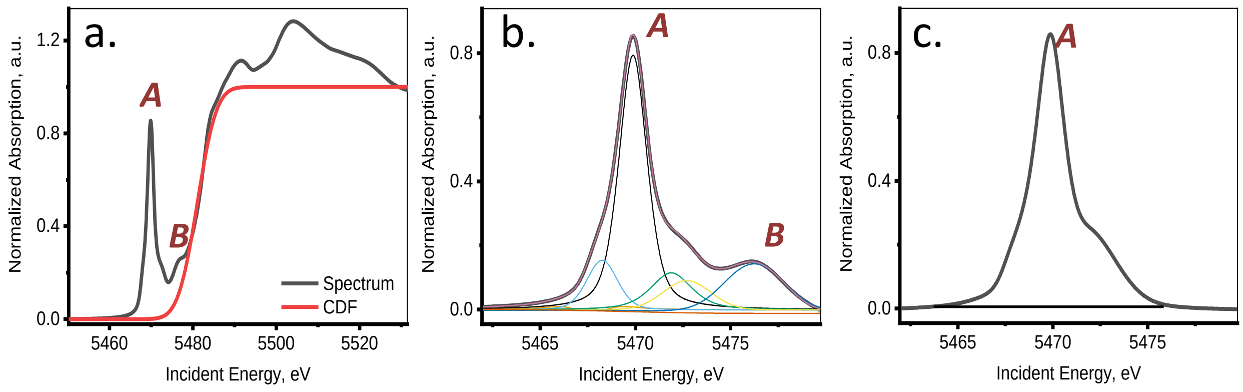

Figure S4. The procedure for the pre-edge extraction. a. Fitting of the edge with a cumulative distribution function; b. After the CDF subtraction, the resulting curve is fitted with multiple pseudo-Voight functions; c. The final pre-edge peak after subtraction of the additional pre-edge peak B.

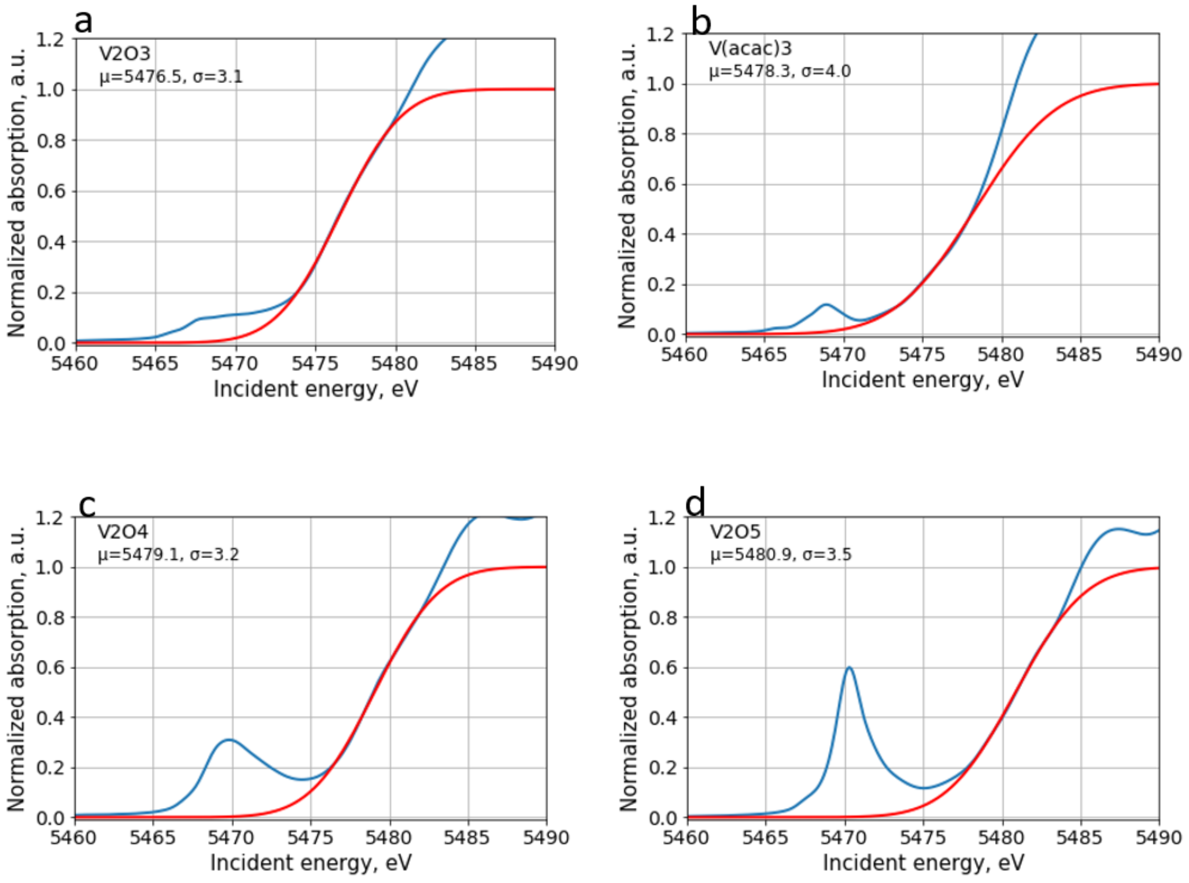

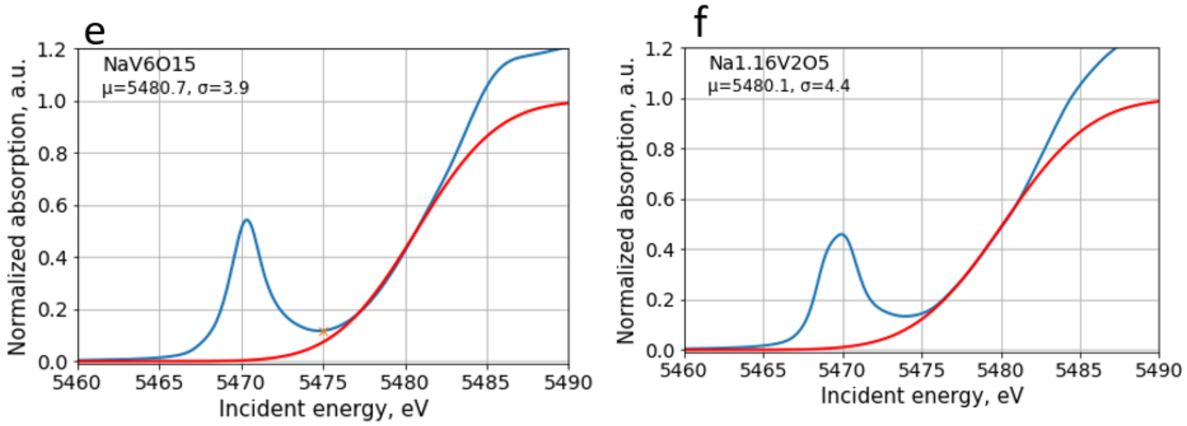

Figure S5. The V K-edge XANES edge rises of the references fitted by CDF. (a) V_2O_3, (b) $V(acac)_3$ (acac- acetylacetonate anion), (c) V_2O_4, (d) V_2O_5, (e) NaV_5O_{15}, (f) $Na_{1.164}V_2O_5$. The V K-edge XANES spectra are shown with blue curves, the fitted CDF with red curves.

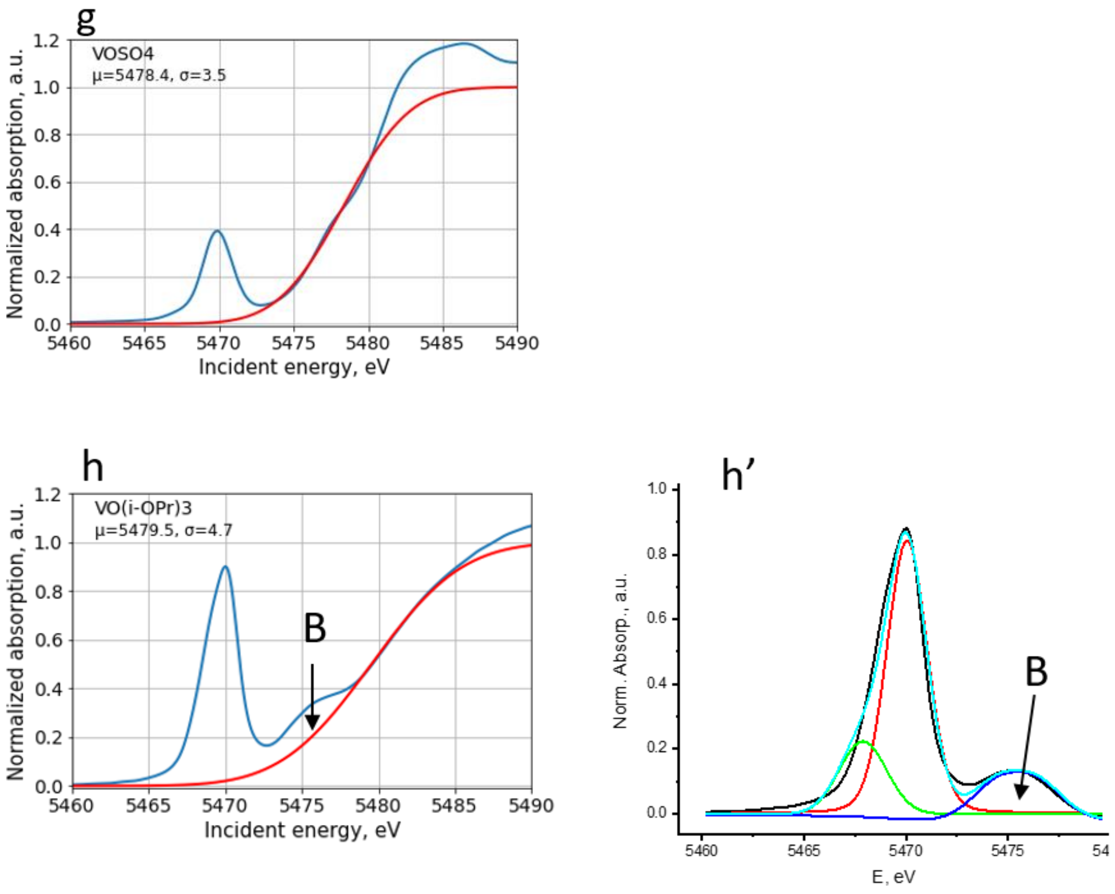

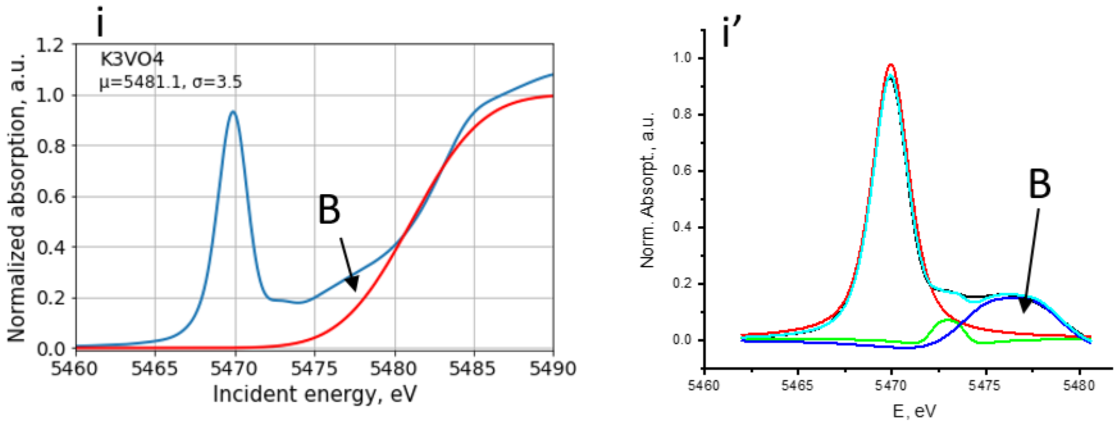

Figure S5. The V K-edge XANES edge rises of the references fitted by CDF. (g) $VOSO_4$, (h) VO(O-*i*-$C_3H_7)_3$, (i) K_3VO_4. The V K-edge XANES spectra are shown with blue curves, the fitted CDF with red curves. On the figures marked with apostrophe, we show the procedure of multiple peaks fitting to extract the contribution of the edge shoulder (marked everywhere with B), this peak was subtracted before integration.

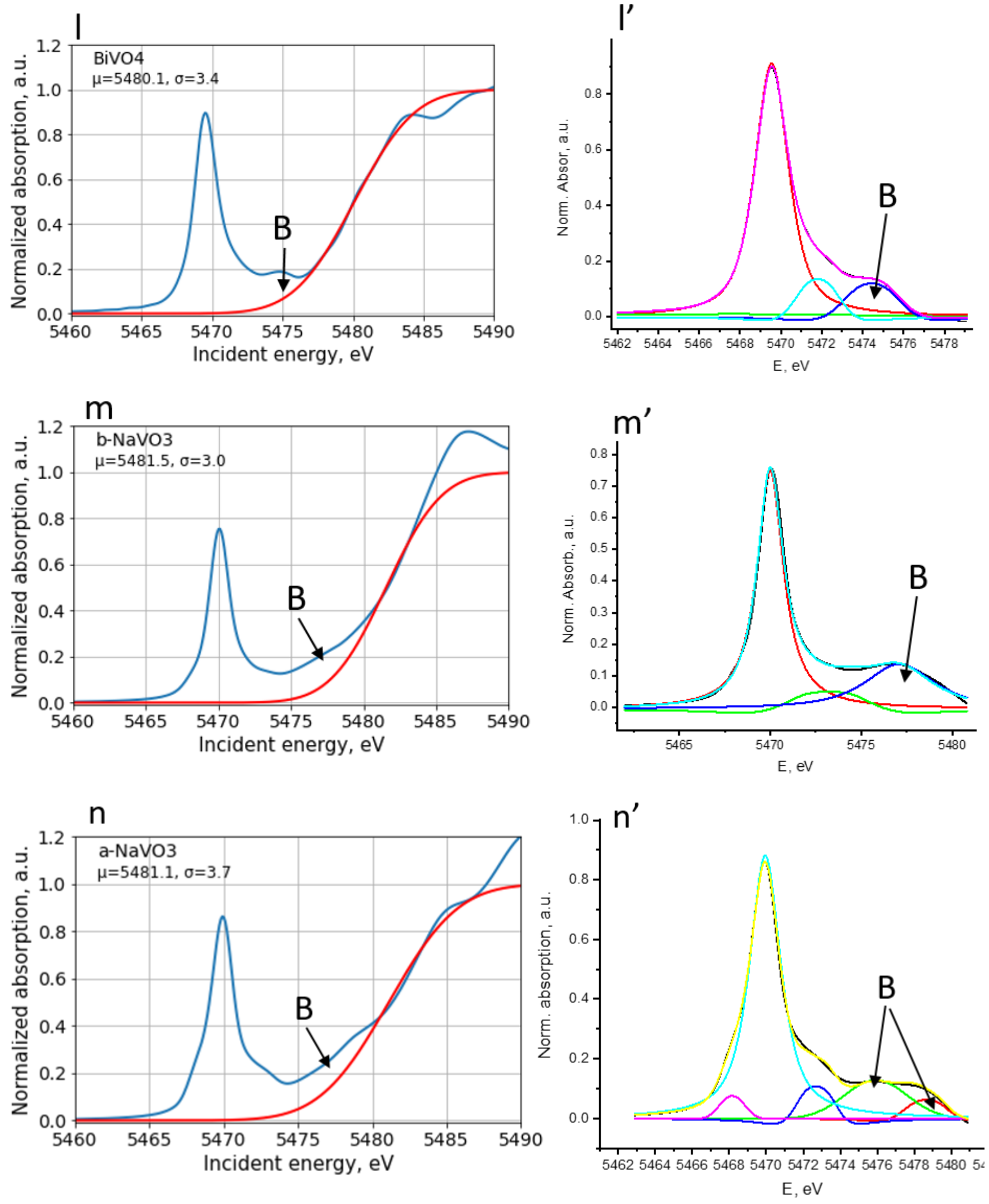

Figure S5. The V K-edge XANES edge rises of the references fitted by CDF. (l) $BiVO_4$, (m) β-$NaVO_3$, (n) α-$NaVO_3$. The V K-edge XANES spectra are shown with blue curves, the fitted CDF with red curves. On the figures marked with apostrophe, we show the procedure of multiple peaks fitting to extract the contribution of the edge shoulder (marked everywhere with B), this peak (-s) was (were) subtracted before integration.

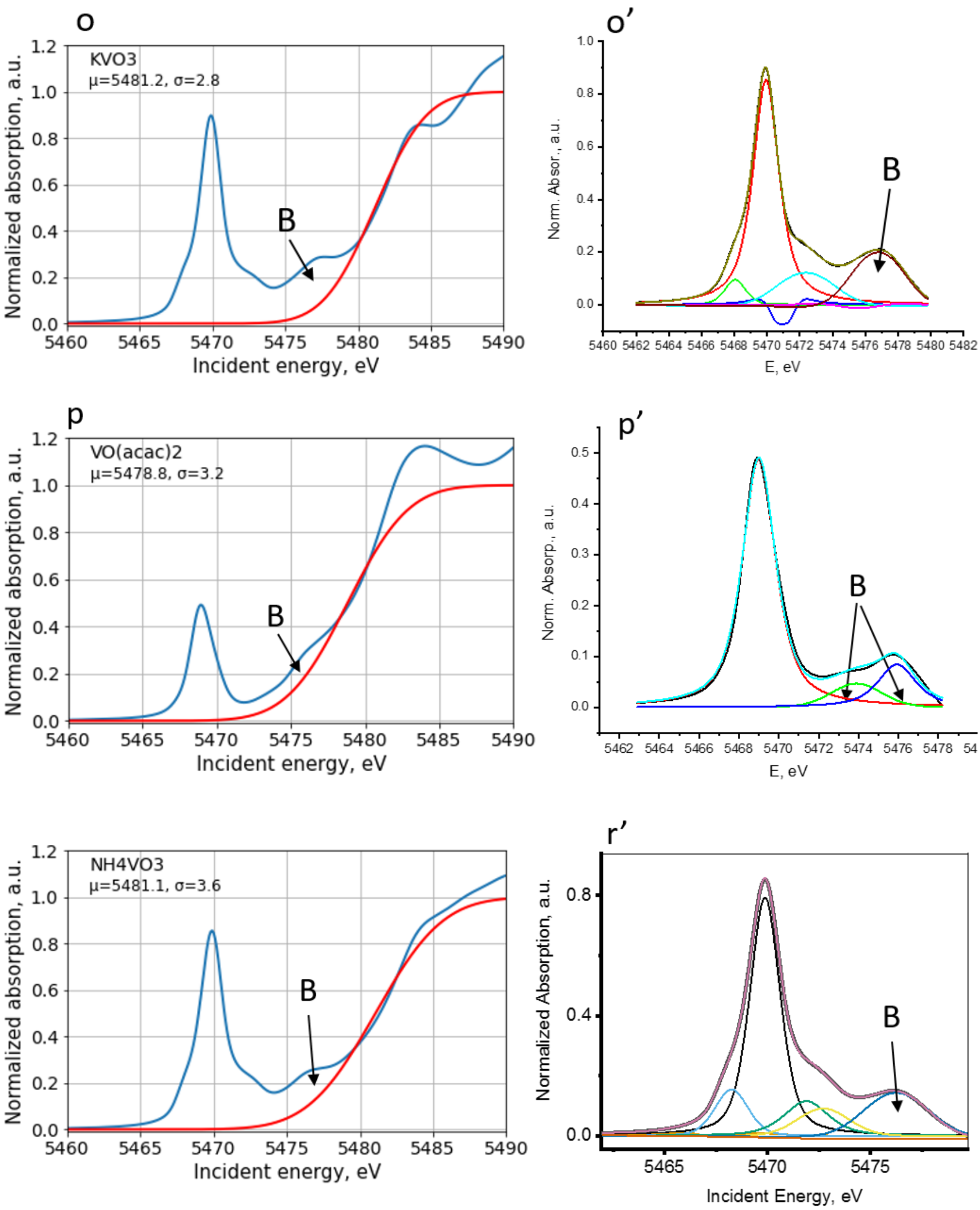

Figure S5. The V K-edge XANES edge rises of the references fitted by CDF. (o) KVO_3, (p) $VO(acac)_2$ (acac- acetylacetonate anion), (r) NH_4VO_3. The V K-edge XANES spectra are shown with blue curves, the fitted CDF with red curves. On the figures marked with apostrophe, we show

the procedure of multiple peaks fitting to extract the contribution of the edge shoulder (marked everywhere with B), this peak (-s) was (were) subtracted before integration.

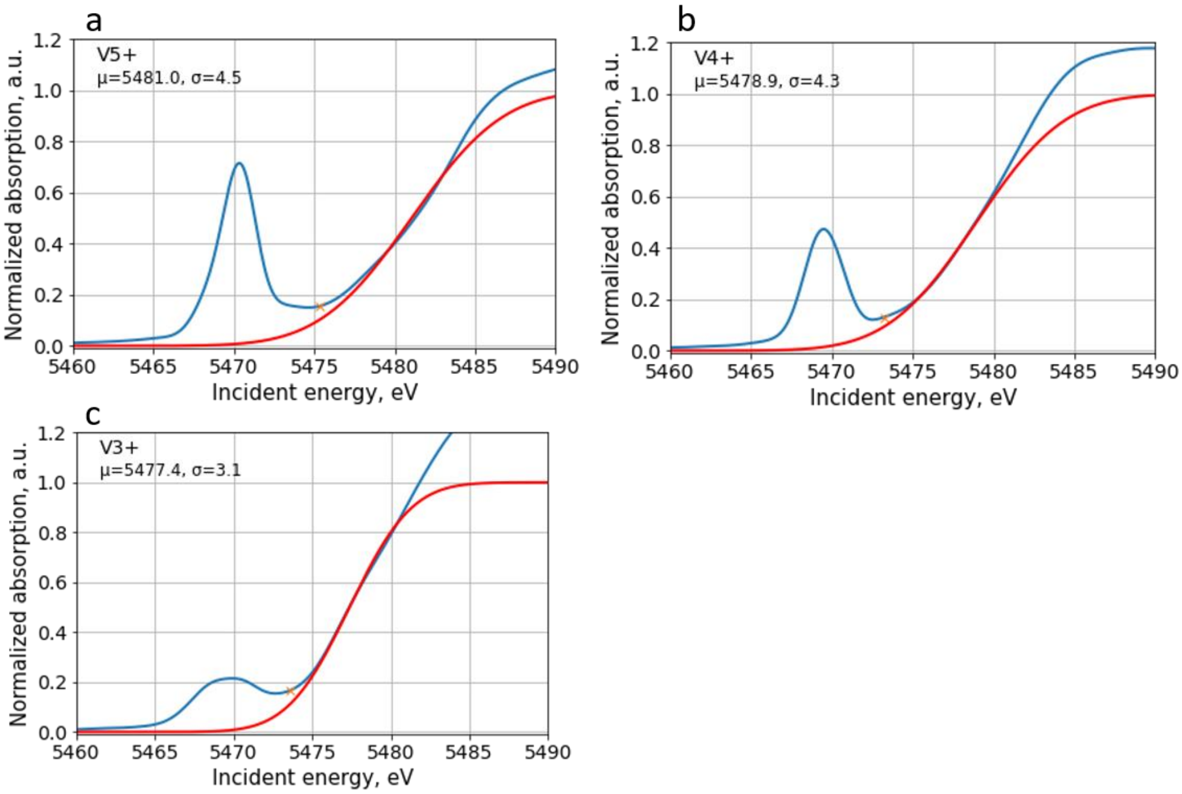

Figure S6. The V K-edge XANES edge rises of resolved components fitted by CDF. (a) V^{5+}, (b) V^{4+}, (c) V^{3+} components. The V K-edge XANES spectra are shown with blue curves, the fitted CDF with red curves. Orange crosses show the end of the integrated peak.

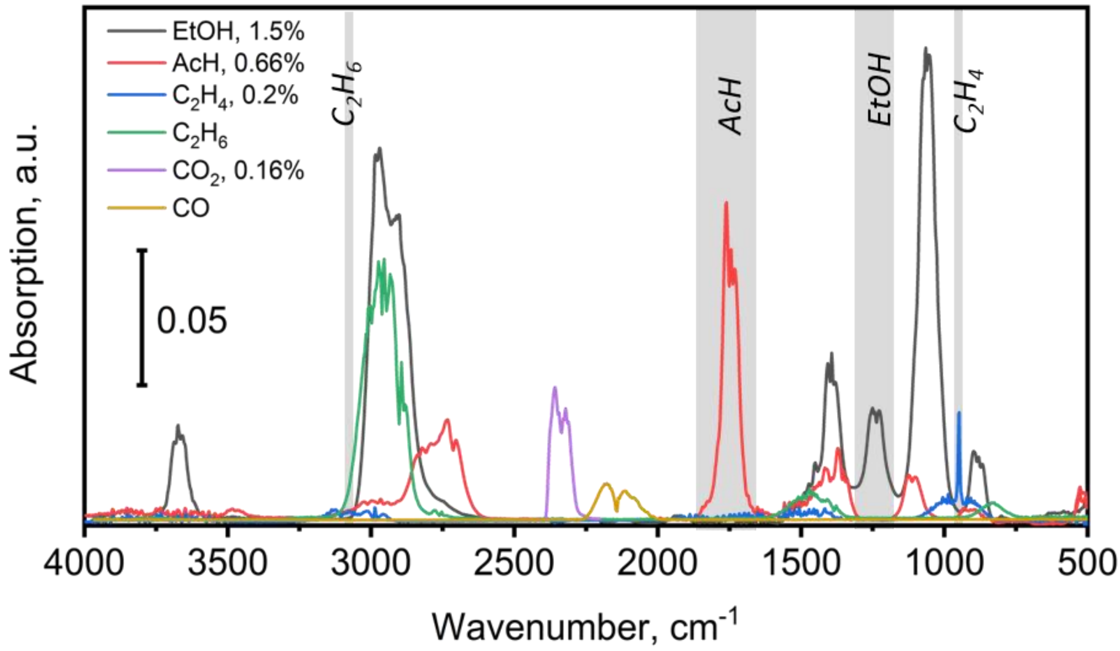

Figure S7. IR spectra of pure ethanol (1.6 vol%), acetaldehyde (0.66 vol%), ethylene (0.2 vol%), and carbon dioxide (0.16 vol%) obtained experimentally and the ones of ethane and carbon monoxide taken from the National Institutes of Standards and Technology (NIST) database. The bands used for quantitative analysis are highlighted with grey color.

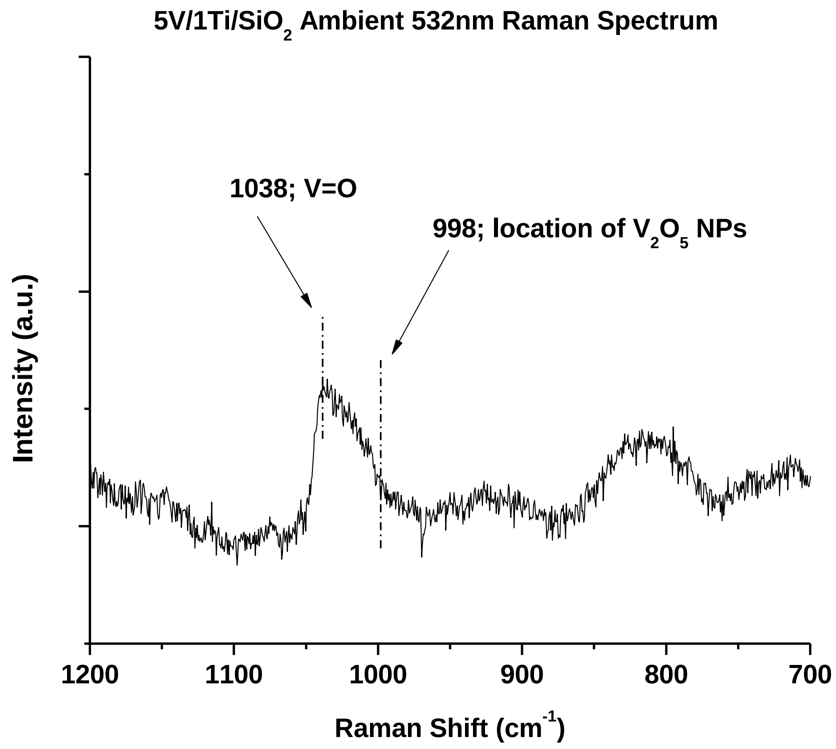

Figure S8 (a) Ambient 532 Raman spectrum of 5 wt% V_2O_5/ 1 wt% TiO_2/SiO_2 catalyst

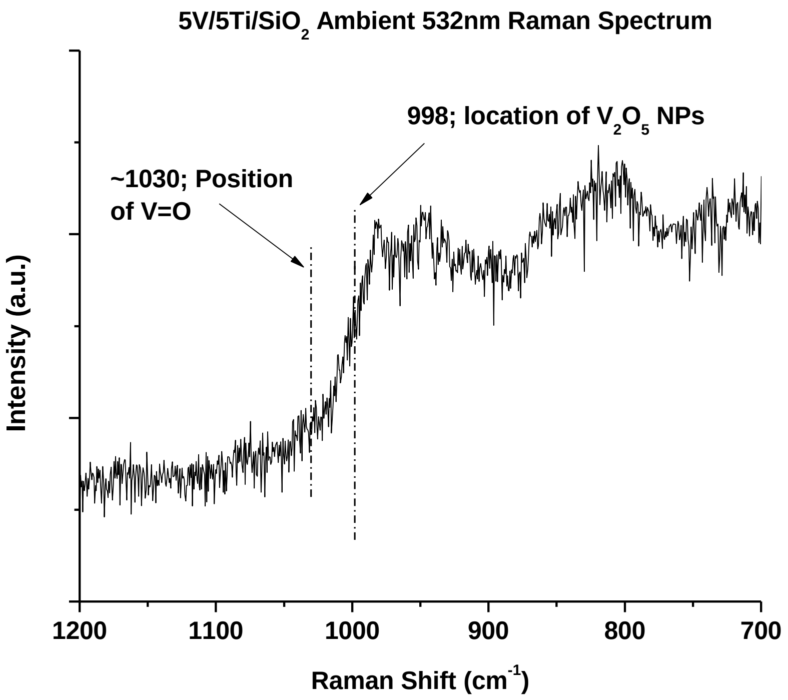

Figure S8 (b) Ambient 532 Raman spectrum of 5 wt% V_2O_5/ 5 wt% TiO_2/SiO_2 catalyst

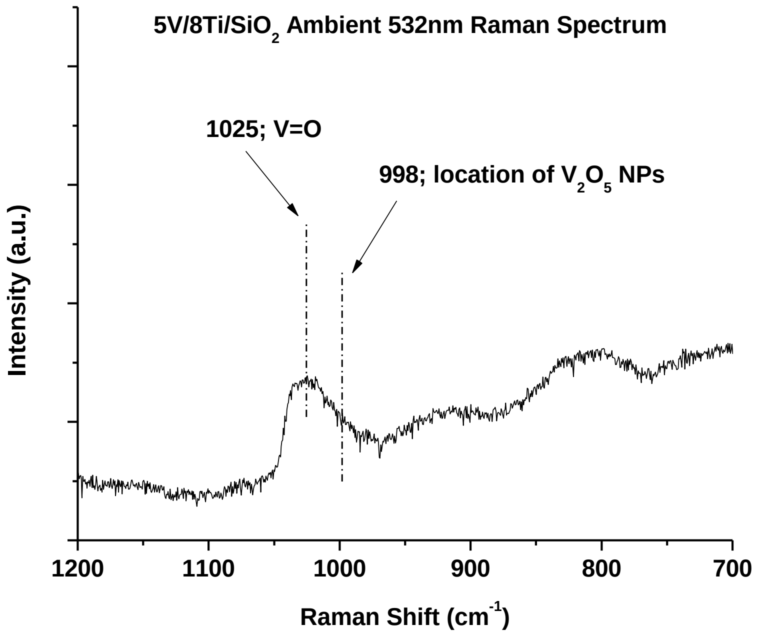

Figure S8 (c) Ambient 532 Raman spectrum of 5 wt% V_2O_5/ 8 wt% TiO_2/SiO_2 catalyst

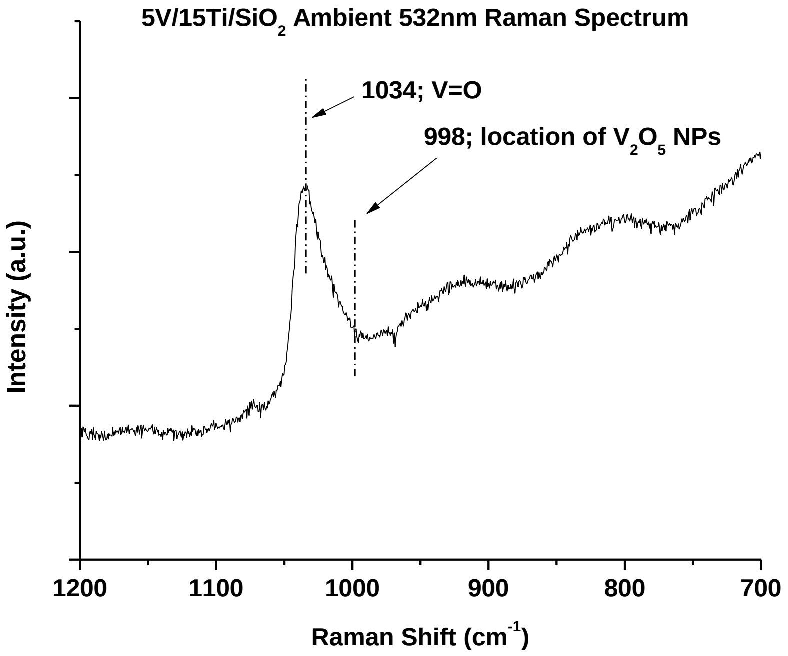

Figure S8 (d) Ambient 532 Raman spectrum of 5 wt% V_2O_5/ 15 wt% TiO_2/SiO_2 catalyst

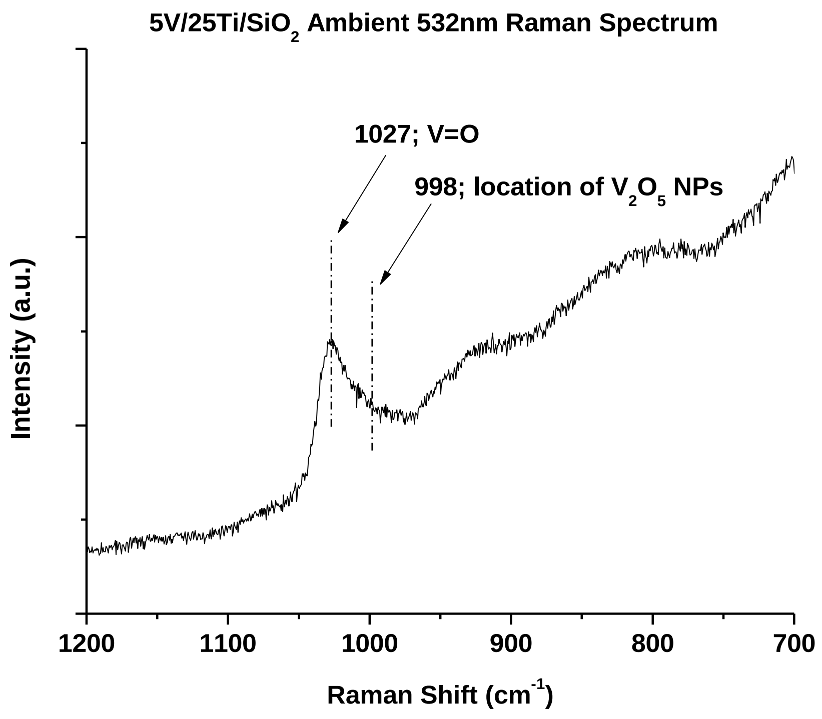

Figure S8 (e) Ambient 532 Raman spectrum of 5 wt% V_2O_5/ 25 wt% TiO_2/SiO_2 catalyst

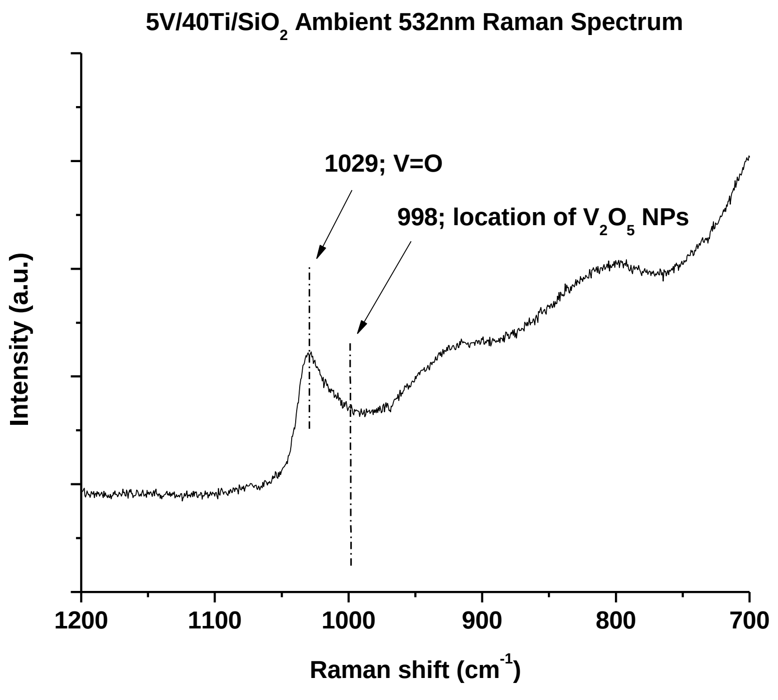

Figure S8 (f) Ambient 532 Raman spectrum of 5 wt% V_2O_5/ 40 wt% TiO_2/SiO_2 catalyst

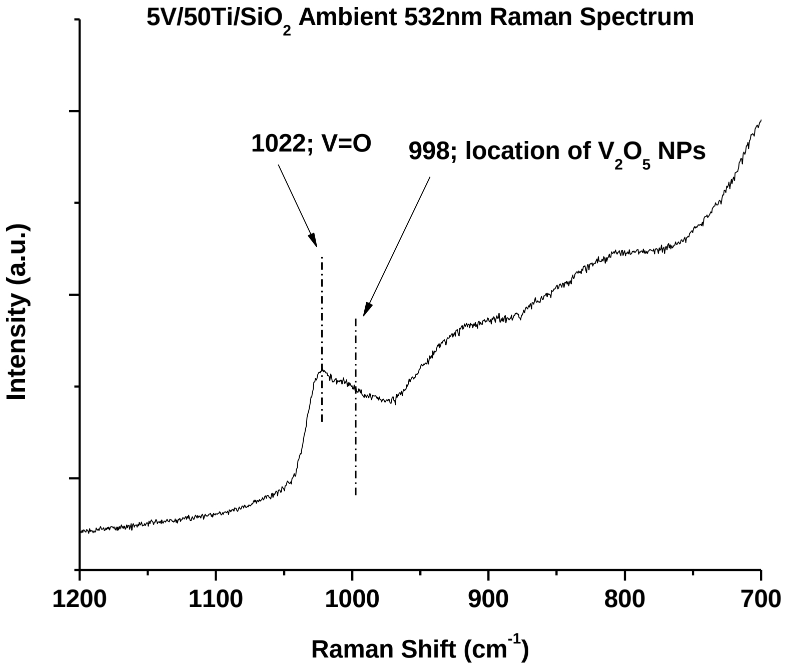

Figure S8 (j) Ambient 532 Raman spectrum of 5 wt% V_2O_5/ 50 wt% TiO_2/SiO_2 catalyst

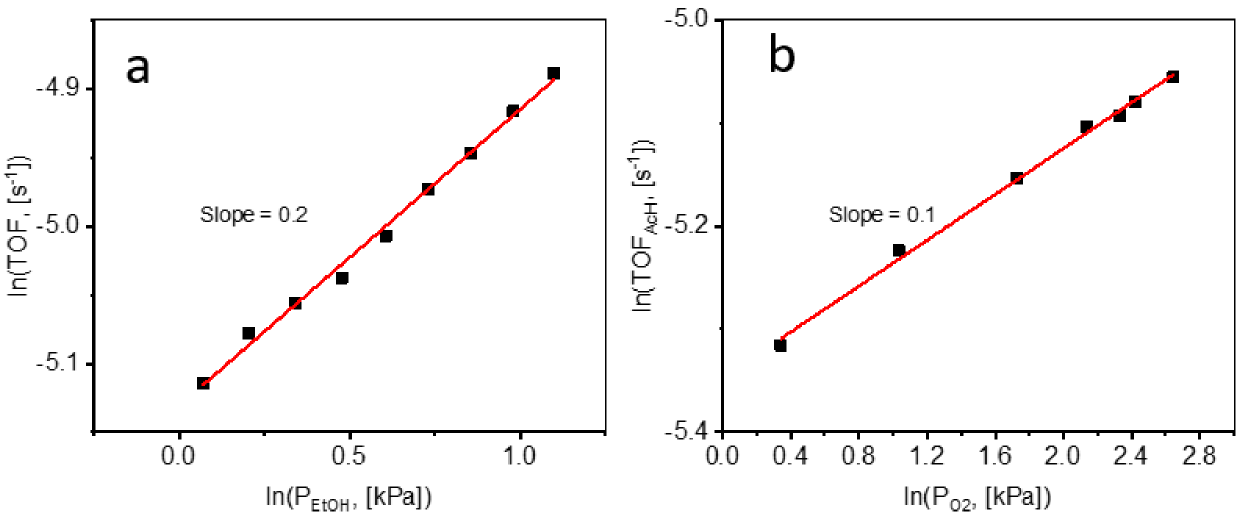

Figure S9. (a) The reaction order for ethanol in ethanol ODH over supported 5 wt% V_2O_5/ 15 wt% TiO_2/SiO_2 catalyst at 180 °C. The oxygen concentration was equal to 12 kPa; the concentration of ethanol was varied in the interval of 0.8-3 kPa. (b) The reaction order for oxygen in ethanol ODH over 5 wt% V_2O_5/ 15 wt% TiO_2/SiO_2 at 180 °C. The ethanol concentration was equal to 1.4 kPa; the concentration of oxygen was varied in the interval of 1.4-14 kPa.

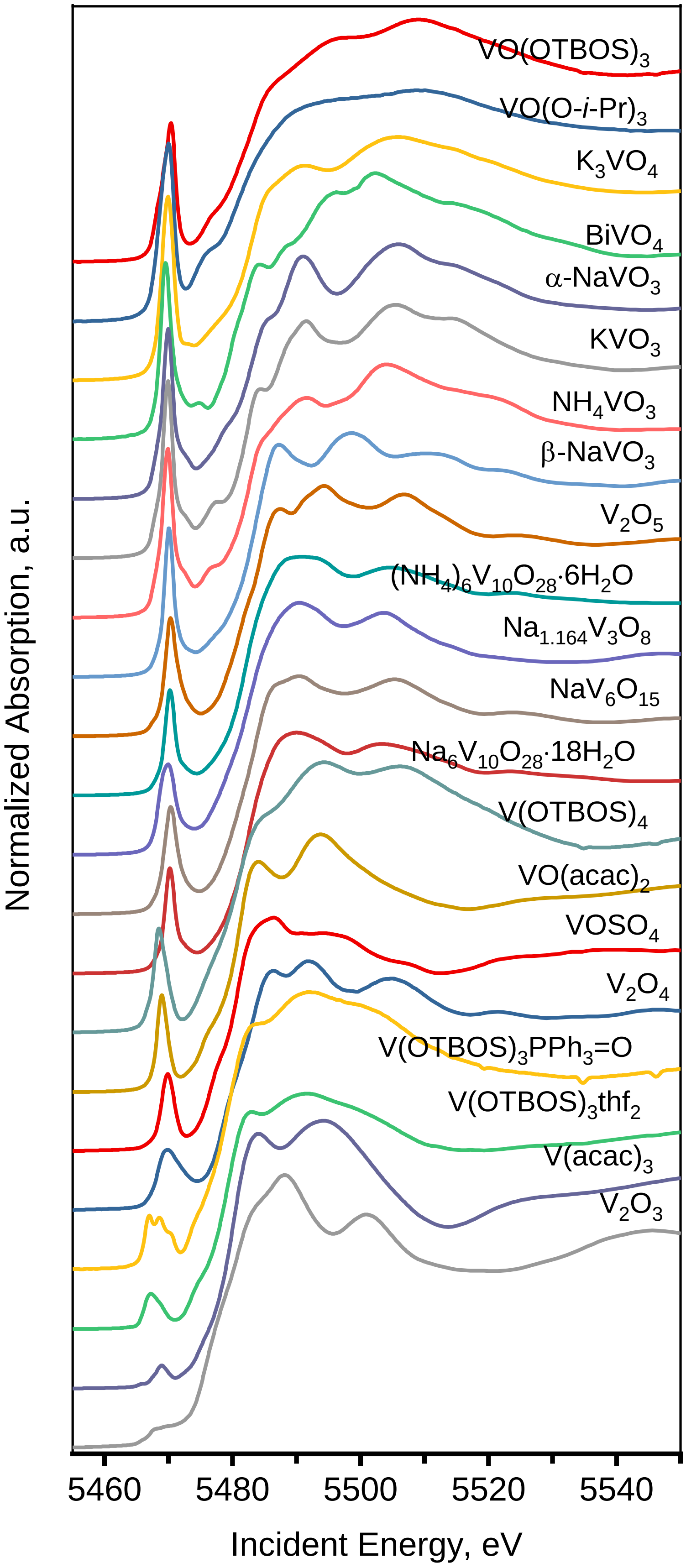

Figure S10. V K-edge XANES spectra of the vanadium-containing reference compounds.

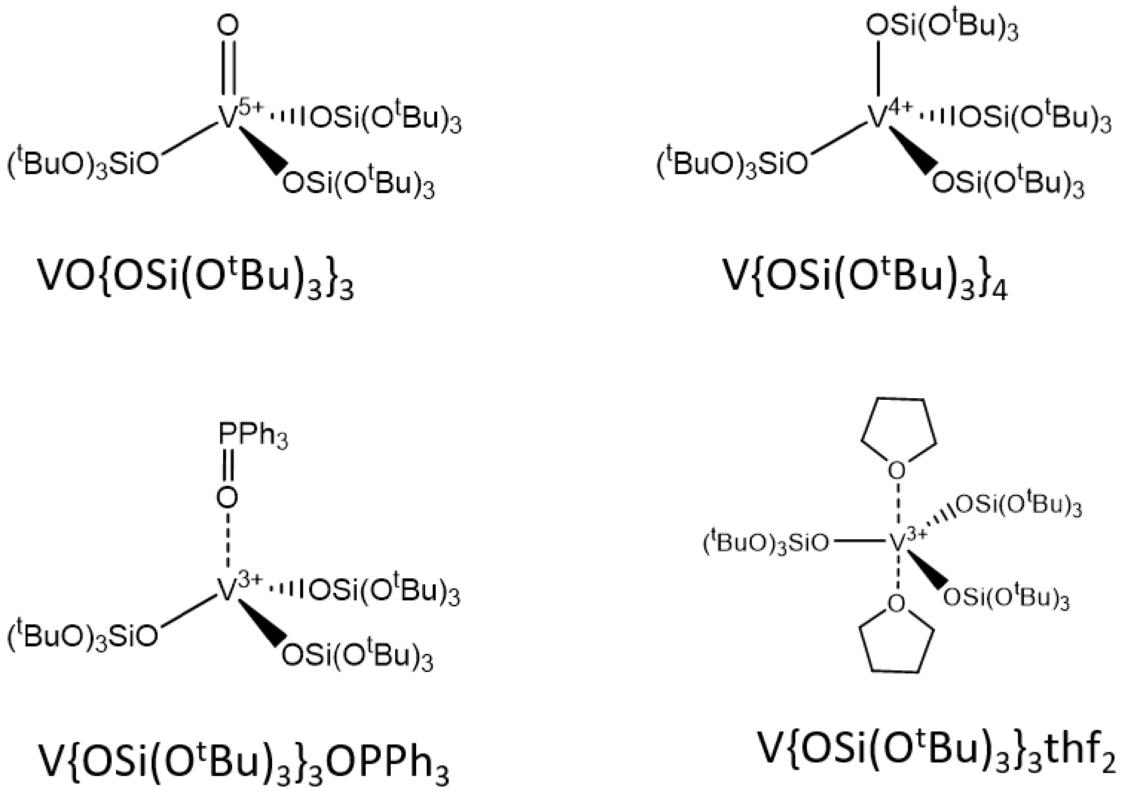

Figure S11. The structures of tailored molecular references.

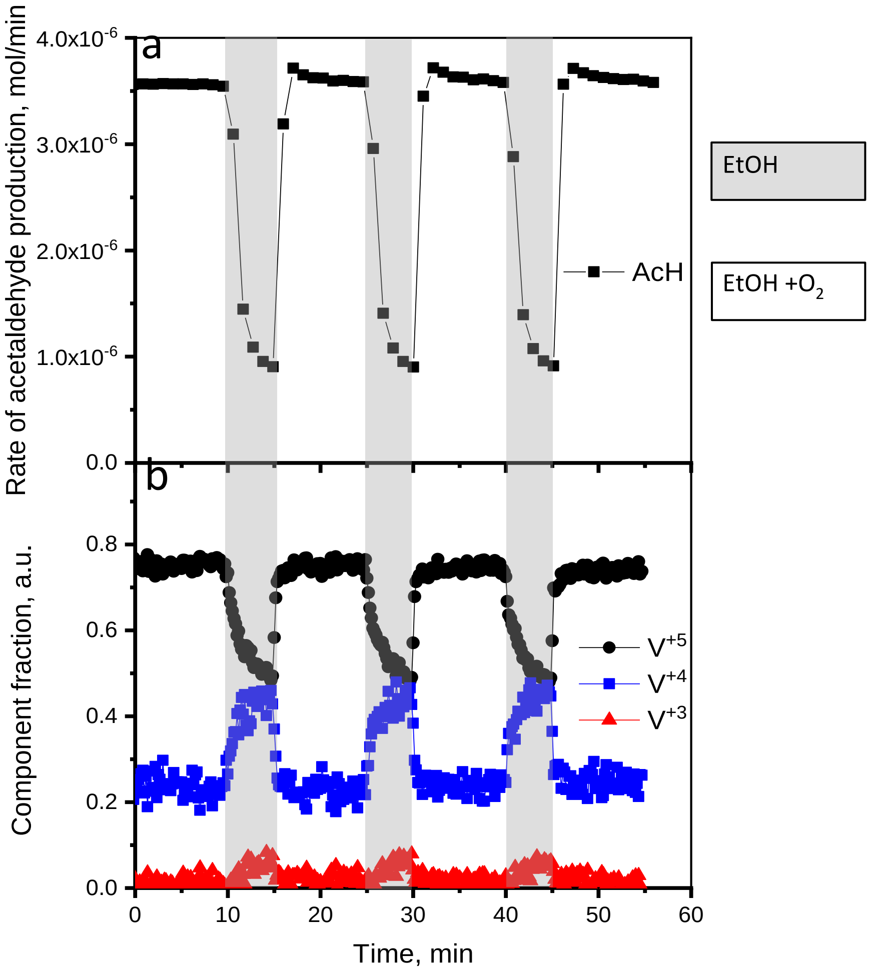

Figure S12. (a) The concentration of acetaldehyde detected by IR spectrometry in oxygen the cut-off experiment at 190 °C and (b) the corresponding V^{5+}, V^{4+}, and V^{3+} profiles. During the experiment, 1.6 vol% EtOH + 6.4 vol% O_2 in He and 5 vol% EtOH in He gas mixtures were periodically switched. The background acetaldehyde rate in oxygen-free conditions must be due to the oxygen leak through a graphite window.

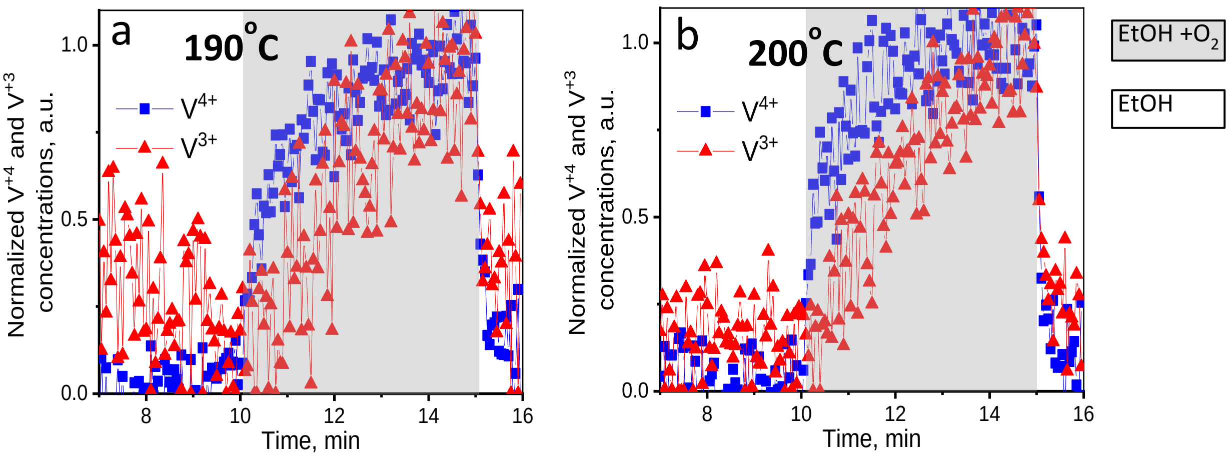

Figure S13. The relative changes in V^{4+} and V^{3+} concentrations during the oxygen cut-off experiments at (a) 190 and (b) 200 °C.

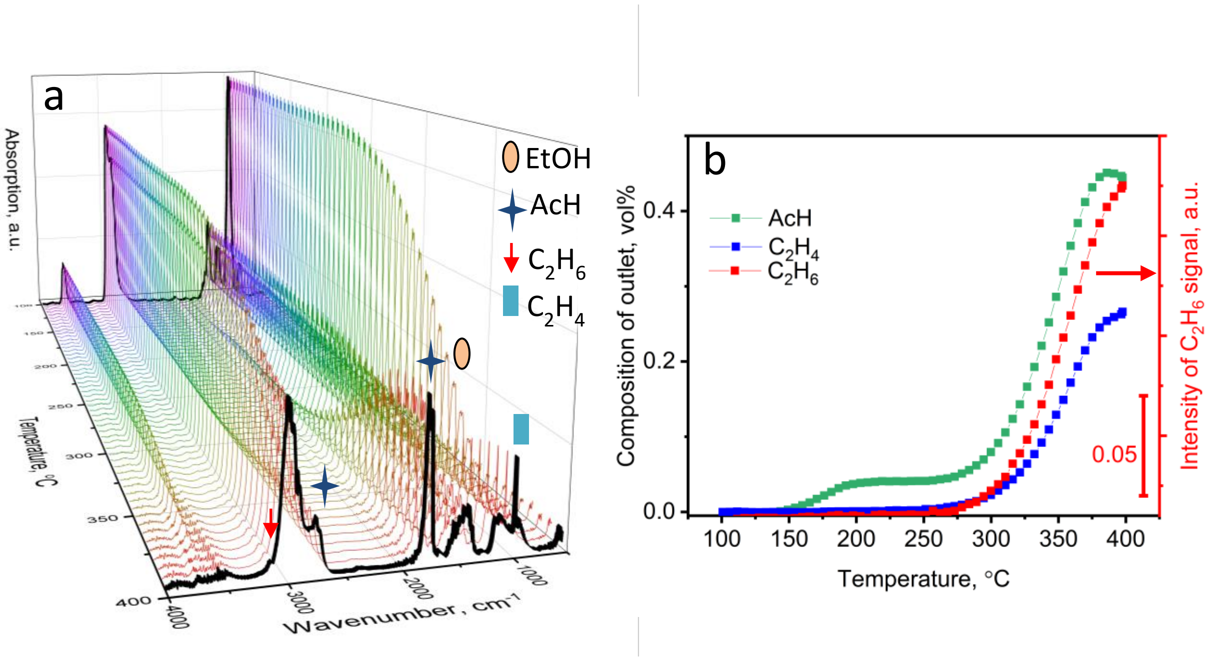

Figure S14. (a) IR spectra at the *operando* reactor outlet during the EtOH TPR experiment. The bands of the products are marked with corresponding symbols. (b) Concentrations of detected products during TPR by ethanol. Concentration of ethane is shown based on the intensity of 3076 cm^{-1} band.

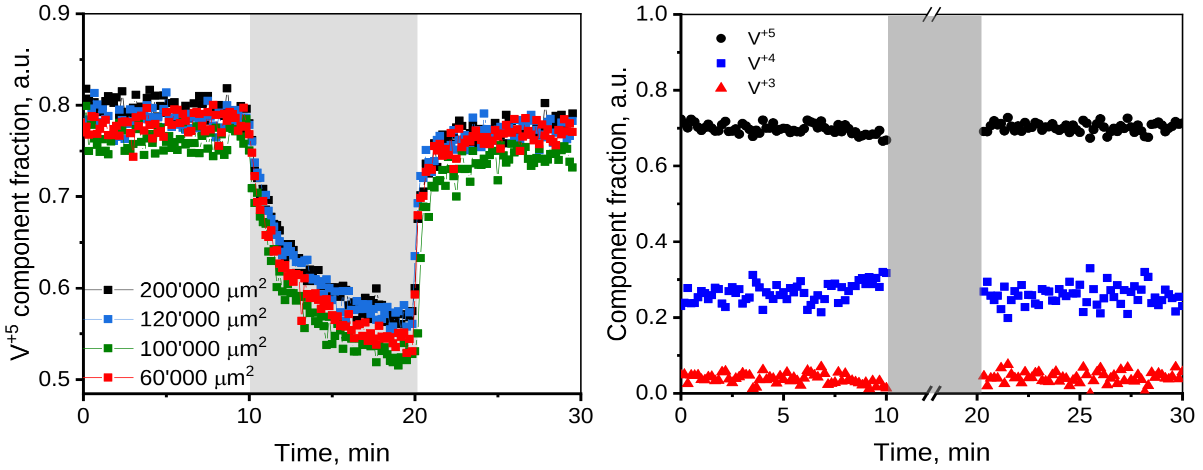

Figure S15. (a) The concentration profiles of V^{5+} during oxygen cut-off experiments with different X-ray beam size: 500 x 400 (200’000) μm^2, 300 x 400 (120’000) μm^2, 500 x 200 (100’000) μm^2, 300 x 200 (60’000) μm^2. (b) The V^{5+}, V^{4+}, V^{3+} concentration profiles during an X-ray beam switching experiment with 500 x 400 μm^2 beam; grey region corresponds to the time interval when the beam was switched off.

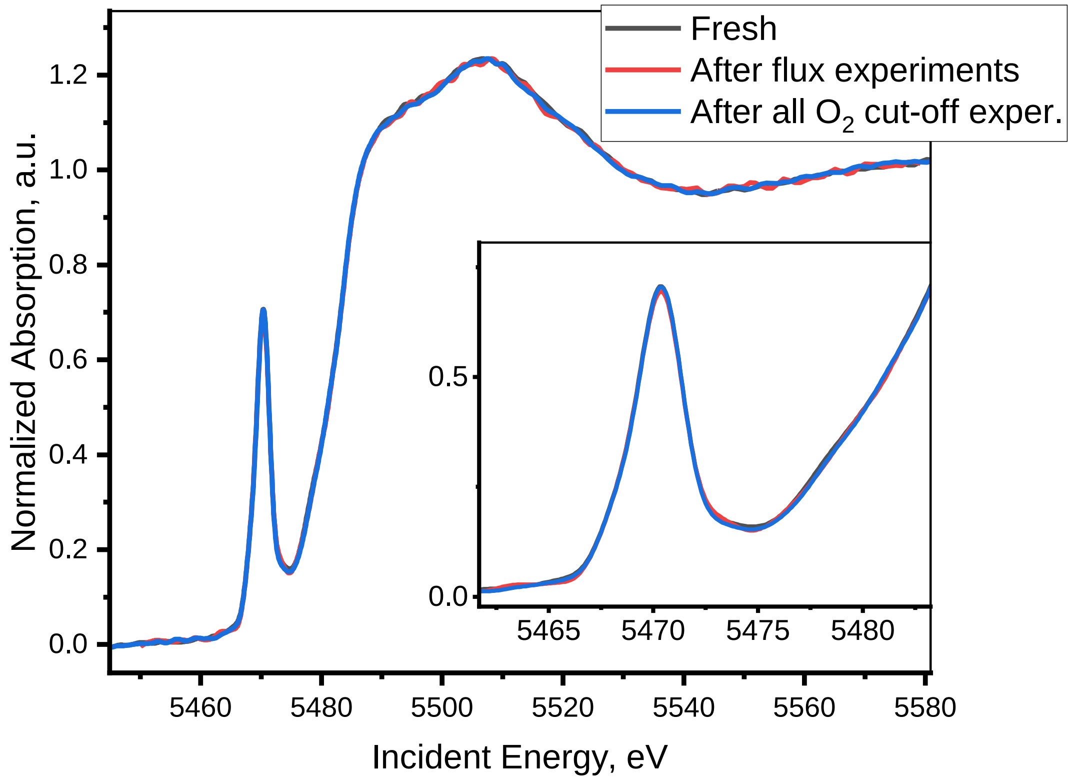

Figure S16. The V K-edge XANES of the 5%V_2O_5/15% TiO_2/SiO_2 catalyst measured on the fresh catalyst (before ethanol exposure), on the catalyst after oxygen cut-off experiments with different flux values (ca. 3 h in EtOH or EtOH+O_2 feed), and after all oxygen cut-off experiments (ca. 24 h in EtOH or EtOH+O_2 feed). All spectra are measured in oxygen-containing feed (6.4 vol% O_2 in He) at 100 or 160 °C after catalyst standard pre-treatment. The inset shows the pre-edge region.

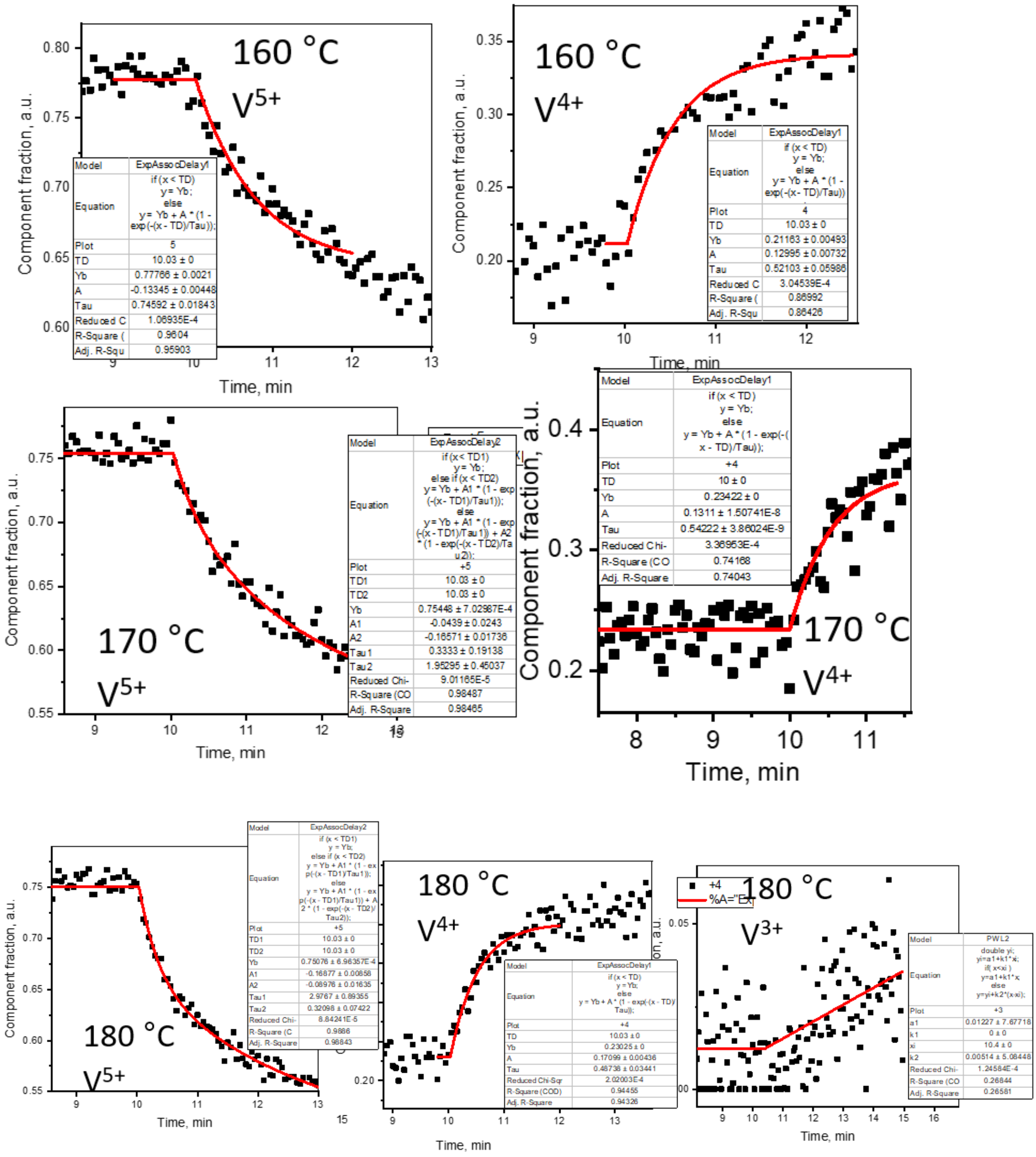

Figure S17. The fitted component concentration profiles at 160, 170, 180 °C upon oxygen switch-off.

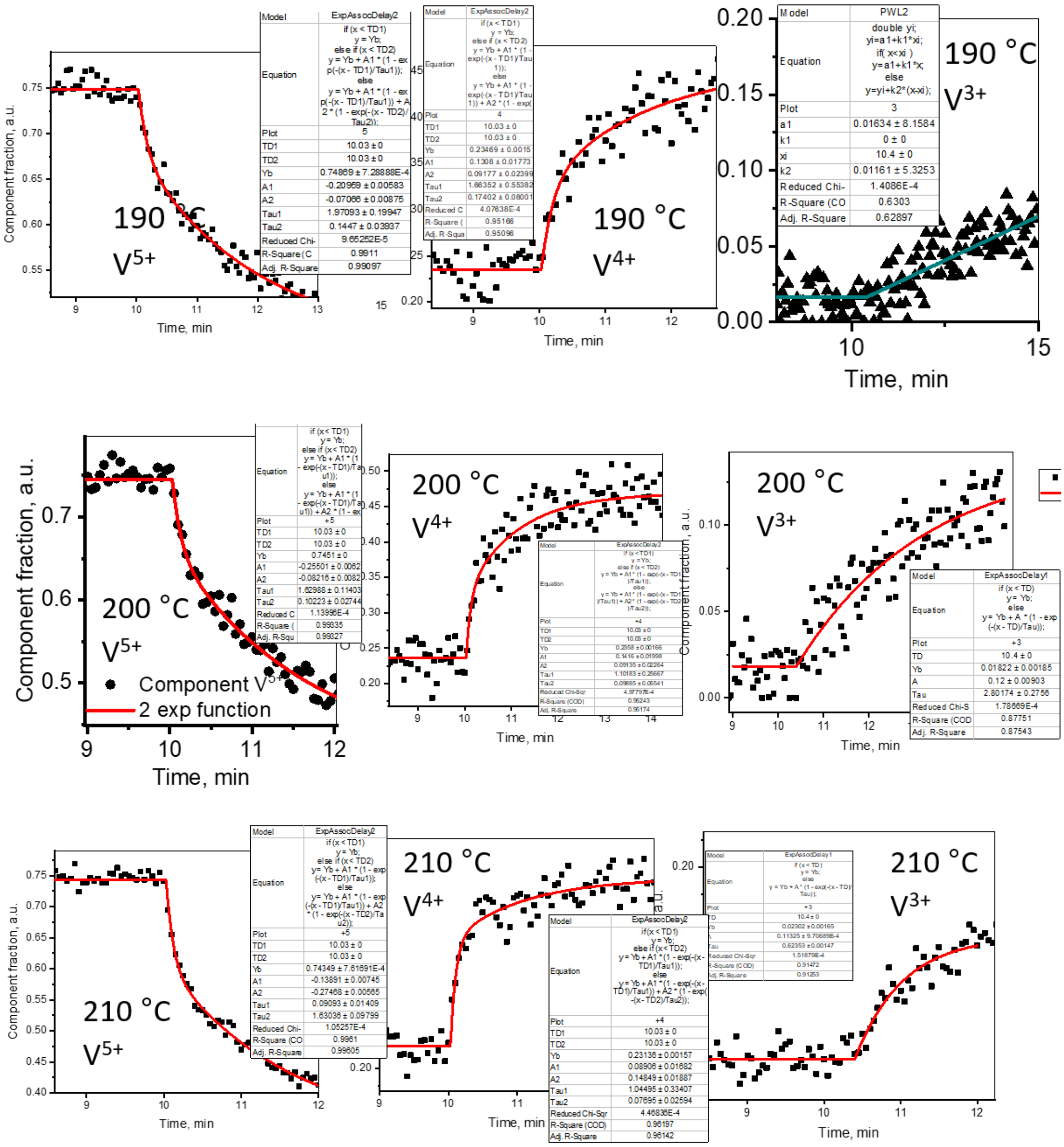

Figure S18. The fitted component concentration profiles at 190, 200, 210 °C upon oxygen switch-off.

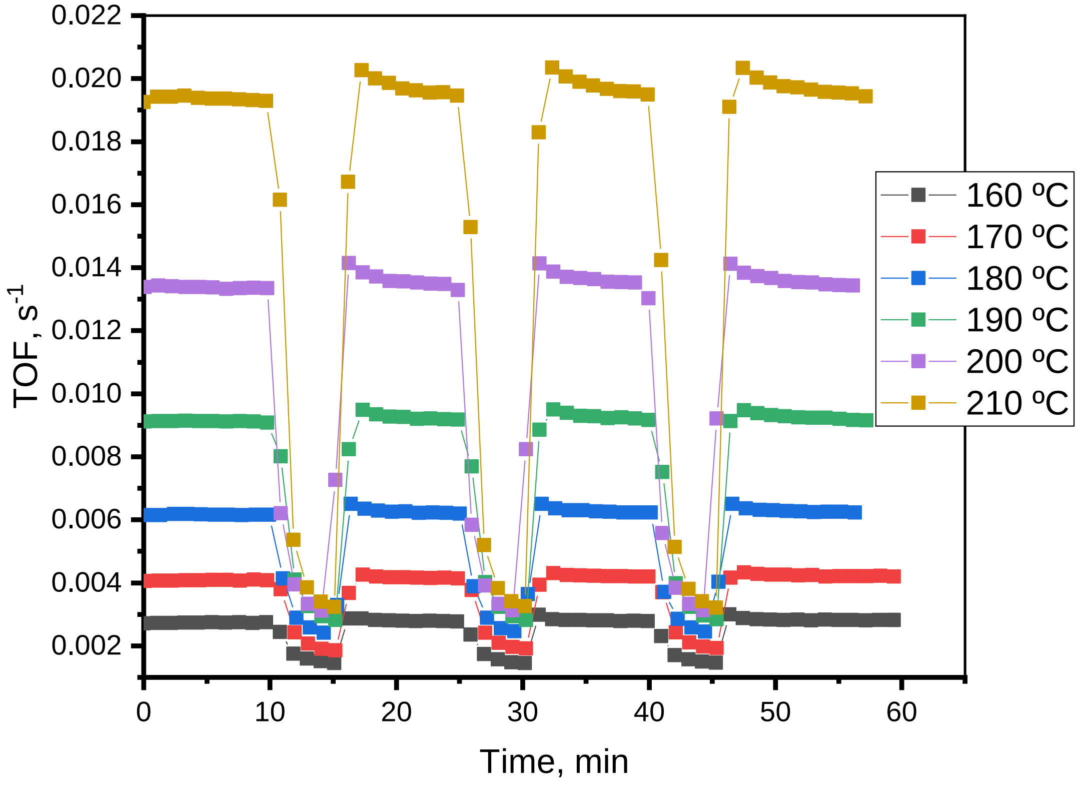

Figure S19. Turn over frequency values measured during *operando* oxygen cut-off experiments over the supported 5 wt% V_2O_5/ 15 wt% TiO_2/SiO_2 catalyst.

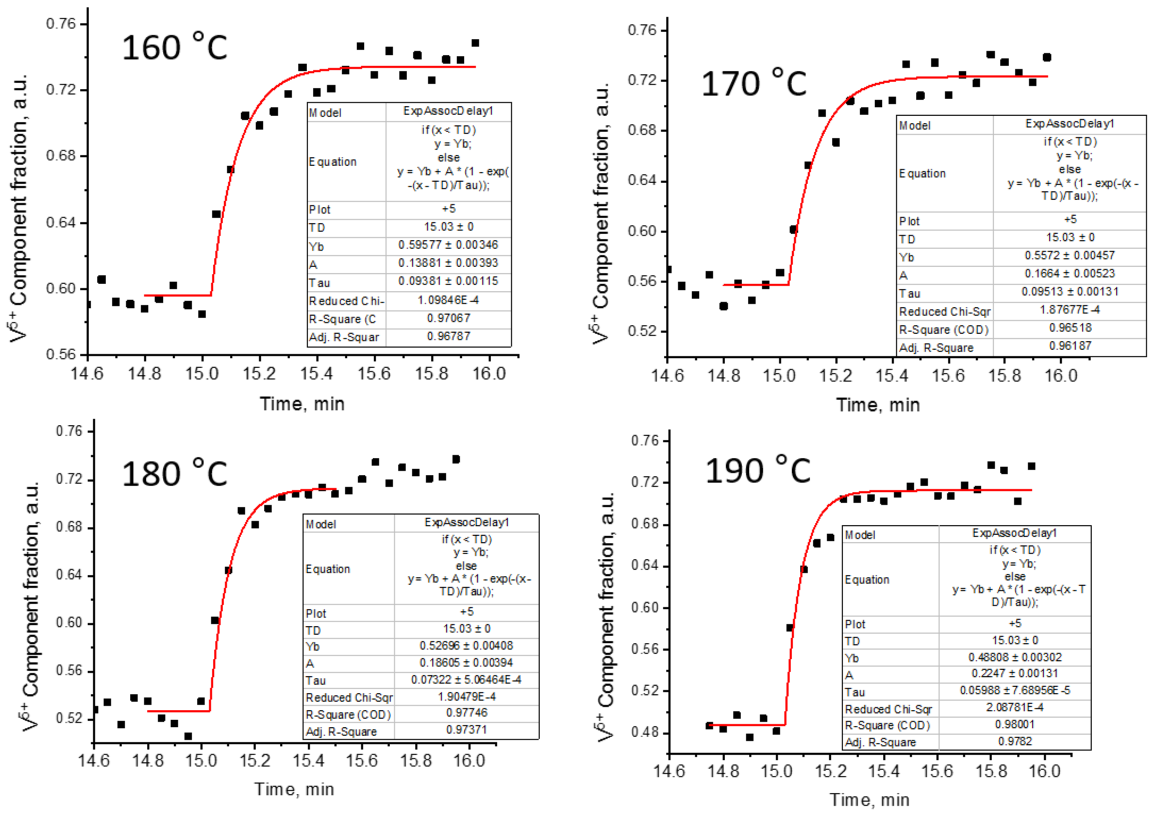

Figure S20. The fitted V^{5+}-component concentration profiles at 190, 200, 210 °C upon oxygen introduction in the oxygen cut-off experiment.

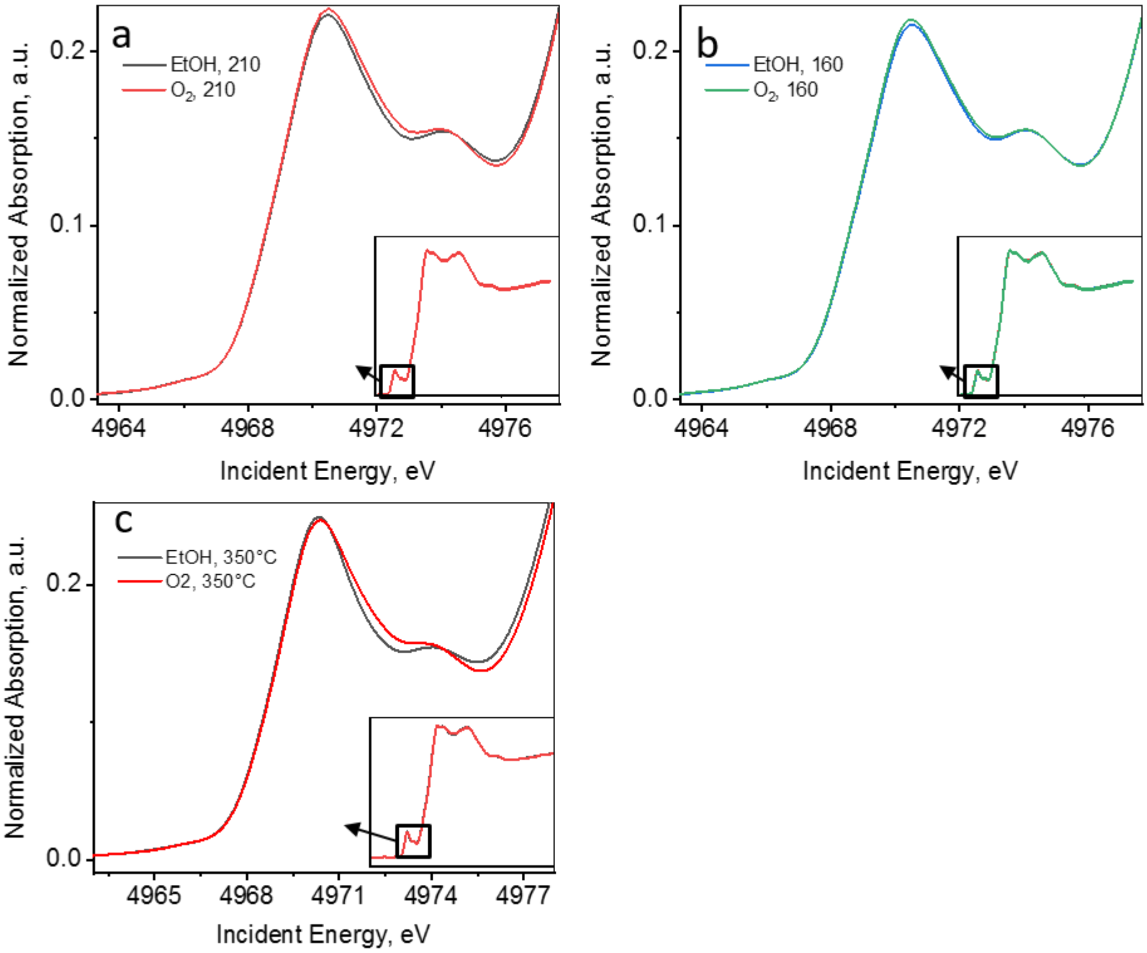

Figure S21. The pre-edge region of Ti K-edge spectra of the catalyst measured in ethanol and in oxygen (a) at 210 °C, (b) at 160 °C and (c) 350 °C. The insets show the full XANES spectra.

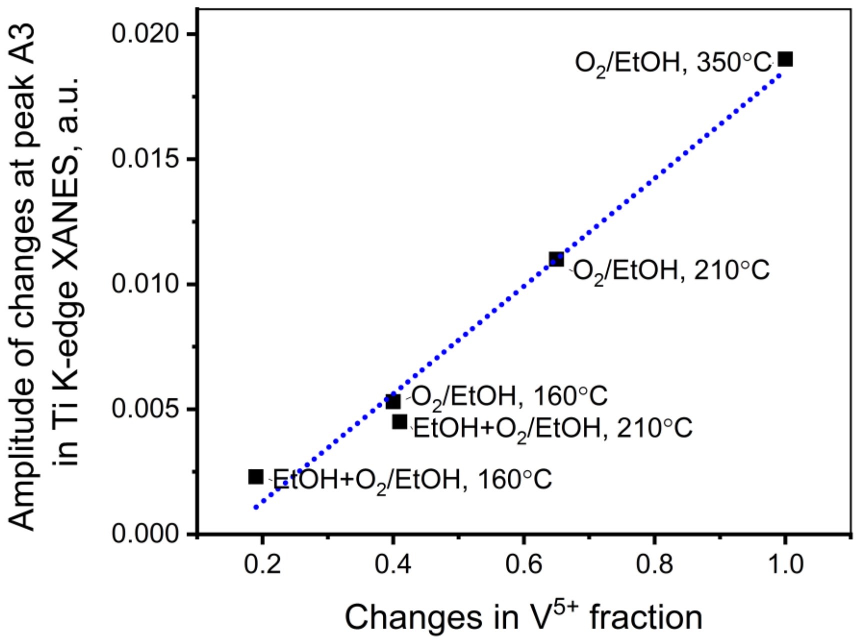

Figure S22. Correlation between changes in V^{5+} fraction and amplitude of A3 peak of Ti K-edge XANES. Details are in Table S3.

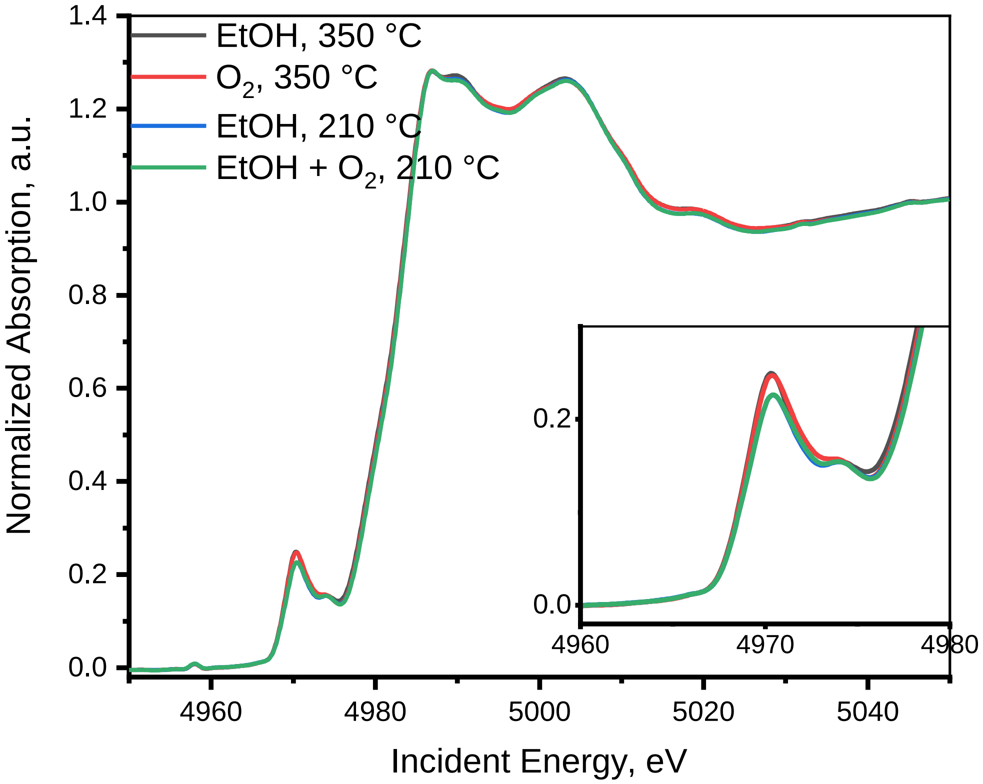

Figure S23. The averaged by phase Ti K-edge spectra of the 5% V_2O_5/15% TiO_2/SiO_2 catalyst measured in EtOH at 350 °C, in O_2 at 350 °C, in EtOH+O_2 at 210 °C, and in EtOH at 210 °C. The inset amplifies the pre-edge region.

3. Supplementary Tables

Table S1. The activity of the $8V_2O_5/SiO_2$, $5\%V_2O_5/TiO_2$ and $5\%V_2O_5$/ x% TiO_2/SiO_2 in ethanol ODH at 200 °C.

	Conversion, %	TOF, s-1	Ea, kJ/mol
8% V_2O_5 / SiO_2*	1	3.3E-4	86
5% V_2O_5/ 1% TiO_2/SiO_2	3	0.0020	78
5% V_2O_5 / 5% TiO_2 / SiO_2	3	0.0010	68
5% V_2O_5 / 8% TiO_2 / SiO_2	17	0.0074	71
5% V_2O_5 / 15% TiO_2 / SiO_2	18	0.013	68
5% V_2O_5 / 25% TiO_2 / SiO_2**	13	0.032	64
5% V_2O_5 / 40% TiO_2 / SiO_2**	22	0.050	65
5% V_2O_5 / 50% TiO_2 / SiO_2**	22	0.053	64
5% V_2O_5 / TiO_2 **	20	0.056	66

*The value of activity is received by extrapolation the activity from high region

**To receive the activity value at 200 °C, the catalyst was diluted 1:3 with SiO_2 (Cab-O-Sil).

Table S2. The functions used to fit the concentration profile. Linear means linear piecewise function; 1 exp is a single exponential function and 2 exp is a double exponential function.

	V^{5+}		V^{4+}		V^{3+}	
T of experiment, °C	Function	Fitting interval, min	Function	Fitting interval, min	Function	Fitting interval, min
160	1 exp	12	1 exp	12.5		
170	2 exp	15	1 exp	11.4		
180	2 exp	15	1 exp	12	linear	15
190	2 exp	15	2 exp	15	linear	15
200	2 exp	15	2 exp	15	linear	15
210	2 exp	15	2 exp	15	1 exp	12

Table S3. Changes in V^{5+} fraction in oxygen switch-off experiment and amplitude of A3 pre-peak in Ti K-edge XANES spectra.

Experiment	V^{5+} fraction in oxidative environment ($EtOH+O_2$ or O_2)	V^{5+} fraction in reductive environment (EtOH)	Changes in V^{5+} fraction upon switching from oxidative to reductive environment	Amplitude of changes at A3 peak in Ti K-edge XANES, a.u.
$EtOH+O_2$/EtOH, 160°C	0.79	0.6	0.19	0.0023
$EtOH+O_2$/EtOH, 210°C	0.76	0.35	0.41	0.0045
O_2/EtOH, 160°C*	1*	0.6*	0.4	0.0053
O_2/EtOH, 210°C*	1*	0.35*	0.65	0.011
O_2/EtOH, 350°C*	1*	0*	1	0.019

* V K-edge was not recorded in EtOH/O_2 experiments. We estimated the V^{5+} fraction as 100% in O_2 environment and equal to $EtOH+O_2$/EtOH values in EtOH environment at identical temperatures. In EtOH/O_2 experiment at 350 °C, we consider that the catalyst is fully oxidized in O_2-feed and fully reduced in EtOH.

4. Supplementary Results and Discussion

4.1. *In situ* DR UV-Vis spectroscopy

In situ UV-vis spectra of 5% V_2O_5/15% TiO_2/SiO_2, 15% TiO_2/SiO_2, and the silica diluent are shown in Figure S24 a. The silica diluent exhibits weak absorbance at <30 000 cm^{-1} and is thus responsible for such absorbance observed for the diluted catalysts, especially at <20 000 cm^{-1} where no such absorbance is expected for $V^{5+}O_x$ and $Ti^{4+}O_x$ species. The line-shape for the 5% V_2O_5/15% TiO_2/SiO_2 catalyst resembles that of the 15% TiO_2/SiO_2 catalyst at >35 000 cm^{-1} and is examined in detail below. The direct bandgaps of the catalysts are determined via Tauc plots in Figure S24 b. The 15% TiO_2/SiO_2 catalyst exhibits a bandgap of 3.6 ± 0.1 eV which

can be assigned to TiO_2 nanocrystals.[19] The 5% V_2O_5/15% TiO_2/SiO_2 catalysts exhibits a lower bandgap of 3.3 ± 0.1 eV, indicating the presence of VO_4 polymers.[20]

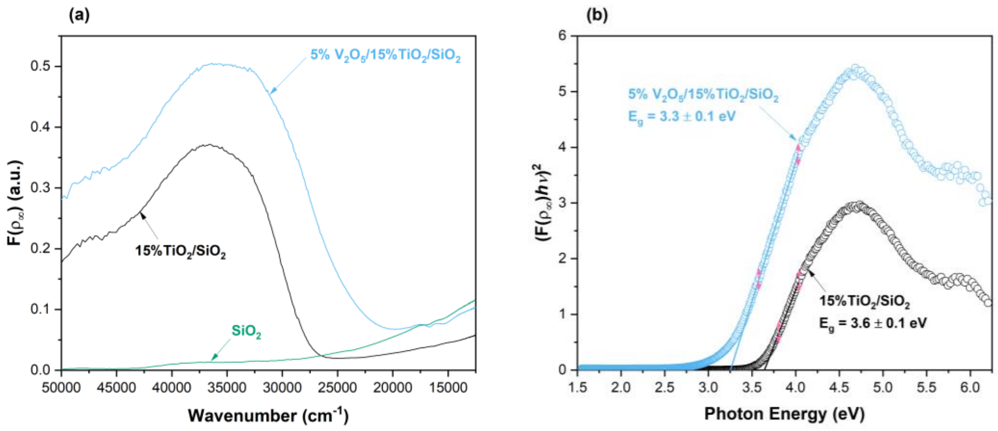

Figure S24 (a) *In situ* DR UV-vis spectra of 5% V_2O_5/15% TiO_2/SiO_2 diluted in SiO_2, 15% TiO_2/SiO_2 diluted in SiO_2, and SiO_2 diluent at 120 °C in 10% O_2/Ar following treatment at 400 °C for 1 h in the same gas mixture. (b) Tauc plots derived from *in situ* spectra of 5% V_2O_5/15% TiO_2/SiO_2 and 15% TiO_2/SiO_2 shown in (a).

The DR UV-vis spectra of the 5% V_2O_5/15% TiO_2/SiO_2 and 15% TiO_2/SiO_2 are further compared in Figure S25 a, where constant-baseline correction and normalization have been applied. It appears that in the region >35 000 cm^{-1} the spectra are nearly identical, suggesting minimal perturbation of TiO_x domains in this spectral region. A difference spectrum is calculated in Figure S25 b. Compared to the 15% TiO_2/SiO_2 catalyst, the 5% V_2O_5/15% TiO_2/SiO_2 catalyst possesses a single additional band at ~28 300 cm^{-1} with an E_g of 3.1 ± 0.1 eV assigned to VO_4 polymers.[20]

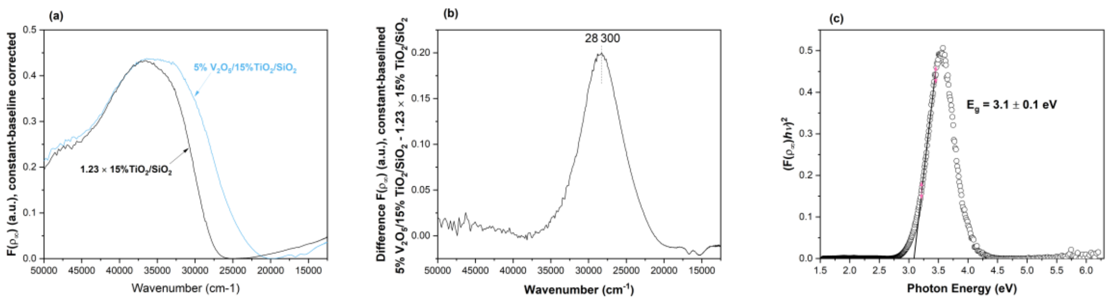

Figure S25 (a) constant-baseline corrected and multiplied *in situ* DR UV-vis spectra of 5% V_2O_5/15% TiO_2/SiO_2 diluted in SiO_2 and 15% TiO_2/SiO_2 diluted in SiO_2 at 120 °C in 10% O_2/Ar following treatment at 400 °C for 1 h in the same gas mixture, where the multiplication

factors are 1.00 and 1.23 for 5% V_2O_5/15% TiO_2/SiO_2 and 15% TiO_2/SiO_2, respectively. (b) Difference spectrum between spectra shown in (a), where 15% TiO_2/SiO_2 is the subtrahend spectrum. (c) Tauc plot derived from difference spectrum shown in (b).

The *in situ* DR UV-vis spectra of 15% TiO_2/SiO_2 and 5% V_2O_5/15% TiO_2/SiO_2 are further analyzed via least-squares fitting in Figure S26 a and Figure S26 c, respectively. An additional band at 35 000 cm^{-1} is present for the 5% V_2O_5/15% TiO_2/SiO_2 sample. Tauc plots that identify the direct band gap of each fitted Gaussian peak are reported in Figure S26 b and Figure S26 d for 15% TiO_2/SiO_2 and 5% V_2O_5/15% TiO_2/SiO_2, respectively. 15% TiO_2/SiO_2 is well-fit by three bands at centered at 46 600, 37 100, and 32 200 cm^{-1} with direct band gaps of 4.8 ± 0.1, 4.0 ± 0.1, and 3.6 ± 0.1 eV, respectively, that are assigned to the $O^{2-} \rightarrow Ti^{4+}$ ligand to metal charge transfer (LMCT) transitions of TiO_4 monomers, TiO_5/TiO_6 polymers, and TiO_2 nanoparticles, respectively.[19] 5% V_2O_5/15% TiO_2/SiO_2 possesses an additional band centered at 30 100 cm^{-1} with a direct band gap of 3.2 ± 0.1 eV, which is assigned to the $O^{2-} \rightarrow V^{5+}$ LMCT transition of VO_4 oligomeric domains.[20] However, the band gap value is larger than 3.0 eV associated with extended oligomeric VO_x domains.[20] Rather, the bandgap value of 3.2 ± 0.1 eV is consistent with one to two V-O-V bonds (within experimental error) based on UV-vis spectra of reference compounds.[21] Therefore, the VO_x domain size on the 5% V_2O_5/15% TiO_2/SiO_2 catalyst is specifically assigned as dimeric or trimeric. The bandgap determined for VO_x dimers/trimers determined via deconvolution is consistent (within experimental error) with that determined when considering the whole spectrum of 5% V_2O_5/15% TiO_2/SiO_2 (Figure S24 b) or when calculating a difference spectrum (Figure S25 c). This consistency corroborates the assignment from the various analytical approaches.

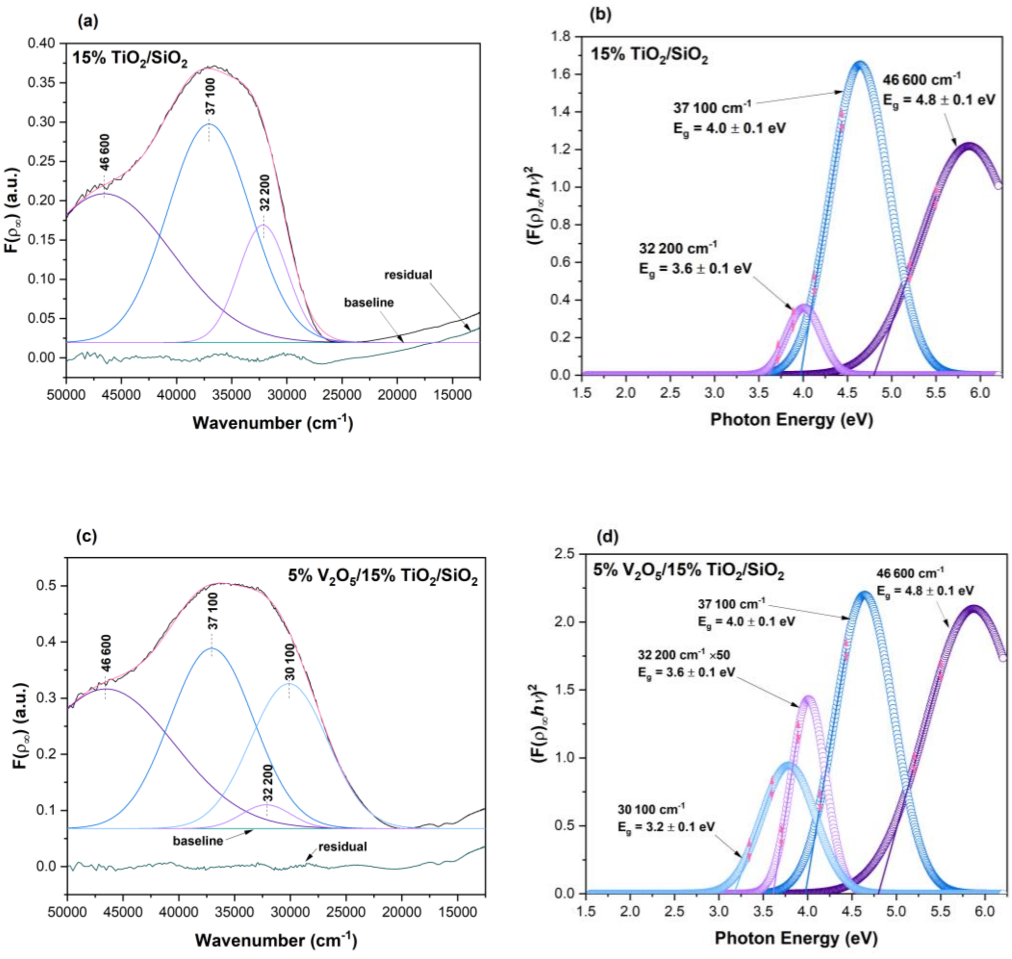

Figure S26 Least-squares fitting of *in situ* DR UV-vis spectra of (a) 5% V_2O_5/15% TiO_2/SiO_2 diluted in SiO_2 and (c) 15% TiO_2/SiO_2 diluted in SiO_2 at 120 °C in 10% O_2/Ar following treatment at 400 °C for 1 h in the same gas mixture. (b) Tauc plot of Gaussian peaks derived from the least-squares fitting of 15% TiO_2/SiO_2 shown in (a). (d) Tauc plot of Gaussian peaks derived from least-squares fitting of 5% V_2O_5/15% TiO_2/SiO_2 shown in (c).

A summary of the results from least-squares fitting of the *in situ* UV-vis spectra of 5% V_2O_5/15% TiO_2/SiO_2 and 15% TiO_2/SiO_2 catalysts is reported inTable S4. Considering the total band area of TiO_x domains, the percentage corresponding to each domain is reported. 15% TiO_2/SiO_2 and 5% V_2O_5/15% TiO_2/SiO_2 possess similar quantities of TiO_4 monomers, TiO_5/TiO_6 polymers, and TiO_2 nanoparticles.

Table S4 Summary of results from least-squares fitting of *in situ* DR UV-vis spectra of 5% V_2O_5/15% TiO_2/SiO_2 and 15% TiO_2/SiO_2 catalysts.

**Band max. (cm^{-1})**	**FWHM (cm^{-1})**	**Direct E_g (eV)**	**Structural Assignment**	**TiO_x band area percentage (%)**		**Ref.**
				15% TiO_2/SiO_2	5% V_2O_5/15% TiO_2/SiO_2	
46 600 ± 900	15 000 ± 3000	4.8 ± 0.1	$Ti^{4+}O_4$ monomer	50 ± 10	60 ± 10	[19]
37 100 ± 100	8 800 ± 700	4.0 ± 0.1	$Ti^{4+}O_5/Ti^{4+}O_6$ oligomer	40 ± 10	40 ± 10	[19]
32 200 ± 100	5 200 ± 200	3.6 ± 0.1	TiO_2 nanoparticles	13 ± 3	3 ± 5	[19]
30 100	8 200	3.2 ± 0.1	$V^{5+}O_4$ dimer/trimer	-	-	[20]

Fang calculated molar extinction coefficients for hydrated TiO_x species supported on mesoporous silica and found that the molar absorption coefficient for TiO_4 monomers is 10-10^2 times that for TiO_x polymer and TiO_2 nanoparticle species,[22] consistent with the finding that the absorption coefficient of cations usually increases by one or two orders of magnitude if the coordination changes from centrosymmetry to non-centrosymmetry.[23,24] The relative quantification of TiO_x species for 15% TiO_2/SiO_2 and 5% V_2O_5/15% TiO_2/SiO_2 catalysts are reported in Table S5. It is found that both catalysts overwhelmingly consist of TiO_5/TiO_6 oligomers, with trace quantities of TiO_4 monomers and TiO_2 nanoparticles, consistent with literature reports for this Ti surface density on the silica support.[25,26]

Table S5 Relative quantification of TiO_x species for 15% TiO_2/SiO_2 and 5% V_2O_5/15% TiO_2/SiO_2 catalysts from *in situ* UV-vis results.

**Species**	**UV-vis molar absorption coefficient ($mmol^{-1}$ cm^2)**[22]	**Concentration of TiO_x species (%)**	
		**15% TiO_2/SiO_2**	**5% V_2O_5/15% TiO_2/SiO_2**
$Ti^{4+}O_4$ monomer	230 × 10^4	1.8 ± 0.6	2.1 ± 0.6
$Ti^{4+}O_5/Ti^{4+}O_6$ polymer	4 × 10^4	90 ± 30	100 ± 30
TiO_2 nanoparticle	17 × 10^4	7.0 ± 2.0	2.0 ± 3.0

4.2. *In situ* Raman spectroscopy

The *in situ* Raman spectra of dehydrated 15% TiO_2/SiO_2 and 5% V_2O_5/15% TiO_2/SiO_2 are presented in Figure S27. For the 15% TiO_2/SiO_2, the bands at 148, 392, 507, and 630 cm^{-1} are assigned to the $E_{g(1)}$, $B_{1g(1)}$, $A_{1g}/B_{1g(2)}$, and $E_{g(3)}$ vibrational modes of crystalline TiO_2 (anatase).[27,28] The blueshift in the peak position of the $E_{g(1)}$ mode relative to the reported value for bulk TiO_2 (anatase) (144 cm^{-1}) is attributed to phonon confinement and indicates the presence of crystallites that are ~6 nm in size.[28] The band at 258 cm^{-1} is tentatively assigned to the δ(Ti-O-Si) bending mode of two-dimensionally dispersed surface TiO_x domains on the SiO_2 support.[19] The bands at 670 and 779 cm^{-1} are assigned to the ν(Ti-OH) and ν(Ti-$(OH)_2$) stretching modes of type I and geminal titanols, respectively.[29,30] The type I titanol has been assigned to TiO_4 monomeric species.[29,30] The band at ~835 cm^{-1} is assigned to a ν(Ti-O-Ti) stretching mode of two-dimensionally dispersed oligomeric TiO_x domains.[31,32] The band at 1067 cm^{-1} is assigned to either a symmetric or asymmetric ν(Ti-O-Si) mode, where the discrimination between two types of vibrational modes is not possible owing to their spectral proximity.[19,29,33] The band at 1164 cm^{-1} is assigned to the asymmetric, longitudinal optical stretching mode of the silica support, $\nu_{as}(Si\text{-}O\text{-}Si)_{LO}$.[34,35] Altogether, the Raman spectrum indicates the presence of TiO_2 (anatase) nanoparticles, monomeric surface TiO_4 species, and oligomeric surface TiO_x species on the silica support. Furthermore, the two-dimensionally dispersed titania species possess hydroxyls.

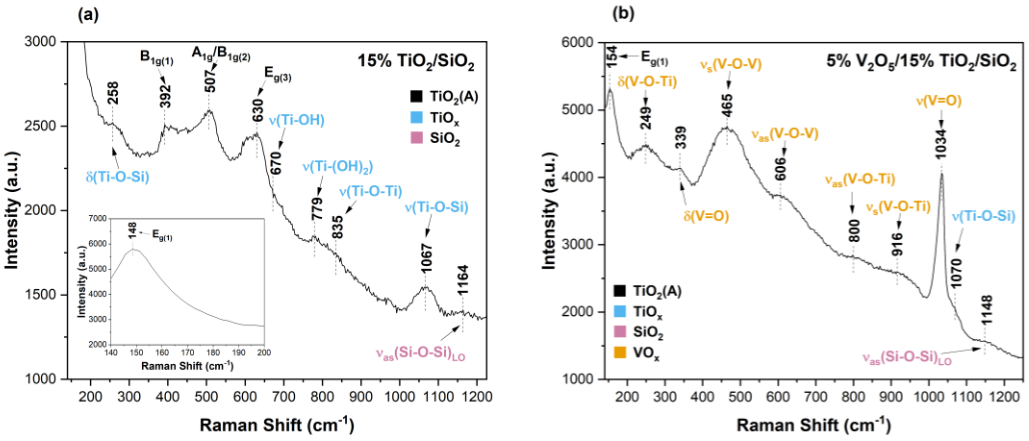

Figure S27. *In situ* Raman spectra of (a) 15% TiO_2/SiO_2 and (b) 5% V_2O_5/15% TiO_2/SiO_2 at 120 °C in 10% O_2/Ar following treatment at 450 °C for 1 h in the same gas mixture.

For the 5% V_2O_5/15% TiO_2/SiO_2 sample, the band at 154 cm^{-1} is assigned to the $E_{g(1)}$ mode of ~4 nm TiO_2 (anatase) nanoparticles.[27,28] The intensity of the $E_{g(1)}$ mode relative to other spectral features is much weaker when compared to the 15% TiO_2/SiO_2 sample, but it is noted that Raman scattering with 442 nm laser excitation will be affected by the optical absorption from VO_x domains and, thus, a quantitative comparison cannot be made with the 15% TiO_2/SiO_2 by Raman spectroscopy. An additional band is observed at 465 cm^{-1} that is suggestive of E_g mode of rutile TiO_2 (reported at 447 cm^{-1} for the bulk).[36] However, the presence of rutile TiO_2 is excluded as its B_{1g} mode is reported at 143 cm^{-1} while the 447 cm^{-1} E_g mode will redshift to lower wavenumbers with either phonon confinement,[36] vanadium doping,[37] or increasing laser power.[38] The presence of V_2O_5 nanoparticles is excluded due to the absence of a band at 996 cm^{-1} assigned the ν(d^1) mode of α-V_2O_5[39,40] as well as the absence of bands at 942 and 1021 cm^{-1} assigned to the ν(V_b-O_{1b}) and ν(V_a-O_{1a}) of β-V_2O_5, respectively.[40] The band at 249 cm^{-1} is assigned to the δ(V-O-Ti) bending mode of V-O-Ti bridging bonds.[26] However, the possibility that δ(V-O-V) vibration also contributes to the 249 cm^{-1} band cannot be ruled out, but such vibrations can couple with the stretching $ν_s$(V-O-V) mode and appear at higher wavenumbers.[40] Compared to the 15% TiO_2/SiO_2 sample, the shift from 258 to 249 cm^{-1} suggests that Ti-O-Si and Ti-O-Ti bonds are not responsible for the observed 249 cm^{-1} band. The band at 339 cm^{-1} is assigned to the δ(V=O) bending mode of the vanadyl moiety.[41] The bands at 465 and 606 cm^{-1} are assigned to the symmetric and asymmetric bending modes of V-O-V bonds, respectively,[41] confirming that VO_x species exist as oligomeric domains. The relatively higher intensity of these modes is tentatively attributed to

resonance enhancement from the 442 nm (22 600 cm^{-1}) laser excitation, which is within the optical absorption envelope of the VO_x domains (see in situ DR UV-Vis discussion). While silica possesses a band at 610 cm^{-1} due to 3-membered ring defects,[34,35] its signal is weaker than for the so-called "R-band" of 4-,5-, and 6-membered rings in the 200-500 cm^{-1} region[34,35] that are not observed. Furthermore, such a band (especially when considering its FWHM) is not clearly observed for the TiO_2/SiO_2 catalyst. It is, therefore, concluded that the 606 cm^{-1} band cannot be attributed to the silica support. The bands at 800 and 916 cm^{-1} are assigned to the asymmetric and symmetric stretching mode of V-O-Ti bonds [42], respectively, confirming that VO_x species anchor on TiO_x domains. The band at 1034 cm^{-1} is assigned to the stretching mode of vanadyl V=O moieties.[26,42–44] The band at 1070 cm^{-1} is assigned to either a symmetric or asymmetric ν(Ti-O-Si) mode, where the discrimination between two types of vibrational modes is not possible owing to their spectral proximity. [19,29,33] It is noted that bands for ν_s(V-O-Si) and ν_{as}(V-O-Si)[33] of VO_x anchored to the silica support are also expected at ~920 cm^{-1} and ~1070 cm^{-1}, respectively.[33,44,45] However, the vanadyl stretch for such silica-supported VO_x domains is observed at 1041 cm^{-1} with 442 nm laser excitation.[45] Thus, the presence of VO_x domains anchored to the silica support is tentatively ruled out. The band at 1148 cm^{-1} is assigned to the asymmetric, longitudinal optical stretching mode of the silica support, $\nu_{as}(Si\text{-}O\text{-}Si)_{LO}$.[34,35] Altogether, the Raman spectrum indicates the presence of two-dimensionally dispersed surface VO_x oligomers anchored to the TiO_x domains.

4.3. Determining the number of components in V K-edge XANES

The Lack of fit of MCR ALS solution received with the use of 2, 3, 4, and 5 pure components is shown in Figure S28. The addition of a third component allows to diminish the lack of fit, whereas the addition of fourth and fifth components does not improve significantly the fitting error. The spectra of components received for 2, 3, and 4-components systems are shown in Figure S29.

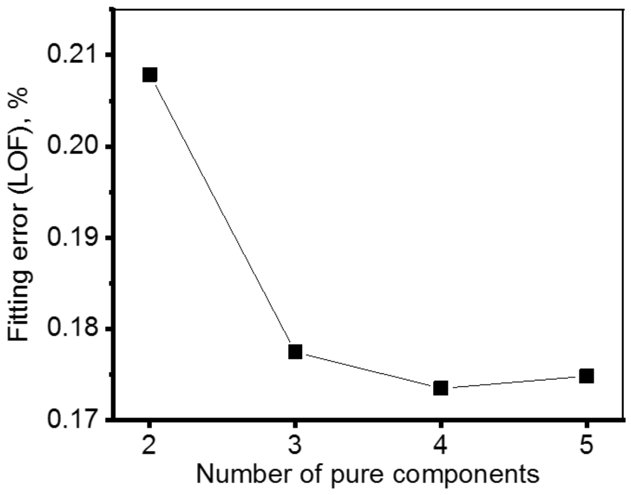

Figure S28. Fitting error as a function of the number of components.

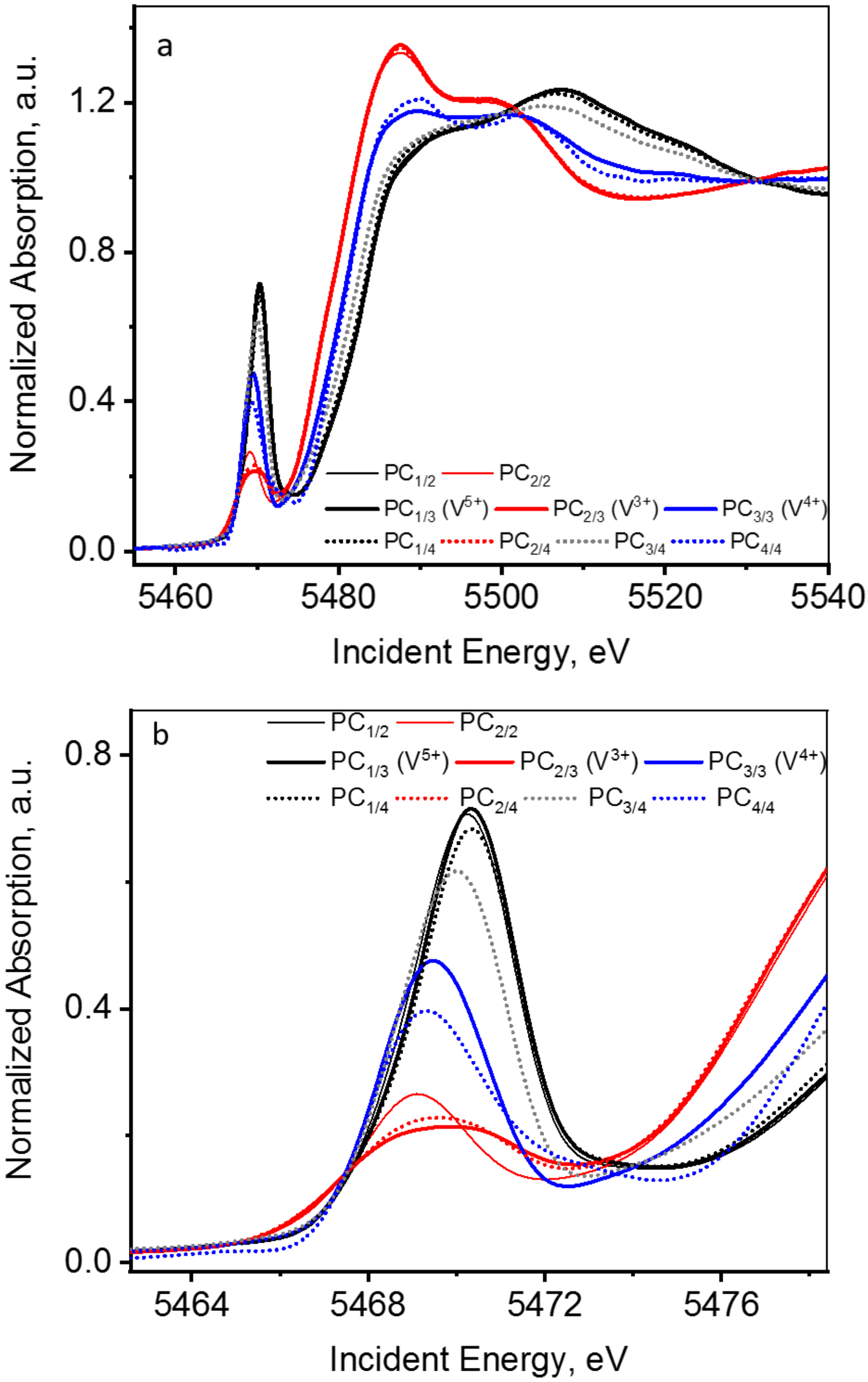

Figure S29. The V K-edge XANES spectra of pure components resolved by MCR-ALS using different number of pure components. The fractions in the name (n/m) in the capture mean the number of pure component (n) in the *m*-component solution; e.g. 2/4 is the second pure component in 4-component solution. The Figure b shows the amplified pre-edge region.

4.4. X-ray Crystallographic Analysis of tailored molecular V-references

The overall molecular structure of $V(OTBOS)_3(thf)_2$ was revealed by X-ray diffraction analysis, though the quality of the diffraction data was low because of a disorder of TBOS ligands, as well as a rotation of coordinating THF; only the connectivity of the molecular structure was clarified. Because of this, a molecular structure of analogous complex, $V(OSi(OMes)(O^tBu)_2)_3(thf)_2$, is shown here instead.

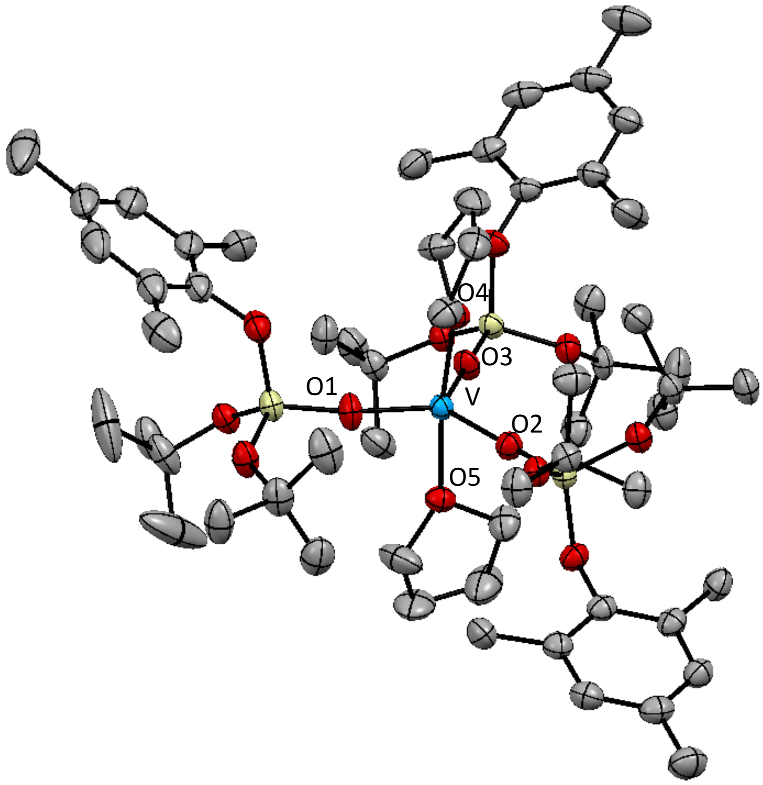

Figure S30. ORTEP drawing of $V(OSi(OMes)(O^tBu)_2)_3(thf)_2$ with ellipsoids at the 50% probability level. All hydrogens are omitted for clarity. Selected bond distances (Å); V–O1, 1.861(2); V–O2, 1.863(2); V–O3, 1.853(2); V–O4, 2.087(2); V–O5, 2.084(9). Each bond length is in an area of that of reported analogous vanadium(III) siloxide complexes.[14]

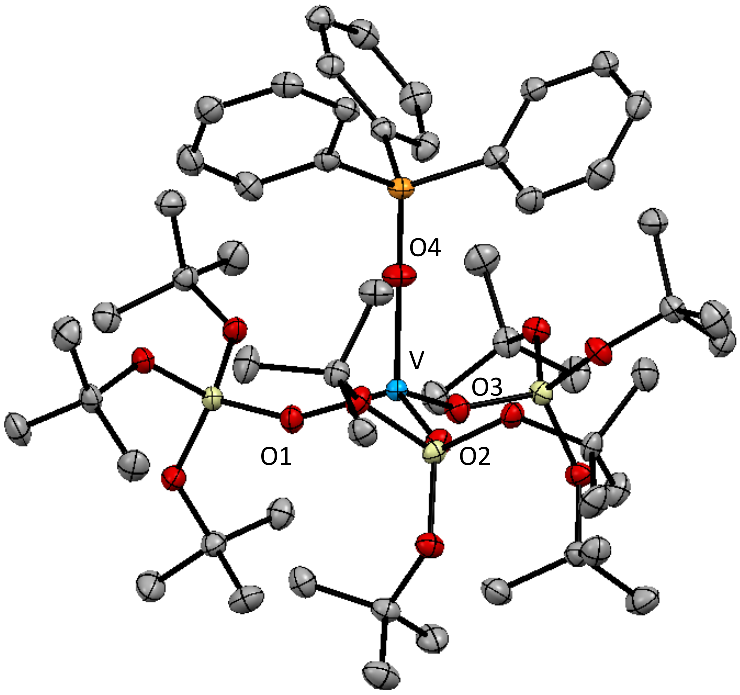

Figure S31. ORTEP drawing of V(OTBOS)$_3$(Ph$_3$PO) with ellipsoids at the 50% probability level. All hydrogens are omitted for clarity. Selected bond distances (Å); V–O1, 1.8499(8); V–O2, 1.8499(8); V–O3, 1.850(8); V–O4, 1.960(15). Each bond length is in an area of that of reported analogous vanadium(III) siloxide complexes.[46]

Table S5. Crystallographic parameters for $V(OSi(OMes)(O^tBu)_2)_3(thf)_2$ and $V(OTBOS)_3(Ph_3PO)$

	$V(OSi(OMes)(O^tBu)_2)_3(thf)_2$	$V(OSi(O^tBu)_3)_3(Ph_3PO)$
empirical formula	$C_{59}H_{103}O_{14}Si_3V$	$C_{54}H_{96}O_{13}PSi_3V$
formula weight	1171.62	1119.52
crystal system	triclinic	Trigonal
space group	$P\bar{1}$	$R\bar{3}$
a, Å	14.0623(6)	21.3914(9)
b, Å	14.1913(6)	21.3914(9)
c, Å	19.8739(9)	23.9645(11)
α, deg.	93.877(2)	90
β, deg.	104.713(2)	90
γ, deg.	118.845(2)	120
V, Å^3	3274.4(3)	9496.8(9)
Z	2	9
D_{calcd}, g/cm^3	1.188	1.174
μ [Mo-Kα], mm^{-1}	0.264	0.293
T, K	100.0	100
crystal size, mm	0.3 × 0.3 × 0.01	0.4 × 0.4 × 0.1
θ range for data collection (deg.)	4.22 to 81.424	4.714 to 56.59
no. of reflections measured	160300	159245
unique data (R_{int})	36742 (0.1229)	5245 (0.0377)
data / restraint / parameters	36742/712/798	5245/0/227
$R1$ ($I > 2.0\sigma(I)$)	0.0747	0.0289
$wR2$ ($I > 2.0\ \sigma(I)$)	0.1654	0.0716
$R1$ (all data)	0.2769	0.0338
$wR2$ (all data)	0.2521	0.0759
GOF on F^2	1.019	1.082
$\Delta\rho$, e Å^{-3}	0.71 / -0.98	0.47 / -0.32

a) $R1 = (\Sigma||Fo| - |Fc||)/(\Sigma |Fo|)$ b) $wR2 = [\{\Sigma\, w(Fo^2-Fc^2)^2\}/\{\Sigma\, w(Fo^4)\}]^{1/2}$

4.5. Assignment of MCR-resolved V K-edge spectra

To assign the MCR resolved V K-edge spectra, we measured V K-edge spectra of 21 V-containing reference compounds and characterized them by calculation the pre-edge height, the pre-edge surface area, the pre-edge center of mass position, and the edge position. In Figure S32 and S33 we built correlation plots using the pre-edge peak area or height and the pre-edge centroid position.[47–49] The MCR-resolved components are included in Figure S32 and S33. The assignment of the oxidation state with such correlations, however, is less reliable than one made using the pre-edge area and the edge position (Figure 4) and does not allow to clearly distinguish neither V^{4+} and V^{5+} nor V^{3+} and V^{4+}.

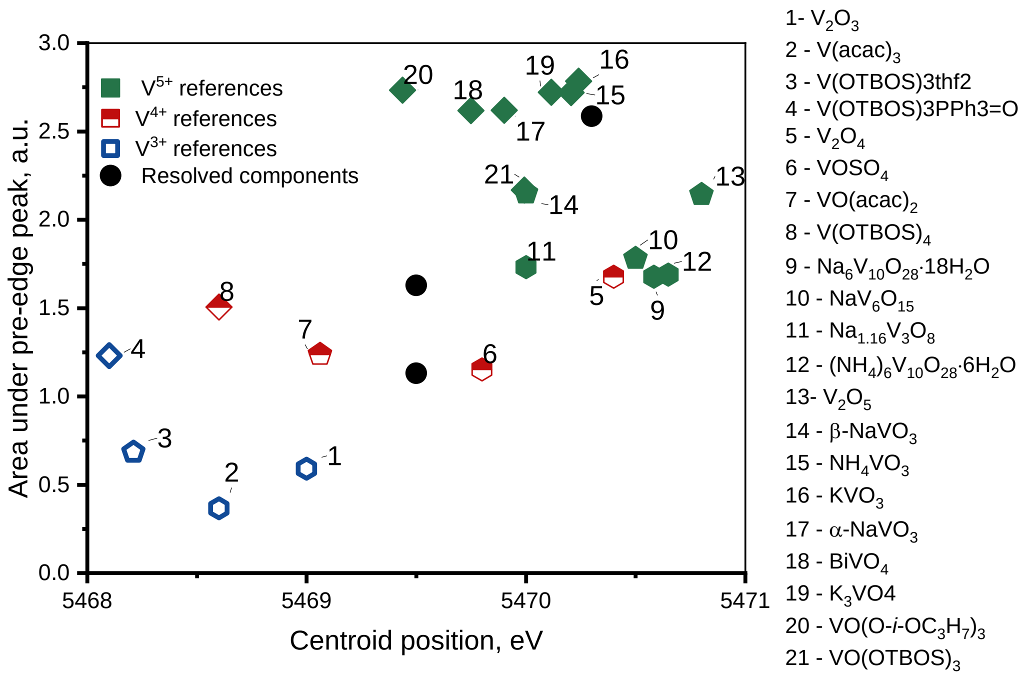

Figure S32. A correlation between the area under the V K pre-edge peak and its centroid position for the listed reference compounds and the MCR-ALS resolved components. Acac corresponds to acetylacetonate ion; OTBOS is OSi(O-*tert*-$C_4H_9)_3$ group.

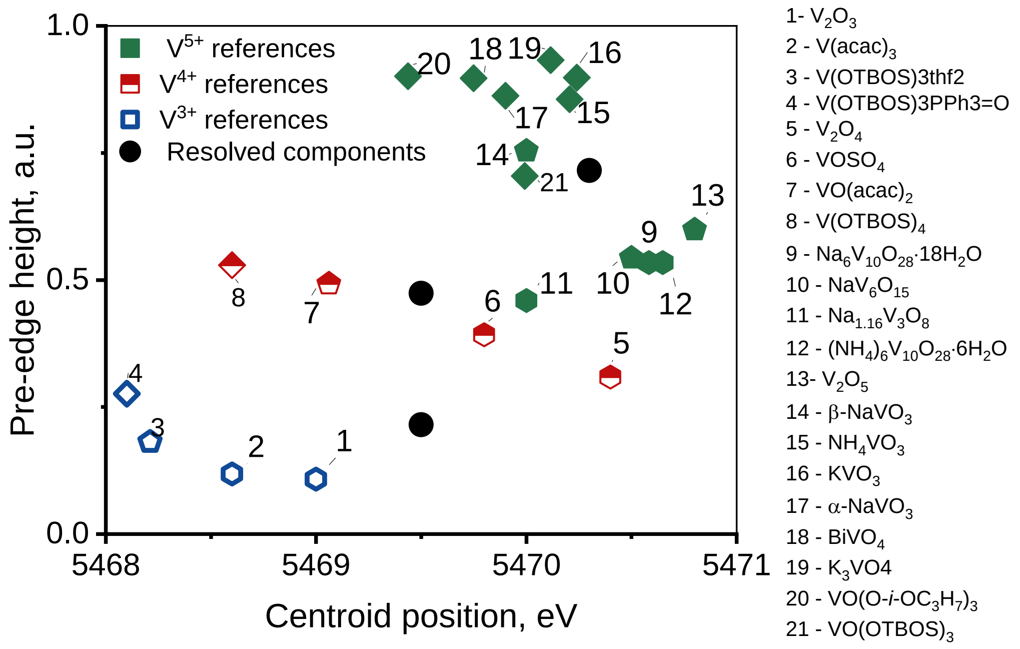

Figure S33. The pre-edge height and its centroid position for the listed reference compounds and the MCR-ALS resolved components. Acac corresponds to acetylacetonate ion; OTBOS is OSi(O-*tert*-$C_4H_9)_3$ group.

In Figures S34-S36 we plotted the V K-edge XANES of the MCR-resolved vanadium components for the 5 wt%V_2O_5/15 wt%TiO_2/SiO_2 catalyst and the best corresponding references. The edge positions of all resolved components coincide with the corresponding references. In the pre-edge region, the MCR resolved V^{5+} component is similar to the 4-fold V^{5+} in α-$NaVO_3$ and 5-fold V^{5+} in β-$NaVO_3$ and V_2O_5 references. This suggests that surface V^{5+} in the investigated catalyst could be present as a mixture of at least two types of V^{5+} moieties, 4- and 5-fold coordinated. The post-edge feature of the V^{5+} component also resembles V^{5+} in the VO(O-*i*-C_3H_7)$_3$ and VO(OTBOS)$_3$ references, which are molecular and/or a liquid and, thus, does not have a long-range order. As for the V^{4+} and V^{3+} components, they possess more intensive pre-edge features than the octahedral references. We associate this with the fact that V^{4+} and V^{3+} species in the catalyst may have a lower coordination number than 6.

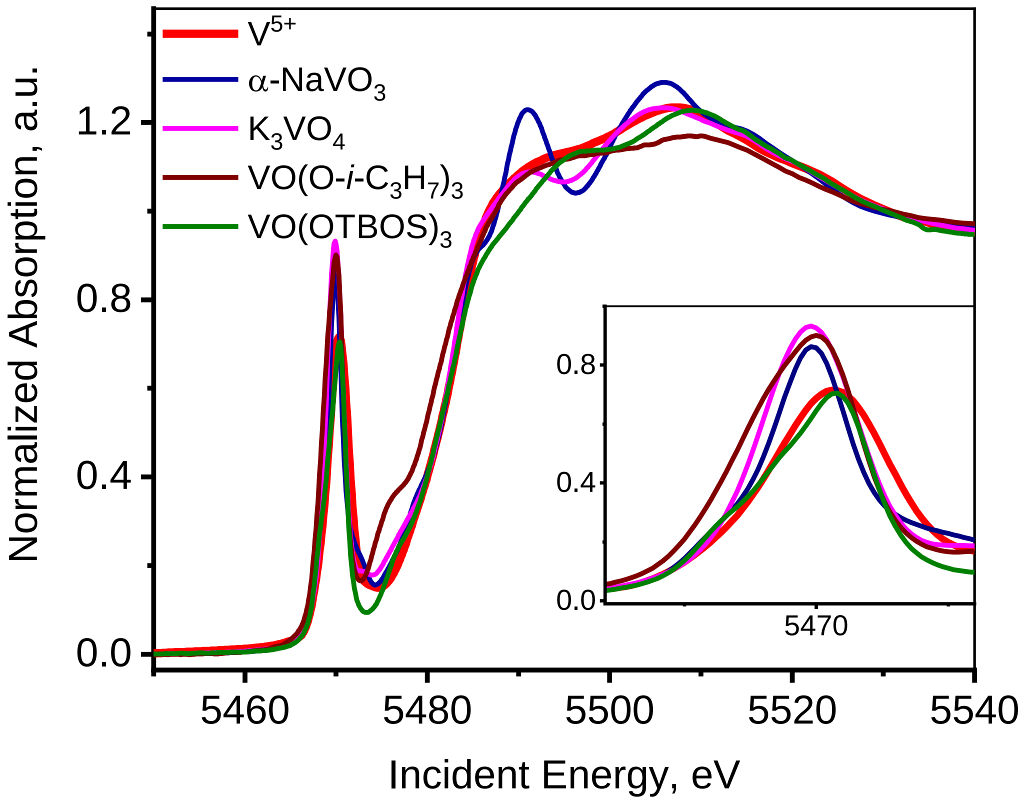

Figure S34 Comparison of the V K-edge XANES spectra of the V^{5+} MCR-ALS resolved component and the most similar V^{5+}-references. The insets zoom on the pre-edge region.

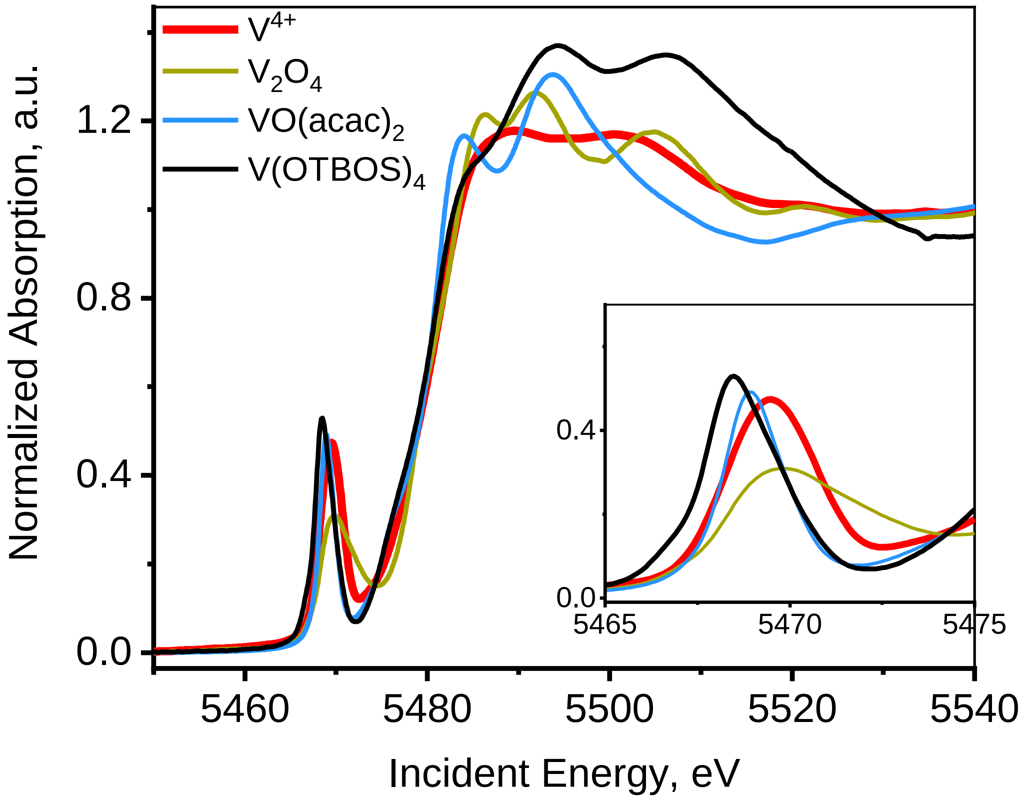

Figure S35 Comparison of the V K-edge XANES spectra of the V^{4+} MCR-ALS resolved component and the V^{4+}-containing references. The insets zoom on the pre-edge region.

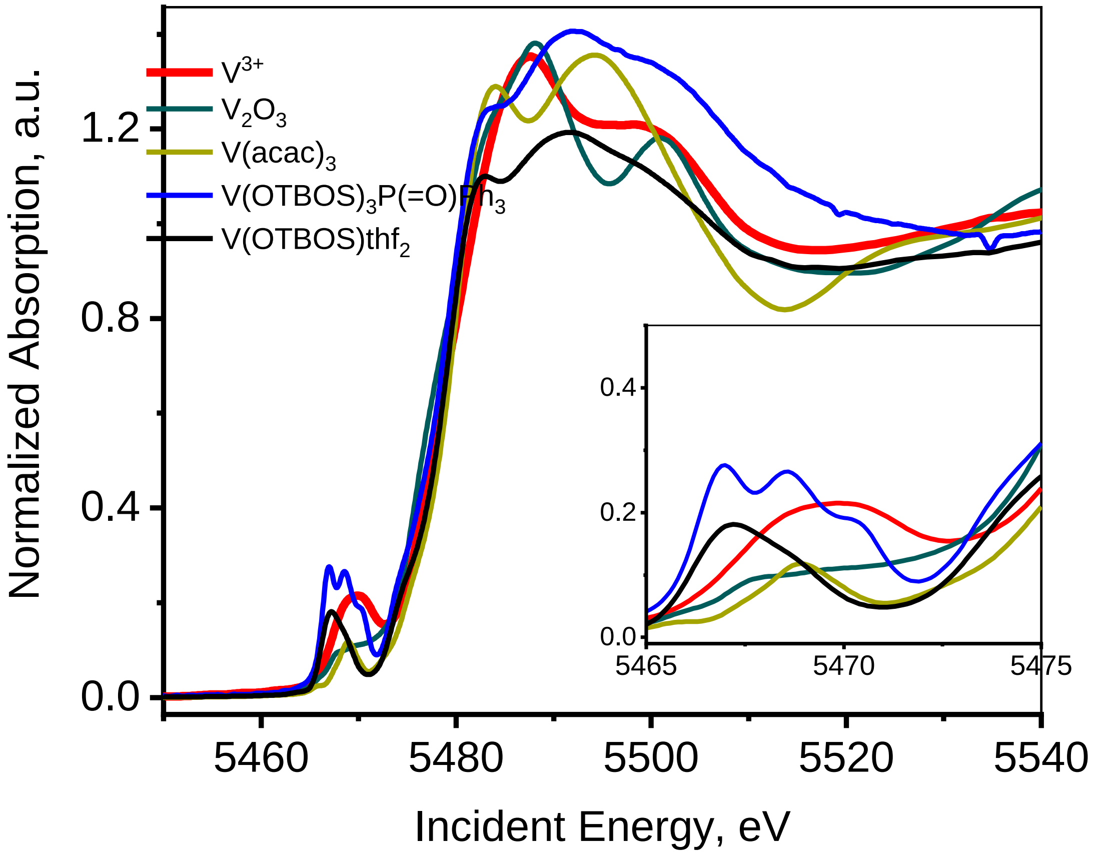

Figure S36. Comparison of the V K-edge XANES spectra of the V^{3+} MCR-ALS resolved component and the V^{3+}-containing references. The insets zoom on the pre-edge region.

4.6. V^{4+} re-oxidation rate

Under steady-state conditions, the rate of V^{5+} reduction and V^{4+} re-oxidation are equal, this can be expressed using the next equation:

$$r_{red} = r_{ox} \text{ or}$$

$$k_{red}[V^{5+}] = k'_{ox}[V^{4+}],$$

Where r_{red} and r_{ox} are the rates of V^{5+} reduction and V^{4+} re-oxidation, respectively; k_{red} is the constant of reduction and k_{ox}' is the constant of re-oxidation (includes the oxygen concentration).

Thus, under steady-state conditions, the ratio between V^{5+} and V^{4+}-species is equal to:

$$\frac{[V^{5+}]}{[V^{4+}]} = \frac{k'_{ox}}{k_{red}}$$

Which was estimated experimentally using the next equation:

$$\frac{[V^{5+}]}{[V^{4+}]} = \frac{\frac{r_{ox}}{[V^{4+}]_{t=15}}}{\frac{r_{red}}{[V^{5+}]_{t=10}}}$$

Where $[V^{4+}]_{t=15}$ and $[V^{5+}]_{t=10}$ are the concentrations of V^{4+} and V^{5+} when oxygen was switched off (t=10 min) and on (t= 15 min), respectively; r_{red} and r_{ox} – the rates of V^{5+} consumption (upon oxygen switch-off) and appearance (upon oxygen switch-on) in oxygen cut-off experiments, respectively.

The fits of V^{5+} formation upon oxygen switch on are shown in Figure S20.

As we discussed in the main text, due to oxygen leak, acetaldehyde concentration never reached 0. As a result, a fraction of V-species was involved in the MvK cycles also in the "oxygen-free" ethanol-containing phase and was not detected among reduced species. This means that the observed rate of V^{4+} production is underestimated by 2*r_{AcH} (r_{AcH} – observed in oxygen switch off phase rate of acetaldehyde production, 2 mol of V^{4+} are produced per 1 mol of acetaldehyde). We took it in an account, and in Table S5, we show the expected V^{4+} concentration with the correction to the O_2-leak through the graphite window.

Table S5. The rates and ratios of $V^{5+} \rightarrow V^{4+}$ and $V^{4+} \rightarrow V^{5+}$ processes in oxygen cut-off experiments.

T, °C	Rate of reduction $V^{5+} \rightarrow V^{4+}$, mol/min/$g_{cat}$	Rate of re-oxidation $V^{4+} \rightarrow V^{5+}$, mol/min/$g_{cat}$	Ratio of re-oxidation and reduction rates	Fraction of V^{5+} upon oxygen switch-off	Fraction of V^{4+} upon oxygen switch-on	Expected V^{4+} fraction under steady-state conditions	Expected V^{4+} with correction to O_2-leak	Observed V^{4+} fraction under steady-state conditions
160	9.83E-05	8.13E-04	8.3	0.78	0.4	0.06	0.10	0.22
170	1.19E-04	9.61E-04	8.1	0.75	0.42	0.07	0.10	0.25
180	1.85E-04	1.40E-03	7.6	0.75	0.45	0.08	0.12	0.25
190	3.27E-04	2.06E-03	6.3	0.75	0.45	0.10	0.10	0.25

4.7. Activity of titanium during alcohol oxidation

In the first type of *operando* ME Ti K-edge XAS experiments, the ethanol concentration in the feed was held constant, and the flow of oxygen was periodically switched on and off every 5 min (denoted as EtOH+O_2→EtOH cycling). In the second type of the ME Ti K-edge XAS experiments, the oxygen and ethanol flows were periodically (every 5 min) alternated (denoted as O_2→EtOH cycling). The latter experiments represent the most extreme conditions of ODH, reminiscent of a chemical looping operation. After recording 10 cycles at each working temperature, the resulting Ti K-edge XAS spectra were normalized and analyzed using the PSD approach in the XANES region. The phase-resolved spectra from the ME XAS experiments over the supported 5% V_2O_5/15% TiO_2/SiO_2 catalyst are shown in Figure S37 together with the Ti K-edge XANES spectrum of the same catalyst measured at 350 °C in an oxygen flow.

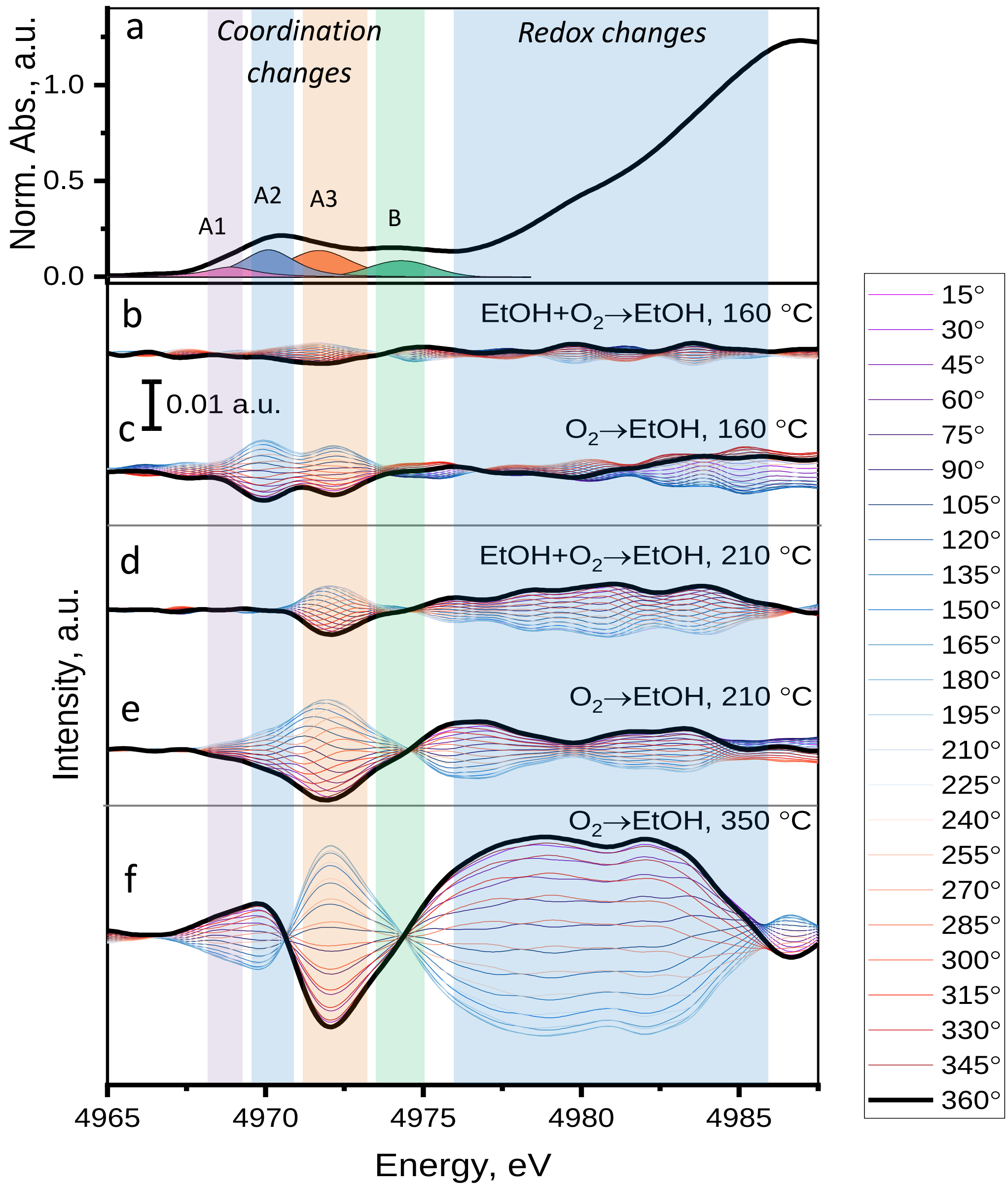

Figure S37. (a) Ti K-edge XANES spectrum (20 vol% O_2, 400 °C) of the 5% V_2O_5/15% TiO_2/SiO_2 catalyst. (b and d) The phase-resolved spectra from the ME Ti K-edge XAS experiments during the periodic switching off oxygen from the ethanol-oxygen feed at 160 and 210 °C, respectively. (c, e, and f) The phase-resolved spectra from the ME Ti K-edge XAS experiments during the periodic switching between oxygen- and ethanol-containing feeds at 160, 210 and 350 °C, respectively. Bold curves in (b-f) represent spectral changes when switching from more oxidizing (O_2 or EtOH+O_2) to more reducing (EtOH) feed. The colored intervals show the changes in theA1, A2, A3, and B features in the pre-edge region (4968-4975 eV) and in the edge region (4977 - 4986 eV).

Comparison of the phase-resolved Ti K-edge spectra in the pre-edge region (4968-4970 eV) (Figure S37) revealed interesting details. When ethanol is constantly present in the feed (Figure S37 b, d), no significant changes in the pre-edge peaks A1 and A2 are detected. However, small changes in the peak intensities of A1 and A2 can be observed if ethanol is periodically removed from the feed (during O_2 / EtOH experiments, Figure S37 c, e, f). These peaks have mostly local character; changes in A2 intensity indicate changes in the symmetry or the coordination number of titanium.[50] At 350 °C, A1 and A2 peaks are more intense in the presence of ethanol (the bold curve in Figure S37 f representing EtOH to oxygen switch has positive values; also see Figure S21 c) indicating that the titanium coordination number decreases in the reducing environment. This could be an evidence of oxygen-vacancy formation upon partial titanium reduction.

The O_2/EtOH ME Ti K-edge XAS experiments performed at lower temperatures (160 and 210 °C, Figure S37 c, e and S21) demonstrate the reverse character of the A1 and A2 changes: the bold curves (O_2 to EtOH switch) are negative in the A1 and A2 regions meaning that the A1 and A2 peaks are less intense in ethanol. This suggests that titanium has more neighbors in the first coordination sphere in EtOH and its coordination structure is closer to a perfect octahedron. Presumably, when ethanol is present in the feed, it can adsorb at the 5-fold coordinated titanium sites leading to an increase in the coordination number of Ti.[51] Apparently, at lower temperatures (160 and 210 °C) this effect is greater than oxygen-vacancy formation due to titanium reduction. However, the intensities of these changes are extremely small indicating that only a small fraction of titanium atoms undergo any transformation (Figures S21, S23).

5. Supplementary References

(1) Bulánek, R.; Čapek, L.; Setnička, M.; Čičmanec, P. DR UV-Vis Study of the Supported Vanadium Oxide Catalysts. *J. Phys. Chem. C* **2011**, *115* (25), 12430–12438. https://doi.org/10.1021/jp112206c.

(2) KUBELKA, P. New Contributions to the Optics of Intensely Light-Scattering Materials. *J. Opt. Soc. Am.* **1948**, *38* (5), 448–457. https://doi.org/10.1364/JOSA.38.000448.

(3) Wojdyr, M. Fityk: A General-Purpose Peak Fitting Program. *J. Appl. Crystallogr.* **2010**, *43*, 1126–1128. https://doi.org/10.1107/S0021889810030499.

(4) Clark, A. H.; Imbao, J.; Frahm, R.; Nachtegaal, M. ProQEXAFS: A Highly Optimized Parallelized Rapid Processing Software for QEXAFS Data. *J. Synchrotron Radiat.* **2020**, *27*, 551–557. https://doi.org/10.1107/S1600577519017053.

(5) Müller, P.; Hermans, I. Applications of Modulation Excitation Spectroscopy in Heterogeneous Catalysis. *Ind. Eng. Chem. Res.* **2017**, *56* (5), 1123–1136. https://doi.org/10.1021/acs.iecr.6b04855.

(6) Dolomanov, O. V.; Bourhis, L. J.; Gildea, R. J.; Howard, J. A. K.; Puschmann, H. OLEX2: A Complete Structure Solution, Refinement and Analysis Program. *J. Appl. Crystallogr.* **2009**, *42* (2), 339–341. https://doi.org/10.1107/S0021889808042726.

(7) Palatinus, L.; Van Der Lee, A. Symmetry Determination Following Structure Solution in P1. *J. Appl. Crystallogr.* **2008**, *41* (6), 975–984. https://doi.org/10.1107/S0021889808028185.

(8) Palatinus, L.; Chapuis, G. SUPERFLIP - A Computer Program for the Solution of Crystal Structures by Charge Flipping in Arbitrary Dimensions. *J. Appl. Crystallogr.* **2007**, *40* (4), 786–790. https://doi.org/10.1107/S0021889807029238.

(9) Sheldrick, G. M. Crystal Structure Refinement with SHELXL. *Acta Crystallogr. Sect. C Struct. Chem.* **2015**, *71* (Md), 3–8. https://doi.org/10.1107/S2053229614024218.

(10) Jaumot, J.; de Juan, A.; Tauler, R. MCR-ALS GUI 2.0: New Features and Applications. *Chemom. Intell. Lab. Syst.* **2015**, *140*, 1–12. https://doi.org/10.1016/j.chemolab.2014.10.003.

(11) Nadjafi, M.; Abdala, P. M.; Verel, R.; Hosseini, D.; Safonova, O. V.; Fedorov, A.; Müller, C. R. Reducibility and Dispersion Influence the Activity in Silica-Supported Vanadium-Based Catalysts for the Oxidative Dehydrogenation of Propane: The Case of Sodium Decavanadate. *ACS Catal.* **2020**, *10* (3), 2314–2321. https://doi.org/10.1021/acscatal.9b04752.

(12) Johnson, G. K.; Murmann, R. K.; Deavin, R.; Griffith, W. P. Sodium and Ammonium Decayanadates(V). In *Inorganic Syntheses, Volume 19*; Shriver, D. F., Ed.; John Wiley & Sons, Inc, 1979; pp 140–145. https://doi.org/10.1002/9780470132500.ch32.

(13) Rulkens, R.; Male, J. L.; Terry, K. W.; Olthof, B.; Khodakov, A.; Bell, A. T.; Iglesia, E.; Don Tilley, T. Vanadyl Tert-Butoxy Orthosilicate, OV[OSi(OtBu)3]3: A Model for Isolated Vanadyl Sites on Silica and a Precursor to Vanadia-Silica Xerogels A. *Chem. Mater.* **1999**, *11* (10), 2966–2973. https://doi.org/10.1021/cm990350o.

(14) Rost, M.; Görls, H.; Imhof, W.; Seidel, W.; Thiele, K. Synthese Und Struktur von Vanadium(III)-Und Vanadium(IV)-Silanolaten. *Zeitschrift für Anorg. und Allg. Chemie* **1998**, *624* (12), 1994–

2000. https://doi.org/10.1002/(SICI)1521-3749(1998120)624:12<1994::AID-ZAAC1994>3.0.CO;2-R.

(15) Smith, P. D.; Huffman, J. C.; Bansemer, R. L.; Martin, J. L.; Caulton, K. G. Bimetallic Halides. Crystal Structure of and Ethylene Polymerization by VCl2ZnCl2-4THF. *Inorg. Chem.* **1985**, *24* (19), 2997–3002. https://doi.org/10.1021/ic00213a025.

(16) Hitchcock, P. B.; Hughes, D. L.; Leigh, G. J.; Sanders, J. R.; De Souza, J.; McGarry, C. J.; Larkworthy, L. F. Preparation of New Vanadium(II) Iodides and Crystal Structure of Hexakis(Acetonitrile)Vanadium(II)(Tetraiodide). *J. Chem. Soc. Dalt. Trans.* **1994**, No. 24, 3683–3687. https://doi.org/10.1039/dt9940003683.

(17) McMullen, A. K.; Tilley, T. D.; Rheingold, A. L.; Geib, S. J. Preparation and Characterization of the Monomeric Copper(II) Siloxide Complex Cu[OSi(OCMe3)3]2(Py)2. *Inorg. Chem.* **1989**, *28* (19), 3772–3774. https://doi.org/10.1021/ic00318a032.

(18) Docherty, S. R.; Estes, D. P.; Copéret, C. Facile Synthesis of Unsymmetrical Trialkoxysilanols: (RO)2(R'O)SiOH. *Helv. Chim. Acta* **2018**, *101* (3). https://doi.org/10.1002/hlca.201700298.

(19) Gao, X.; Bare, S. R.; Fierro, J. L. G.; Banares, M. A.; Wachs, I. E. Preparation and In-Situ Spectroscopic Characterization of Molecularly Dispersed Titanium Oxide on Silica. *J. Phys. Chem. B* **1998**, *102* (29), 5653–5666. https://doi.org/10.1021/jp981423e.

(20) Tian, H.; Ross, E. I.; Wachs, I. E. Quantitative Determination of the Speciation of Surface Vanadium Oxides and Their Catalytic Activity. *J. Phys. Chem. B* **2006**, *110* (19), 9593–9600. https://doi.org/10.1021/jp055767y.

(21) Gao, X.; Wachs, I. E. Investigation of Surface Structures of Supported Vanadium Oxide Catalysts by UV-Vis-NIR Diffuse Reflectance Spectroscopy. *J. Phys. Chem. B* **2000**, *104* (6), 1261–1268. https://doi.org/10.1021/jp992867t.

(22) Fang, L. Surface Engineering of Mesoporous Silica for Ti-Based Epoxidation Catalysts, Ecole normale supérieure de lyon - ENS LYON; East China normal university (Shanghai), 2012.

(23) Klaas, J.; Schulz-Ekloff, G.; Jaeger, N. I. UV-Visible Diffuse Reflectance Spectroscopy of Zeolite-Hosted Mononuclear Titanium Oxide Species. *J. Phys. Chem. B* **1997**, *101* (8), 1305–1311. https://doi.org/10.1021/jp9627133.

(24) Burdett, J. K.; Hughbanks, T. Aspects of Metal-Metal Bonding in Early-Transition-Metal Dioxides. *Inorg. Chem.* **1985**, *24* (12), 1741–1750. https://doi.org/10.1021/ic00206a006.

(25) Gao, X.; Bare, S. R.; Weckhuysen, B. M.; Wachs, I. E. In Situ Spectroscopic Investigation of Molecular Structures of Highly Dispersed Vanadium Oxide on Silica under Various Conditions. *J. Phys. Chem. B* **1998**, *102* (52), 10842–10852. https://doi.org/10.1021/jp9826367.

(26) Gao, X.; Bare, S. R.; Fierro, J. L. G.; Wachs, I. E. Structural Characteristics and Reactivity/Reducibility Properties of Dispersed and Bilayered $V_2O_5/TiO_2/SiO_2$ Catalysts. *J. Phys. Chem. B* **1999**, *103* (4), 618–629. https://doi.org/10.1021/jp983357m.

(27) Ohsaka, T.; Izumi, F.; Fujiki, Y. Raman Spectrum of Anatase, TiO2. *J. Raman Spectrosc.* **1978**, *7* (6), 321–324. https://doi.org/10.1002/jrs.1250070606.

(28) Swamy, V.; Kuznetsov, A.; Dubrovinsky, L. S.; Caruso, R. A.; Shchukin, D. G.; Muddle, B. C. Finite-Size and Pressure Effects on the Raman Spectrum of Nanocrystalline Anatase Ti O2. *Phys. Rev. B - Condens. Matter Mater. Phys.* **2005**, *71* (18), 15–17.

https://doi.org/10.1103/PhysRevB.71.184302.

(29) Nitsche, D.; Hess, C. New Insight into the Structure of Dispersed Titania by Combining Normal-Mode Analysis with Experiment. *Chem. Phys. Lett.* **2014**, *616–617*, 115–119. https://doi.org/10.1016/j.cplett.2014.10.042.

(30) Waleska, P.; Hess, C. Structural Dynamics of Dispersed Titania During Dehydration and Oxidative Dehydrogenation Studied by In Situ UV Raman Spectroscopy. *Catal. Letters* **2018**, *148* (8), 2537–2547. https://doi.org/10.1007/s10562-018-2442-0.

(31) Vuurman, M. A.; Wachs, I. E. In Situ Raman Spectroscopy of Alumina-Supported Metal Oxide Catalysts. *J. Phys. Chem.* **1992**, *96* (12), 5008–5016. https://doi.org/10.1021/j100191a051.

(32) Shapovalov, V.; Fievez, T.; Bell, A. T. A Theoretical Study of Methanol Oxidation Catalyzed by Isolated Vanadia Clusters Supported on the (101) Surface of Anatase. *J. Phys. Chem. C* **2012**, *116* (35), 18728–18735. https://doi.org/10.1021/jp302862q.

(33) Nitsche, D.; Hess, C. Structure of Isolated Vanadia and Titania: A Deep UV Raman, UV-Vis, and IR Spectroscopic Study. *J. Phys. Chem. C* **2016**, *120* (2), 1025–1037. https://doi.org/10.1021/acs.jpcc.5b10317.

(34) Borodko, Y.; Ager, J. W.; Marti, G. E.; Song, H.; Niesz, K.; Somorjai, G. A. Structure Sensitivity of Vibrational Spectra of Mesoporous Silica SBA-15 and Pt/SBA-15. *J. Phys. Chem. B* **2005**, *109* (37), 17386–17390. https://doi.org/10.1021/jp051801x.

(35) Schweitzer, N. M.; Hu, B.; Das, U.; Kim, H.; Greeley, J.; Curtiss, L. A.; Stair, P. C.; Miller, J. T.; Hock, A. S. Propylene Hydrogenation and Propane Dehydrogenation by a Single-Site Zn2+ on Silica Catalyst. *ACS Catal.* **2014**, *4* (4), 1091–1098. https://doi.org/10.1021/cs401116p.

(36) Swamy, V.; Muddle, B. C.; Dai, Q. Size-Dependent Modifications of the Raman Spectrum of Rutile TiO 2. *Appl. Phys. Lett.* **2006**, *89* (16). https://doi.org/10.1063/1.2364123.

(37) Khatun, N.; Anita; Rajput, P.; Bhattacharya, D.; Jha, S. N.; Biring, S.; Sen, S. Anatase to Rutile Phase Transition Promoted by Vanadium Substitution in TiO2: A Structural, Vibrational and Optoelectronic Study. *Ceram. Int.* **2017**, *43* (16), 14128–14134. https://doi.org/10.1016/j.ceramint.2017.07.153.

(38) Zhang, Y.; Harris, C. X.; Wallenmeyer, P.; Murowchick, J.; Chen, X. Asymmetric Lattice Vibrational Characteristics of Rutile TiO2 as Revealed by Laser Power Dependent Raman Spectroscopy. *J. Phys. Chem. C* **2013**, *117* (45), 24015–24022. https://doi.org/10.1021/jp406948e.

(39) Brázdová, V.; Verónica Ganduglia-Pirovano, M.; Sauer, J. Periodic Density Functional Study on Structural and Vibrational Properties of Vanadium Oxide Aggregates. *Phys. Rev. B - Condens. Matter Mater. Phys.* **2004**, *69* (16), 1–14. https://doi.org/10.1103/PhysRevB.69.165420.

(40) Baddour-Hadjean, R.; Smirnov, M. B.; Smirnov, K. S.; Kazimirov, V. Y.; Gallardo-Amores, J. M.; Amador, U.; Arroyo-De Dompablo, M. E.; Pereira-Ramos, J. P. Lattice Dynamics of β-V 2O 5: Raman Spectroscopic Insight into the Atomistic Structure of a High-Pressure Vanadium Pentoxide Polymorph. *Inorg. Chem.* **2012**, *51* (5), 3194–3201. https://doi.org/10.1021/ic202651b.

(41) Waleska, P. S.; Hess, C. Oligomerization of Supported Vanadia: Structural Insight Using Surface-Science Models with Chemical Complexity. *J. Phys. Chem. C* **2016**, *120* (33), 18510–18519. https://doi.org/10.1021/acs.jpcc.6b01672.

(42) Lewandowska, A. E.; Calatayud, M.; Tielens, F.; Bañares, M. A. Dynamics of Hydration in Vanadia-Titania Catalysts at Low Loading: A Theoretical and Experimental Study. *J. Phys. Chem. C* **2011**, *115* (49), 24133–24142. https://doi.org/10.1021/jp204726b.

(43) Magg, N.; Immaraporn, B.; Giorgi, J. B.; Schroeder, T.; Bäumer, M.; Döbler, J.; Wu, Z.; Kondratenko, E.; Cherian, M.; Baerns, M.; Stair, P. C.; Sauer, J.; Freund, H. J. Vibrational Spectra of Alumina- and Silica-Supported Vanadia Revisited: An Experimental and Theoretical Model Catalyst Study. *J. Catal.* **2004**, *226* (1), 88–100. https://doi.org/10.1016/j.jcat.2004.04.021.

(44) Döbler, J.; Pritzsche, M.; Sauer, J. Vibrations of Silica Supported Vanadia: Variation with Particle Size and Local Surface Structure. *J. Phys. Chem. C* **2009**, *113* (28), 12454–12464. https://doi.org/10.1021/jp901774t.

(45) Wu, Z.; Dai, S.; Overbury, S. H. Multiwavelength Raman Spectroscopic Study of Silica-Supported Vanadium Oxide Catalysts. *J. Phys. Chem. C* **2010**, *114* (1), 412–422. https://doi.org/10.1021/jp9084876.

(46) Veige, A. S.; Slaughter, L. G. M.; Lobkovsky, E. B.; Wolczanski, P. T.; Matsunaga, N.; Decker, S. A.; Cundari, T. R. Symmetry and Geometry Considerations of Atom Transfer: Deoxygenation of (Silox)3WNO and R3PO (R = Me, Ph, TBu) by (Silox)3M (M = V, NbL (L = PMe3, 4-Picoline), Ta; Silox = TBu3SiO). *Inorg. Chem.* **2003**, *42* (20), 6204–6224. https://doi.org/10.1021/ic0300114.

(47) Giuli, G.; Paris, E.; Mungall, J.; Romano, C.; Dingwell, D. V Oxidation State and Coordination Number in Silicate Glasses by XAS. *Am. Mineral.* **2004**, *89* (11–12), 1640–1646. https://doi.org/10.2138/am-2004-11-1208.

(48) Sutton, S. R.; Karner, J.; Papike, J.; Delaney, J. S.; Shearer, C.; Newville, M.; Eng, P.; Rivers, M.; Dyar, M. D. Vanadium K Edge XANES of Synthetic and Natural Basaltic Glasses and Application to Microscale Oxygen Barometry. *Geochim. Cosmochim. Acta* **2005**, *69* (9), 2333–2348. https://doi.org/10.1016/j.gca.2004.10.013.

(49) Chaurand, P.; Rose, J.; Briois, V.; Salome, M.; Proux, O.; Nassif, V.; Olivi, L.; Susini, J.; Hazemann, J. L.; Bottero, J. Y. New Methodological Approach for the Vanadium K-Edge X-Ray Absorption near-Edge Structure Interpretation: Application to the Speciation of Vanadium in Oxide Phases from Steel Slag. *J. Phys. Chem. B* **2007**, *111* (19), 5101–5110. https://doi.org/10.1021/jp063186i.

(50) Rossi, T. C.; Grolimund, D.; Nachtegaal, M.; Cannelli, O.; Mancini, G. F.; Bacellar, C.; Kinschel, D.; Rouxel, J. R.; Ohannessian, N.; Pergolesi, D.; Lippert, T.; Chergui, M. X-Ray Absorption Linear Dichroism at the Ti K Edge of Anatase TiO2 Single Crystals. *Phys. Rev. B* **2019**, *100* (24), 245207. https://doi.org/10.1103/PhysRevB.100.245207.

(51) Chen, L. X.; Rajh, T.; Jäger, W.; Nedeljkovic, J.; Thurnauer, M. C. X-Ray Absorption Reveals Surface Structure of Titanium Dioxide Nanoparticles. *J. Synchrotron Radiat.* **1999**, *6* (3), 445–447. https://doi.org/10.1107/S090904959801591X.
